# Precision Molecular Editing: Predicting Substrate Scope and Regiochemistry for CHEESY1, a Flavin Dependent Halogenase

**DOI:** 10.1021/acscatal.5c04436

**Published:** 2025-10-31

**Authors:** Ying Zhang, Olena Holodaieva, Yunpeng Wang, Sunil V. Sharma, Jagwinder Dhaliwal, Keith Mulholland, Danai S. Gkotsi, Rebecca J. M. Goss

**Affiliations:** † EaStCHEM School of Chemistry, University of St Andrews, North Haugh, St Andrews KY16 9ST, Fife, U.K.; ‡ Biomedical Sciences Research Complex, Institute of Engineering, University of St Andrews, North Haugh, St Andrews KY16 9ST, Fife, U.K.; § Chemical Development, AstraZeneca, Silk Road, Business Park, Macclesfield SK10 2NA, U.K.

**Keywords:** halogenase, substrate prediction, regioselectivity, regiochemical prediction, late-stage functionalization

## Abstract

The ability to carry out C–H activation at any site on any heteroaromatic scaffold is a holy grail, offering the potential to revolutionize molecule making. Precision editing, activating, and replacing a C–H bond with a carbon halogen bond opens the way to almost any diversification imaginable. Here, through genome mining and *in silico* analysis, we present a previously undescribed halogenase tool for precision C–H activation and halogenation. While many halogenated metabolites have been found in the marine environment, indicating the operation of a vast array of halogenases, the presence of such halogenases in other salty environments is less well-known. Here, we describe the first discovery and utilization of a halogenase from a microbe associated with salty and fermented food: specifically brined cheese. Most halogenases explored so far have been identified through their association with a biosynthetic gene cluster of a known natural product. Based on their role in the biosynthesis of that natural product, their native substrate is predicted. Many exciting and unexplored halogenases exist discretely of identified biosynthetic clusters. We describe an approach of carrying out halogenase discovery (unrelated to and unguided by known biosynthetic pathways and their encoded natural products) and predicting non-native substrates that the enzyme can and cannot process as well as the regiochemistry of each biotransformation. Following carrying out discovery *in silico*, we demonstrate the validation of the discovery results in the laboratory. CHEESY1 (Chemistry Helper Enzyme Enabling SelectivitY1) is shown to regioselectively halogenate a broad structural range of medicinally relevant heterocycles, including quinolines, isoquinoline, phenylpyrazole, and flavonoids. Being able to predict from gene to substrate to product for this powerful class of enzymes, which together afford the opportunity for providing a general solution for precision molecule editing, ahead of carrying out wet experimentation opens up exciting opportunities for the future of chemical catalysis and synthesis.

## Graphical abstract

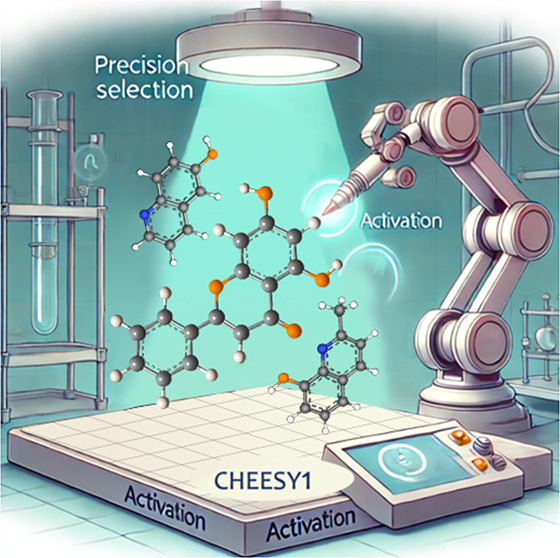

## Introduction

Halogenation of privileged heterocycles is central to the design and development of pharmaceuticals and agrochemicals, enhancing bioavailability and bioactivity as well as introducing a chemically reactive tag for molecule construction.^1,2^ Current synthetic methodologies employ highly reactive electrophiles, and result in challenging-to-separate mixtures of compounds.^3,4^ In contrast, the flavin dependent halogenases (FDHs) can introduce carbon halogen bonds with a high level of regioselectivity.^5–7^

The widely accepted and general mechanism for regioprecise halogenation mediated by FDHs is illustrated in Figure 1. These enzymes are known to generate HOX which is then passaged down a ∼10 Å tunnel to a lysine residue^8^ (Figure 1A). This residue subsequently directs the regiochemistry of halogenation. There are two proposed mechanisms for this next step, one in which the formation and usage of haloamine is implicated^9^ (Figure 1B), and one in which HOX is stabilized and guided through hydrogen bonding, in the case of PrnA to K79 and E346 ^10^ (Figure 1C).

**Figure 1:**
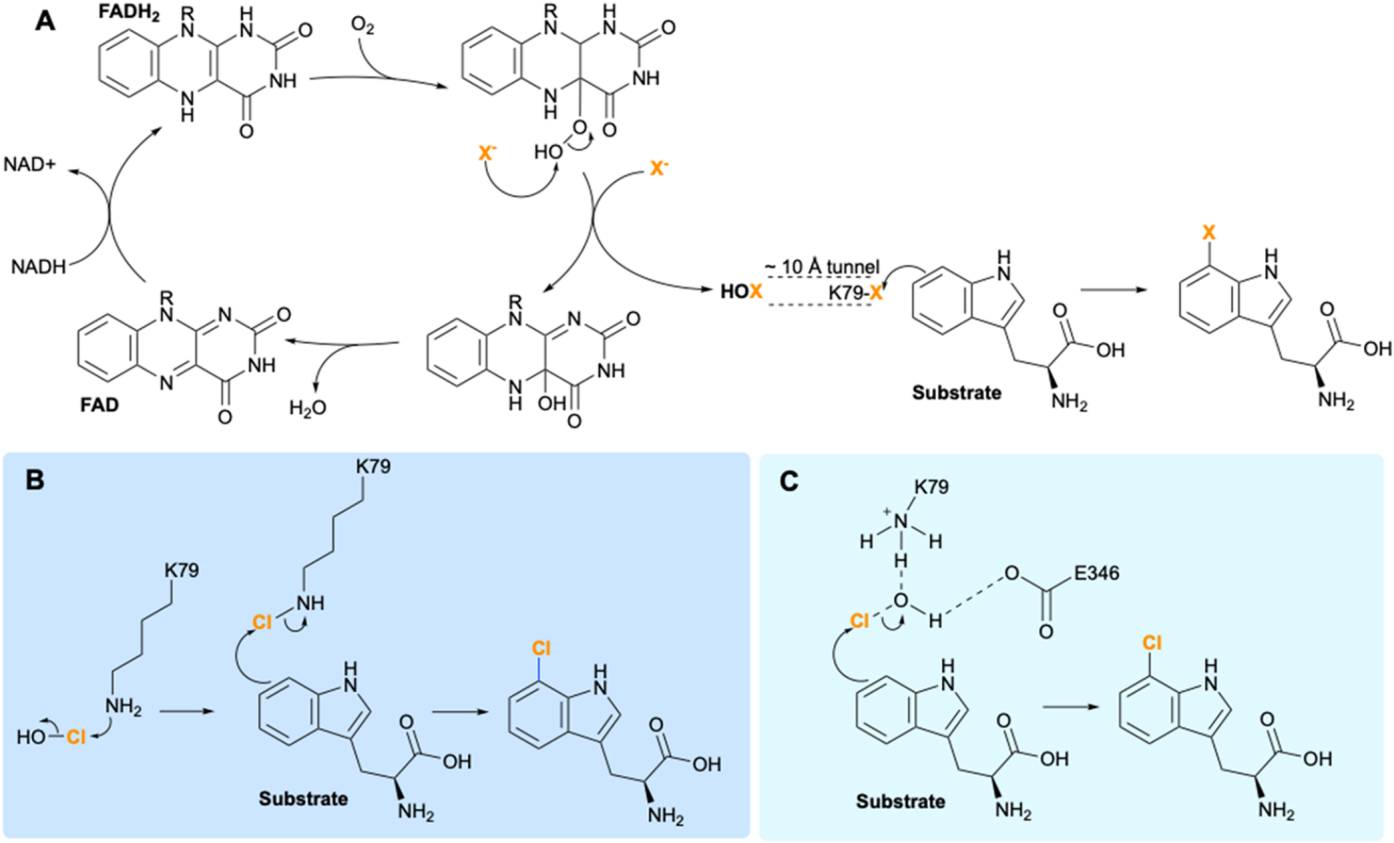
Proposed mechanisms of halogenation for the tryptophan halogenase PrnA. In (A), HOX is generated and passaged down a ∼10 Å tunnel to K79, which subsequently directs the regiochemistry of halogenation through proposed mechanism (B), where formation and usage of haloamine is implicated, or through mechanism (C), where the HOX is stabilized and guided through hydrogen bonding to K79 and E346 of PrnA.

A growing number of FDHs have been shown to possess good levels of substrate promiscuity, while retaining complete regio-precision rendering them potentially interesting tools for development for synthesis,^11,12^ as well as raising questions as to the roles they play in chemical ecology. To find novel halogenases, we are exploring different environmental niches. A proliferation of halometabolites abound within the oceans, isolated from organisms as diverse as sea hares to seaweed.^13,14^ Indeed, native Hawaiians’ favorite edible seaweed, *Asparagopsis taxiformis*, has been shown to contain more than 100 organohalogens.^15^ While numerous halogenases have been found from diverse marine organisms,^16–20^ what about organisms associated with or dwelling within salty and fermented food? Might they also have a propensity to halogenate small molecules?

The majority of halogenases have, so far, been found through the biosynthetic elucidation of halometabolites, and examination of the gene clusters encoding them.^11^ Following the discovery of a halometabolite, the biosynthetic gene clusters, encoding the assembly of the compound, have been sought then explored, and the halogenase and its natural substrate (an intermediate on that biosynthetic pathway) have been investigated. Examples, phylogenetically illustrated in Figure 2, include: AetF^21^ (catalyzing 5,7-dibromination of tryptophan), JamD^22^ (mediating terminal bromination of alkynes), PyrH,^6^ ThaI,^7^ PrnA^23^ (chlorinating tryptophan at C5, C6 and C7, respectively), PrnC^24^ (monochlorination of pyrroles), RadH/Rdc2 (monochlorination/bromination of phenols),^25,26^ PltM (halogenation of phenols)^27^ are all identified from biosynthetic pathways of known natural products. More recently, *in silico-based* discovery methods for new halogenases have been used, mining halogenases from uncurated genomic data sets and directly exploring their ability to halogenate unrelated heteroaromatic compounds of interest.^20,28,29^ Again, when these halogenases are implicated in biosynthetic pathways, the ability to be able to predict what substrates they might utilize is straightforward. Here we describe the *in silico* discovery of a previously unreported halogenase, its structural prediction, molecular docking enabled prediction of substrates that it will and will not transform, and the prediction of regiochemistry of biotransformation.

**Figure 2:**
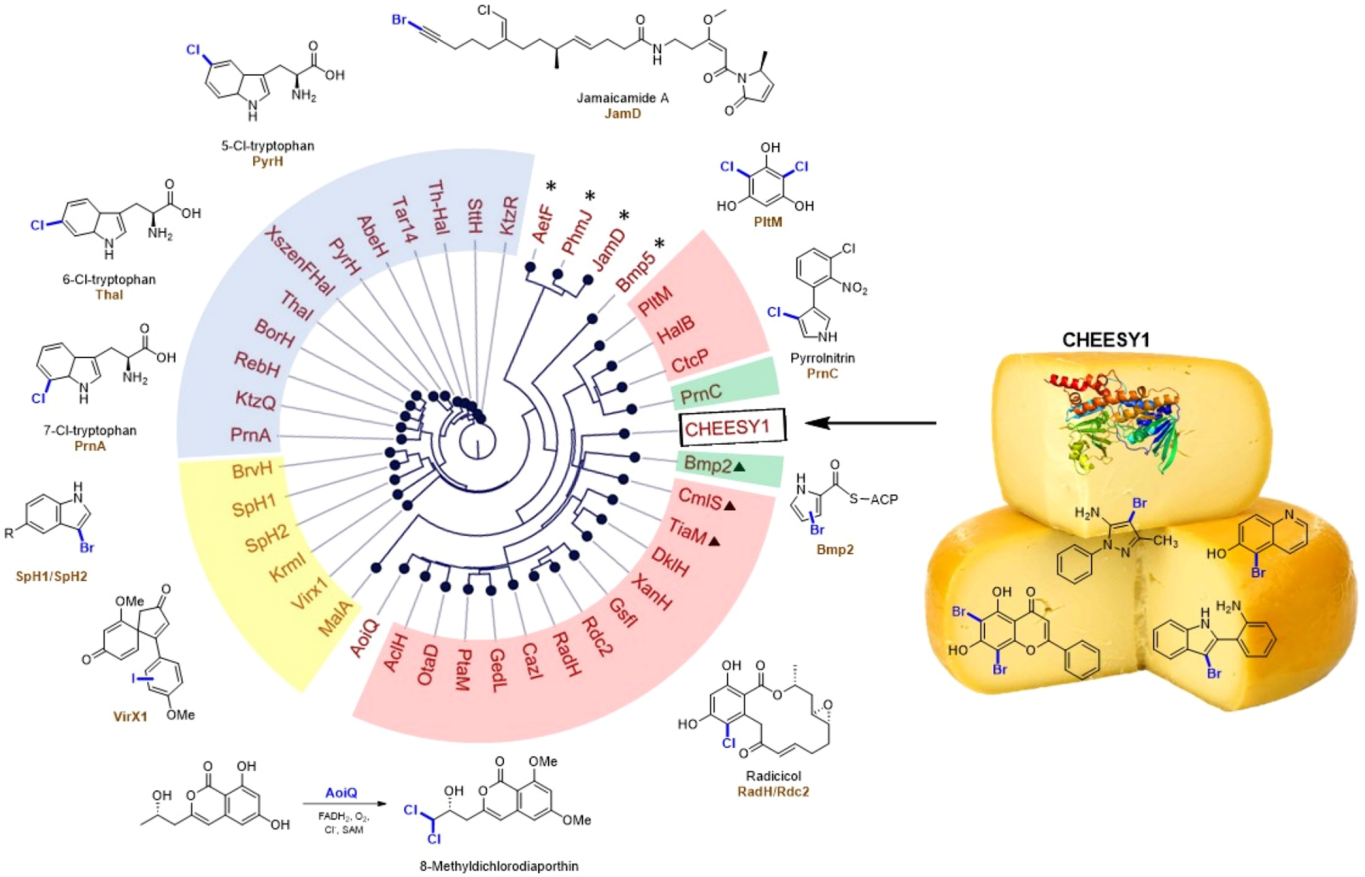
Exemplar FDHs, their phylogenetic distribution, and their substrate utilization, all identified through analysis of the Biosynthetic Gene Clusters (BGCs) encoding halometabolites, except for VirX1, and CHEESY1. Phylogenetic tree positioning CHEESY1 in the context of known FDHs.

## Methods

### Genome Mining for CHEESY1

Genome mining guided by three signature motifs (GxGxxG, WxWxIP, and Fx.Px.SxG) was employed to discover a new and diverse putative FDH. A phylogenetic tree was constructed using a neighbor joining method, then bootstrap 100 by CLC Workbench 22 (CLC Bio-Qiagen, Aarhus, Denmark), followed by global alignment of amino acids using Clustal Omega.^46^ The selected candidate was screened for association with a biosynthetic gene cluster (BGC) by using antiSMASH.

### Docking Simulations

CHEESY1′s structure with the FAD cofactor bound was predicted using AlphaFoldV3. Docking simulations were performed using AutoDockTools-1.5,^47^ employing AutoGrid4 for grid precalculations and AutoDock4 ^48^ for receptor-oriented docking, calculation of ligand binding energy to protein was carried out using AutoVina.^49^

### Construction of the Expression Vector

The gene encoding CHEESY1 was synthesized by GeneArt Gene Synthesis (Thermo Fisher) with codon optimization for *E. coli* BL21­(DE3). It was subsequently cloned into a pET28a-derived vector via *Nde*I and *Hind*III restriction sites to generate the expression construct.

### Protein Production and Purification

The CHEESY1 expression vector was transformed into chemically competent *E. coli* BL21­(DE3). Cells were grown in TB medium supplemented with kanamycin in Erlenmyer flasks and incubated in an orbital shaker incubator at 37 °C. When OD_600_ reached 0.4–0.6, gene expression was induced with 0.5 mM isopropyl β-d-1-thiogalactopyranoside (IPTG). The cells were incubated for a further 28 h at 16 °C. The harvested cells were lysed, and the cleared lysate was purified by Ni-NTA. The column was washed stepwise with buffers containing 20 mM, 40 mM, and 50 mM imidazole, and the target protein was eluted with 250 mM imidazole. The eluate was dialyzed with storage buffer (50 mM HEPES, 10% glycerol, pH 8.0). Further purification was performed by size-exclusion chromatography (SEC) using a HiLoad 16/600 Superdex 200 pg column (Cytiva). Fractions containing pure CHEESY1, as verified by sodium dodecyl sulfate-polyacrylamide gel electrophoresis (SDS-PAGE) and protein mass spectrometry (Mass spectrometry and proteomics facility, University of St Andrews), were pooled and concentrated using Amicon Ultra-15 centrifugal filters. The flavin reductase PrnF was produced and purified in the same manner.

### Enzymatic Halogenation

The initial halogenation reactions were carried out on a 100 μL scale in a V-bottom 96-well plate (Greiner Bio-One) containing the following components: 50 μM CHEESY1, 1 μM PrnF, 10 μM FAD, 2.5 mM NADH, 10 mM NaCl/NaBr, 500 μM substrate, 200 U/mL catalase, and 10 U/mL SOD (Table S6). After overnight incubation at 30 °C with shaking at 150 rpm (New Brunswick Innova 44, orbit 2.5 cm), the reactions were quenched with an equal volume of methanol. The clarified reaction mixtures was analyzed initially using UPLC, followed by LC-MS for confirmation of products. Large-scale biotransformation was performed using the same reaction concentrations in multiple parallel 1 mL batches (final volume was 10 mL-50 mL depending on the conversions), which were combined later for product purification. Process parameters were optimized when significant reduction in conversion efficiency was observed.

### MCD Test

For MCD assays, 3 conditions were set up: Assay 1: MCD + standard halogenation components excluding the substrate. Assay 2: MCD + standard halogenation components including substrate. Assay 3: MCD + all components except CHEESY1 and the substrate. Reactions (100 μL final volume) were performed in 96-well plates, as described. After overnight incubation, reactions were quenched with MeOH and analyzed by UPLC as described previously. Peak area of the MCD was measured under 270 nm.

### Chemical Bromination Using NBS or NaOBr

Reactions were carried out in 100 μL systems containing 1 mM substrate and brominating agents (NBS or NaOBr) at 1 or 5 mM (corresponding to 1 or 5 equiv). After 3 h of incubation at room temperature, the reactions were quenched with an equal volume of methanol and analyzed by UPLC. The chromatograms from chemical halogenation and enzymatic halogenation were compared to evaluate reaction profiles.

### Product Characterization

Products were extracted by washing the reaction mixtures with ethyl acetate for 3 × 10–50 mL (the final volume of large-scale biotransformation),. The crude extract was purified by normal phase chromatography using a silica column (10 g) (100% hexane to 100% DCM, 20 to 30 column volumes), or reverse phase chromatography (Biotage Sfär C18 D - Duo 100 Å 30 μm, gradient from pure H_2_O to 100% MeOH, over 30 column volumes). The dried product was dissolved in appropriate deuterated solvents (CDCl_3_ or CD_3_OD) and analyzed by ^1^H NMR and ^13^C NMR.

### Kinetics

The kinetic assays were performed in triplicate. Each 100 μL reaction contained 25 μM CHEESY1, 1 μM PrnF, 10 μM FAD, 2.5 mM NADH, 10 mM NaCl/NaBr, substrate (ranging from 10 μM to 10 mM), 200 U/mL catalase, and 10 U/mL SOD in 50 mM HEPES buffer (pH 8.0). Reactions were quenched at multiple time points; the corresponding rates of product formation were determined, and the resulting initial velocities and a range of substrate concentrations were fitted to the Michaelis–Menten model using Prism 9.4.0 (GraphPad Software) to derive kinetic parameters.

### Thermal Shift Assay and Thermostability Test

Different protein concentrations (2, 4, 6, and 8 μM) were combined with Sypro Orange protein dye at 1×, 2×, 3×, and 4× concentrations in a total reaction volume of 25 μL. Fluorescence signals from denatured proteins were monitored using a real-time PCR system (Stratagene Mx3005P with MxPro v4.01 software) across a temperature gradient from 25 °C to 95 °C, with a ramp rate of 0.5 °C/min.

For the thermostability test, Th-Fre,^50^ previously reported as a thermostable reductase with an optimum temperature of 45 °C, was employed as the reductase component. Reactions were performed using substrate **1** across a temperature gradient of 25 °C, 30 °C, 35 °C, 40 °C, and 45 °C (Tm of CHEESY1), with PrnF used as the reductase in parallel assays for comparison.

### Site-Directed Mutagenesis

Based on docking simulation results implicating potential residues involved in the substrate binding site, Y170I, Y170 V, S311I, S311 V, and S311A mutants were constructed to probe residue-specific roles. Mutagenesis was performed according to the protocol by Liu et al.,^51^ detailed experimental description can be seen in Section S2.14.

## Results and Discussion

In our search for new flavin dependent halogenases from diverse origins for application in biotechnology and medicinal chemistry, we have employed the recently identified conserved halogenase motif, *Fx.Px.Sx.G*,^20^ to explore uncurated sequence space. We have observed halogenases that contain this motif to be present in every kingdom of life. In this manner, CHEESY1 was found, encoded in the genome of *Chromohalobacter japonicus*, an organism previously found in Danish cheese brine^30^ and Japanese salty food^31^ (multiple sequence alignment is shown in Figure S1). Sequence alignment showed that both the essential *GxGxxG* (motif 1) and *WxWxIP* (motif 2) motifs were present. The flavin binding motif *GxGxxG* is very well-defined for both flavin monooxygenases (FMOs)^32–35^ and flavin dependent halogenases (FDHs),^23–27^ for an overview of flavin binding within such enzymes please see Kubo et al.^36^, Kleiger et al.^37^, and Crowe et al.^11^ From phylogenetic analysis, CHEESY1 was positioned within the phenol-FDH clade, occupying a distinct subclade of bacterial origin (Figure 2). Intriguingly, it appears to neighbor Bmp2 ^38^ (43%/28%, similarity/identity) on one side and Rdc2 (44%/26%, similarity/identity) on the other side. Bmp2 is a variant B FDH halogenase responsible for the tetrabromination of a peptidyl carrier protein tethered to pyrrole, while Rdc2 was identified from the biosynthesis of radicicol, a variant A halogenase capable of accepting and regioselectively halogenating sterically bulky phenolic substrates. From its phylogenetic position, we postulated that CHEESY1 might have the capability to process a broader range of heteroaromatic substrates. Due to the potential context of CHEESY1 within a microbe associated with a variety of salty and fermentative foods, we searched for any putative BGCs using antiSMASH^39^ and found that CHEESY1 is not embedded within a BGC.

With the aim of exploring CHEESY1 as a potential tool to enable precision molecule editing around med chem relevant scaffolds, we then set out to explore whether accurate substrate prediction, using *in silico* methods, might be possible. It is becoming normal practice to explore the docking of substrates that have already been shown to be processed by an enzyme. We postulated that if we could build a sufficiently accurate model and incorporate careful spatial, geometric, and energetic cut offs, we would be able to use it predictively for halogenases. With sequence space increasing at a rapid rate this could potentially provide a very useful approach for informing on appropriate enzyme: substrate combinations for selecting optimal enzymes for a given transformation ahead of carrying out any wet experiments (Figure 3). A particular challenge with the flavin dependent halogenases that has previously precluded the utilization of this approach for predicting which substrates might be transformed is that precise geometric positioning of potential substrates relative to the anchored halogenating species is required. We postulated that with a suitably carefully prepared model and reactivity informed docking this challenge could be overcome.

**Figure 3:**
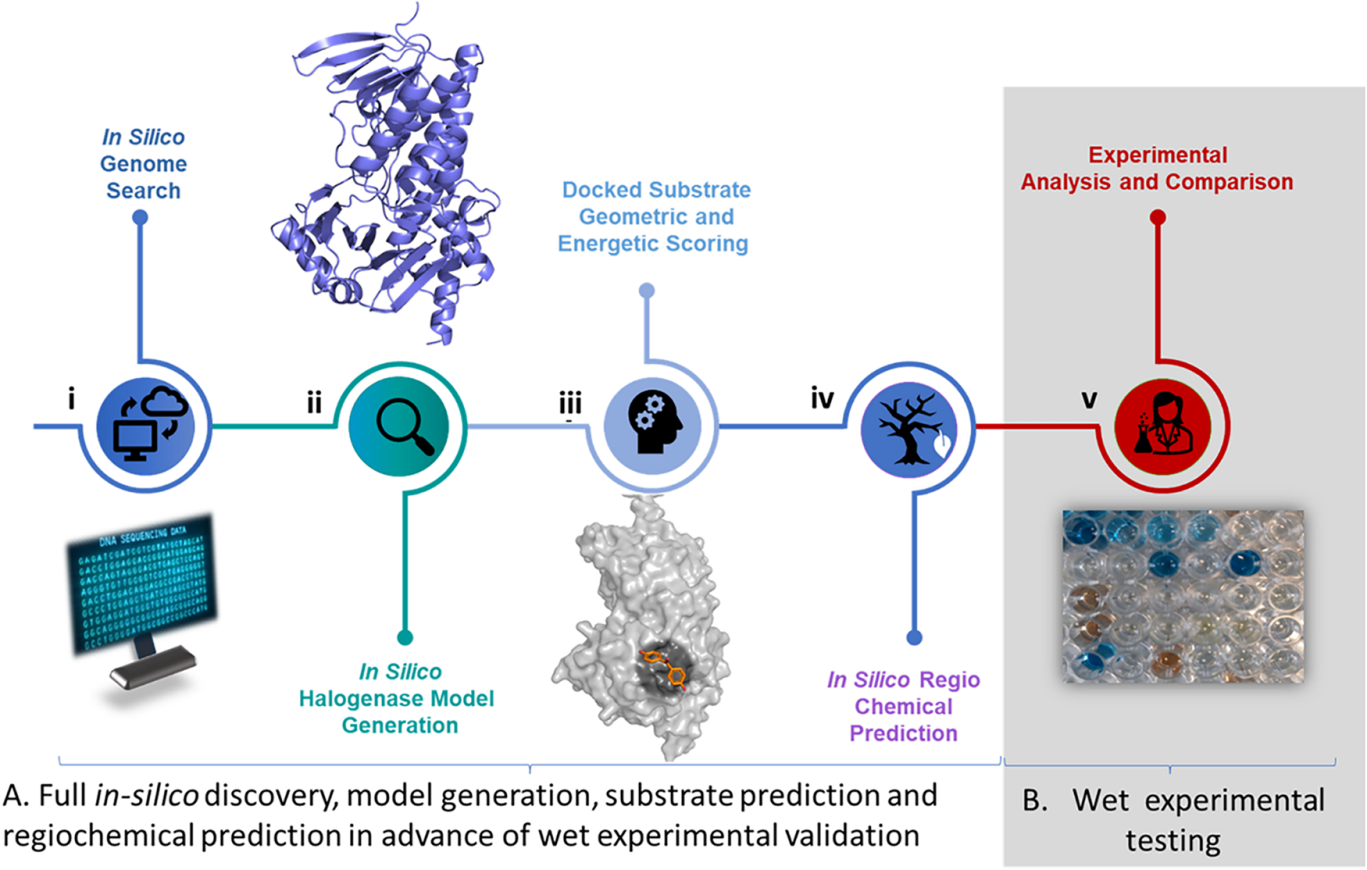
An approach of (A) *in silico* discovery, followed by (B) wet validation: (i) use of 3 motifs and phylogeny screen in halogenase identification and selection, (ii) generation of comparative enzyme models using AlphaFoldV3, (iii) molecular docking of 53 compounds of interest: prediction of their likelihood to be a substrate, (iv) regiochemical prediction, and (v) experimental verification.

To gain insight into the potential non-native substrates that CHEESY1 might halogenate, we next used AlphaFoldV3 ^40^ to model the likely structure of the enzyme. Following careful prediction of its substrate binding site, we used flexible docking models to investigate potential enzyme: substrate interactions.

### Predicting the Substrate Binding Site

In order to determine this putative substrate binding site, we computationally mapped the entire cavity surface of the enzyme, analyzing it comparatively against crystal structures of known FDHs (Figure S6). We identified four potential pockets for substrate binding (Figure S2, shown as P1, P2, P3, and P4).

To determine the most plausible pocket we iteratively used cycles of rigid and flexible docking simulations to sample possible binding poses. We comprehensively established distance between K79 & substrates alongside the corresponding binding energies (Figures S4 and S5 and Table S4). P2 emerged as the only location that simultaneously satisfied the criteria of proximity to catalytic residues and the predicted HOX tunnel with its associated residues (Figure S3), as well as computationally yielded negative binding energies with potential substrates (Table S4).

From our analyses, we could see the substrate binding site (P2) to be located at the bottom of a deep active site cavity with the potential to accept large substrates (Figure 4A). This deep cavity contained both *WxWxIP* (motif 2) and *Fx.Px.Sx.G* (motif 3). The flavin binding site *GxGxxG* (motif 1) could be seen to be close to the entrance of the cavity (Figure 4B). Most flavin dependent halogenases reported so far have separate entrance channels, into the enzyme, for cofactor and substrate (Figure 4D, Rdc2 a phylogenetic neighbor is illustrated here as an example). In the case of predicted model CHEESY1, the entrance channel for cofactor and substrate can be seen to be shared (Figure 4C). The putative substrate binding site for CHEESY1 could be seen to consist of: I50, G51, S53, L55, K79 (responsible for HOX stabilization and positioning), Y170, *W224* (second W in motif 2), *I226* (I in motif 2), S234, *F305* (F in motif 3), L306, N307, *P308* (P in motif 3), *F310*, *S311* (S in motif 3), and D307 positioned to costabilize HOX with K79.

**Figure 4:**
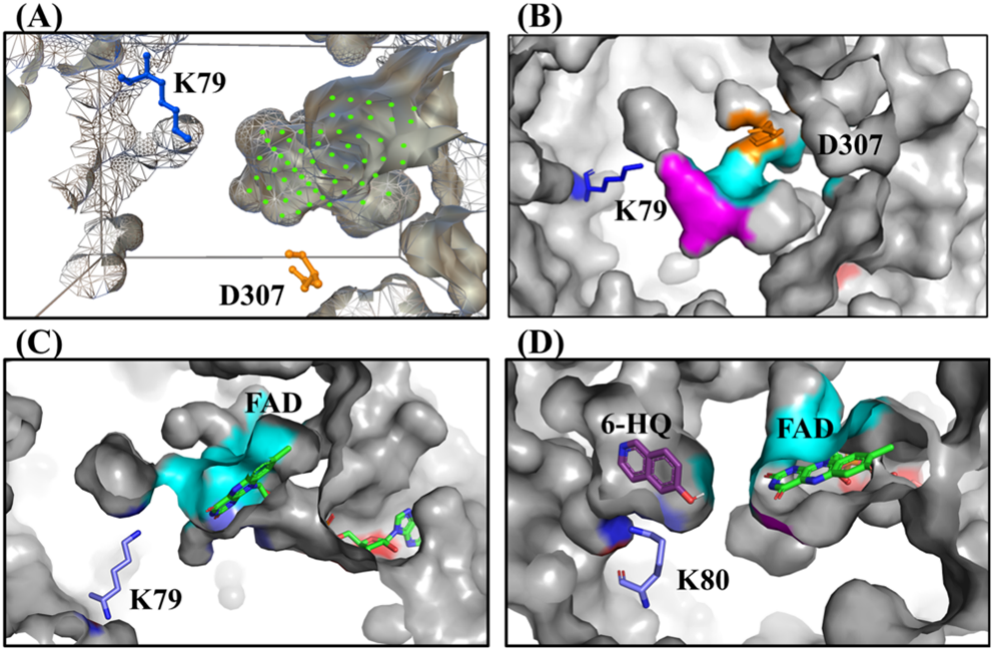
Structural overview of the CHEESY1 substrate binding site showing a large cavity and a hydrophobic binding region enabling the controlled presentation of substrates for halogenation. (A) Positions of substrates binding within the putative binding region in P2 are indicated with green dots in accordance with conventions; (B) Close up of the active site cavity of CHEESY1; (C) Substrate binding site of CHEESY1 showing the shared entrance channel of substrate and the cofactor FAD; (D) Substrate binding site for closest phylogenetic neighbor Rdc2 showing separate entrance channels for substrate 6-hydroxyisoquinoline and cofactor FAD, with 6-hydroxyisoquinoline (6-HQ) shown as a purple stick. The above structural models were predicted by AlphaFoldV3. Color scheme: catalytic lysine (K79 & K80) in blue, D307 in tangerine, FAD in green, *GxGxxG* (motif 1) in pink, *WxWxIP* (motif 2) in magenta, *Fx.Px.Sx.G* (motif 3) in cyan.

### Computationally Predicting Non-Native Substrates

We predicted potential substrates for CHEESY1 by first inspecting the sterics of their access, through the substrate channel, to the depths of the active site. Based on the large size of the active site cavity, we selected 53 modestly sterically bulky biaryl compounds to investigate through docking. We then assessed the candidate substrate’s ability to bind to within the P2 pocket of the active site, and to be presented by this pocket with appropriate proximity and geometry to the anchored electrophile. (Multiple explored binding positions within P2 are represented by green dots in Figure 4A). In this manner, and using a combination of binding energy and proximity and orientation to the halogenating species, we were able to predict the likely candidacy of unnatural substrates to be processed by the enzyme (Table S5).

Compounds that bound too weakly (less than −20.92 kJ/mol) were considered to have a too low affinity for the active site. It was also considered that compounds with very large binding energies (more than −35.56 kJ/mol) might bind too tightly to the enzyme and result in poor conversions. Binding energies between −20.92 to −35.56 kJ/mol were considered optimal. Of the 53 selected challenge substrates, 7 were considered to be potential substrate candidates.

### Computationally Predicting the Regiochemical Outcome

For six of the seven substrates identified *in silico*, a single binding possibility was identified (Figures S7–S11 and S13). To explore whether we could predict the regiochemical outcomes, we then examined the proximity and orientation of the lysine, responsible for the regiocontrolled delivery of the electrophilic halogen to the substrate. From these studies, we could predict the likely regiochemistry of halogenation (Figures S7–S19).

Having predicted these *in silico* identified compounds to have potential as substrates for CHEESY1, we set out to explore experimentally whether these predictions might be correct. The gene encoding CHEESY1 was synthesized and codon optimized (Figure S20), then cloned into a derivative pET28a expression vector with an *N*-terminal 8His tag. Both CHEESY1 and the reductase PrnF were heterologously produced in *Escherichia coli* BL21­(DE3), and purified by nickel-nitrilotriacetic acid (Ni-NTA) affinity chromatography (Figure S21). We tested all 53 compounds, only the 7 compounds that were identified to be promising unnatural substrates were seen to be halogenated by the enzyme, in initial small scale assays, with good to excellent initial conversions (see Figures S22–S29 for ultraperformance liquid chromatography (UPLC) chromatograms and Figures S30–S37 for corresponding liquid chromatography–mass spectrometry (LC-MS) data). These results align consistently with our predictions. Monochlorodimedone (MCD) was used to monitor potential HOX leakage during the reaction period in the presence and absence of substrate (for experimental details, see Section S2.7). No significant MCD consumption was observable, indicating there to be no HOX leakage (Figure S38). After confirming the enzyme’s ability to process these substrates, reactions were scaled up for each substrate with appropriate optimization, to provide sufficient material for full characterization (one exemplar optimization see Table S8). The products were extracted and purified, and the regiochemistry of halogenation was determined by analysis of the ^1^H and ^13^C NMR spectra for each compound (Figures S43–S63 for all compounds ^1^H and ^13^C NMR spectra). During reaction optimization, we also investigated the thermal stability of CHEESY1. The enzyme may be seen to have a melting temperature (Tm) of 45 °C (Figure S64) and an optimal reaction temperature of 30 °C (Figure S65).

In addition to all of the predicted putative substrates being processed by CHEESY1 with moderate to excellent conversions, the regiochemistries for halogenation predicted through modeling were also experimentally validated (Figure 5A). Single regioselective halogenation was observed for 6-hydroxyquinoline (**1**), 5-amino-3-methyl-1-phenylpyrazole (**2**), 2-(1*H*-indol-2-yl)­aniline (**3**) and 2-methyl-8-quinolinol (**4**). As predicted through modeling (Figures S11 and S12), C2 symmetric 4,4′-dihydroxybenzophenone (**5**) was symmetrically halogenated. In addition to monobromination, a 10% conversion to the dihalogenated product (**5b**) was observed for this large, inflexible, substrate.

**Figure 5:**
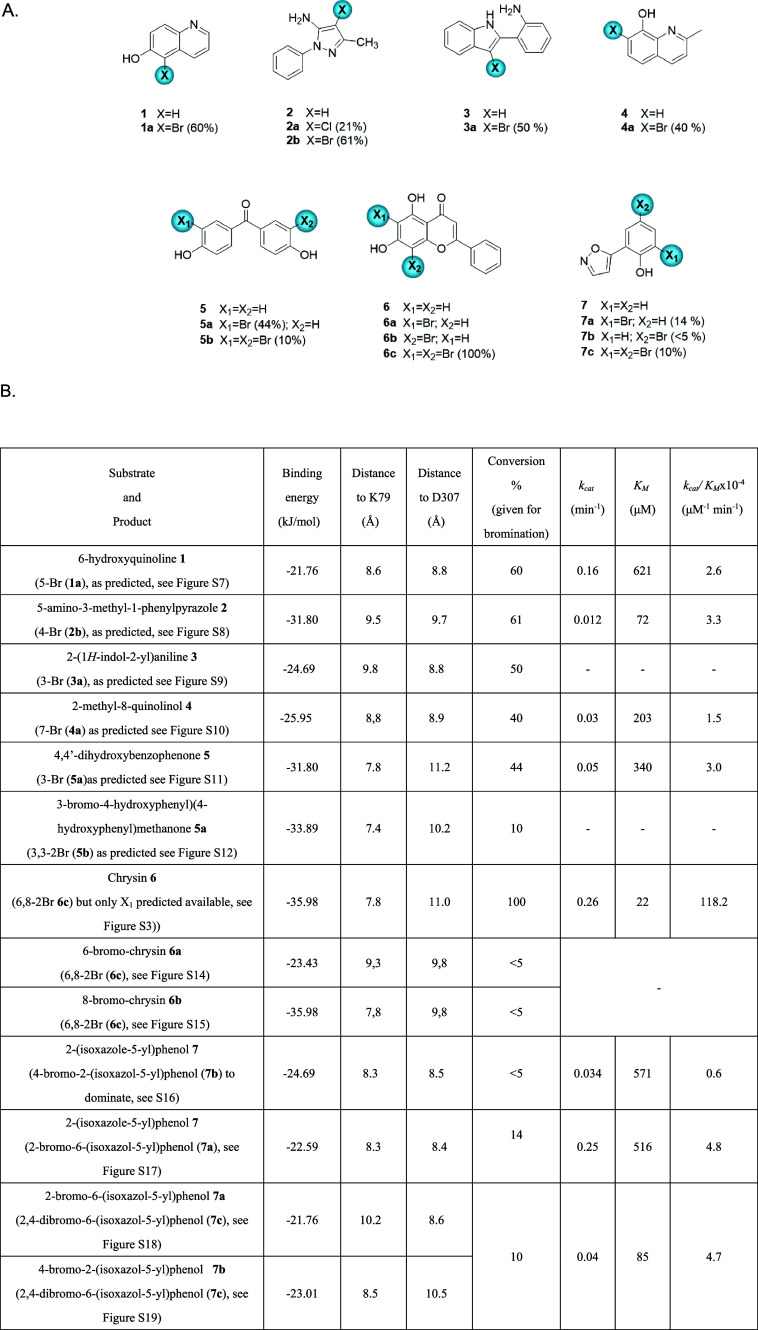
(A) Non-native substrates of CHEESY1, predicted *in silico*. The regiochemistries for halogenation accurately predicted through modeling are experimentally validated. (B) Tabulation of *in silico* predictions for binding energetics and proximity to catalytic K79 and D307 residues, presented alongside experimentally observed conversions, regiochemistries, and kinetics. For the laboratory experimental data, in order to obtain as unbiased data as possible, analytical enzymatic reactions are carried out in unoptimized form, with initial conversions recorded here (to enable scale up, and compound purification, reaction conditions were then optimized). UPLC conversions were measured at specific wavelengths to optimize peak detection: 254 nm for substrates **1** and **4**, 245 nm for **2**, and 300 nm for **3**, **5**, **6**, and **7**. UPLC conversion (%) represents the % of product peak area at the specified wavelength.

Flavin dependent halogenases usually proceed with exquisite regioselectivity, as observed here for substrates **1–4**. However, two different low energy binding orientations with very similar binding energies could be seen for 2-(isaxozol-5-yl)­phenol **7** (Figure 5B, −24.69 kJ/mol vs −22.59 kJ/mol), leading us to predict the generation of two regioisomers. This was indeed observed experimentally with 14% conversion to **7a** (ortho-halogenation) and 5% to **7b** (para-halogenation). Additionally, 10% of the dihalogenated product (**7c**) was obtained. These experimental results are in accordance with the docking results, and the monobrominated compounds are both seen to be good substrates for CHEESY1. As shown in Figure 5B, the distance of **7a** to K79 (10.2 Å) is much further than that of **7b** to K79 (8.5 Å). The closer proximity of **7b** to K79 facilitates the second halogenation to form **7c**, leading to the consumption of **7b** and higher accumulation of **7a**. Only one binding orientation (C6) was predicted for flavonoid chrysin **6** (Figure S13). The monobrominated chrysin may be calculated to be an excellent substrate, rotating in the active site, facilitating rapid dibromination. In accordance with this, experimentation revealed that only a very small trace of a monobrominated species was present at any time, with this being rapidly converted to the dihalogenated compound (**6c**). It was thus not possible to isolate any monobrominated material to verify the experimentally predicted first position of halogenation.

Chemical halogenating agents, such as *N*-bromo succinimide (NBS) react with the most nucleophilic sites of a heterocycle, often generating mixtures of products. For flavin dependent halogenases, the site of halogenation is dictated by the enzyme’s positioning of the substrate with respect to its catalytic lysine, sometimes this is indeed the most nucleophilic position and sometimes this differs. To enable comparative analysis we carried out the reaction of compounds **1**-**6** with both NaOBr and NBS each at 1 and 5 equiv (see Table S7 for experimental setup and results). Surprisingly, while CHEESY1 demonstrated good levels of bromination with 2-(1*H*-indol-2-yl)­aniline (**3**), 2-methyl-8-quinolinol (**4**) (50, 40% respectively), the comparable reactions with conventional halogenating agents showed maximum conversions of 7%, 5%. Similar trends can be seen for chrysin (**6**) (Figure S41). For 5-amino-3-methyl-1-phenylpyrazole (**2**) and 4,4′-dihydroxybenzophenone (**5**), 5 equiv of NBS achieved 100% different regioisomers (Figures S39 and S40), illustrating further the utility of CHEESY1 over a conventional brominating reagent.

CHEESY1 was demonstrated to be capable of bromination and chlorination but not iodination; however, the enzyme shows considerable preference for bromination, as reinforced by the observed kinetics (Figures 5B and S42). While chlorination of 2-(1*H*-indol-2-yl)­aniline (**3**) proceeds with a modest 21% conversion, for other substrates, <1%–6% of the chlorinated product was observed. Notably, not only is organic substrate and regiochemical prediction accurate, but a strong correlation may be seen between the binding energetics, and the conversion levels and kinetics. Together, the sterics of access of the substrate into the active site, its ability to bind well, but not too tightly to the binding pocket, and a distance below 11.5 Å between the C–H to be activated and D307, are all seen to be important. However, along with the ability of the substrate to access the active site for CHEESY1, the next most important factor seems to be the proximity of the lysine that orientates and delivers the halogenating species. For chrysin, all of these factors come together with the lysine perfectly positioned and orientated 7.8 Å from a well bound substrate (−35.98 kJ/mol), resulting in an observed *k*_cat_/*K*_M_ *of* 118.2 × 10^–4^ μM^–1^ min^–1^ and 100% conversion (This is comparable with KtzQ’s processing of its natural substrate tryptophan with *k*_cat_/*K*_M_ *of* 120 × 10^–4^ μM^–1^ min^–1^).^41^ It is likely that after the formation of the first C–X bond, monobrominated chrysin remains and rotates in the active site, facilitating rapid dibromination (Figures S14 and S15). 4,4′-dihydroxybenzophenone (**5**) is calculated to have a similar binding energy to chrysin, within the active site (−31.80 kJ/mol vs −35.98 kJ/mol respectively). The higher steric volume of the former is likely to limit its rate of diffusion into the active site. Once halogenated, its high steric volume will make it challenging for the molecule to rotate within the active site and will likely have to exit and then reenter the active site in order to be dihalogenated.

Many of the modeled, putative substrates displayed particularly close interactions with amino acid residues I50, G51, Y170 and *W224*, *I226*, as well as *F305*, *P308*. To investigate key residue-substrate interactions, we selected Y170 and S311 for further study, as these two residues consistently demonstrated interactions with all tested substrates. The mutants Y170I, Y170 V, S311I, S311 V, and S311A were constructed and produced in *E. coli* BL21­(DE3), all of which were obtained in the soluble form. After Ni-NTA purification (Figure S66), these five mutants were tested against substrates **1**, **2**, and **6**. S311I resulted in a complete loss of enzymatic activity, while S311A showed a moderate effect on activity with no effect on substrate **2** halogenation. Y170I affected the activity more significantly than Y170 V (Figure S67). These results suggest that steric hindrance, within the mutants, prevent effective substrate binding.

## Conclusions

We demonstrate the power of using an *in silico* approach from enzyme discovery through to enzyme structural prediction and flexible docking to predict suitable substrates for a previously unreported flavin dependent halogenase. A strong correlation exists between enzyme: substrate binding energies and the kinetics and conversions of the enzyme-catalyzed reactions. Notably, we also demonstrate the ability to use *in silico* modeling to accurately predict the regiochemistry of halogenation. This will be a particularly powerful approach, paving the way for the selection and use of an individual halogenase *in silico* to give exclusive access to the desired single-regioisomer desired. Such an approach, enabling precision molecule editing, will have broad reaching implications, not least in late-stage functionalization for medicinal chemistry. CHEESY1, a novel FDH from a microbe associated with salty food, has been shown as a promising biocatalyst with capability to carry out regioselective mono- and dibromination of a range of phenolic and indolic derivatives. It is unusual, among halogenases reported so far, in having a single entrance point to the active site shared by both the substrate and the cofactor. Multiple bromination is commonly seen for bromoperoxidases (BPOs) but it is rarer for FDHs. Dichlorination has been seen to be mediated by two-component halogenases such as, Rdc2, MalA,^42^ PltA,^43^ and DklH,^44^ but a larger active site cavity is required to enable dibromination such as that seen for CHEESY1 and for the single component halogenases Bmp5 ^45^ and AetF. AetF is particularly notable with its large active site cavity enabling not only dibromination but also di-iodination of L-tryptophan. With ever increasing access to genome sequences, the opportunity to find halogenases capable of late-stage C–H activating and halogenating any aromatic at any desired position will become a reality. The ability to identify a previously unexplored halogenase *in silico* then accurately predict the substrates that it will process, as well as the regiochemistry of biotransformation, ahead of any wet experimentation, is a powerful step toward realizing the goal of being able to late-stage C–H edit any molecule at any position.

## Supplementary Material



## Notes

Full experimental details, in silico analyses, and compound characterization. (PDF)
