# Supplementary material for: Precision Molecular Editing: Predicting Substrate Scope and Regiochemistry for CHEESY1, a Flavin Dependent Halogenase
Source: ACS Catal. 2025 Oct 31;15(22):18995–9004. doi: 10.1021/acscatal.5c04436 (PMC12645471; doi:10.1021/acscatal.5c04436)
Supplement: Supplementary file 1 [file cs5c04436_si_001.pdf]

Precision Molecular Editing: Predicting Substrate Scope and Regiochemistry for CHEESY1 a Flavin Dependent Halogenase

Authors: Ying Zhang,[1S†§] Olena Holodaieva,[1π] Yunpeng Wang,[1Δ] Sunil V. Sharma[1θ], Jagwinder Dhaliwal,[1,2†] Keith Mulholland,[3] Danai S. Gkotsi[1,2] and Rebecca J. M. Goss*[1]

[1]EaStCHEM School of Chemistry, University of St Andrews, North Haugh, St Andrews, Fife, KY16 9ST, UK. Biomedical Sciences Research Complex, Institute of Engineering, University of St Andrews, North Haugh, St Andrews, Fife, KY16 9ST, UK.

RJMG@st-andrews.ac.uk

[2] These authors contributed to this work whilst studying at the University of St Andrews

[3] Chemical Development, AstraZeneca, Silk Rd, Business Park, Macclesfield SK10 2NA, UK.

[† §]These authors carried out the *in silico* discovery of the enzyme and demonstrated initial heterologous production. [π]This author analysed compound preference *in silico* and contributed to the selection of compounds for the assaying of the halogenase. [S]This author carried out the full biochemical investigation, producing the enzyme, carrying out substrate screening, reaction improvement, scale up, product isolation and product characterisation. [Δ]This author designed the mutagenesis primers and constructed the mutant expression vectors. [θ]This author carried out the chemical halogenation experiment.

Supporting Information

Catalogue

1. General Materials 1
2. Methods 1
2.1 General Methods 1
2.2 Docking Simulations of CHEESY1 2
2.3 CHEESY1 Expression Vector Construction 2
2.4 Production and Purification of CHEESY1 and PrnF 2
2.5 Protein Identification by SDS-PAGE and MS 3
2.6 Small-Scale Halogenation Assays 3
2.7 Monochlorodimedone (MCD) assays 3
2.8 Chemical Bromination Using N-Bromosuccinimide (NBS) or Sodium Hypobromide (NaOBr) against All Substrates 4
2.9 Large Scale Halogenation Assays 4
2.10 Kinetics 4
2.11 Product Purification and Identification 5
2.12 Thermal Shift Assay 5
2.13 Thermal Stability Assay of CHEESY1 with Th-Fre and PrnF 5
2.14 Site-Directed Mutagenesis 5
3 Supporting Results 7
3.1 Sequence Alignment Results of CHEESY1 7
3.2 Docking Simulations of CHEESY1 with Selected Substrates 9
3.2.1 Procedure for Substrate Binding Site Determination 9
3.2.2 Application of Substrate Molecular Docking to Selected Substrates 16
3.3 Gene String Information of CHEESY1 30
3.4 SDS-PAGE of CHEESY1 and PrnF 32
3.5 UPLC Chromatograms of Substrate **1** to **7** 32
3.6 LC-MS Interpretation of Halogenation Reactions 41
3.7 MCD Assays 51
3.8 Chemical Bromination Using NBS or NaOBr 51
3.9 Preliminary Assay Optimisation 54
3.10 Kinetics of CHEESY1 56
3.11 Product Characterisation and NMR Spectra 57
5-Bromo-6-hydroxyquinoline (1a) 57
4-Chloro-3-methyl-1-phenyl-1H-pyrazol-5-amine (2a) 61
4-Bromo-3-methyl-1-phenyl-1H-pyrazol-5-amine (2b) 64
2-(2'-Anilino)-3-bromo-1H-indole (3a) 67
7-Bromo-2-methylquinolin-8-ol (4a) 70
(3-Bromo-4-hydroxyphenyl)(4'-hydroxyphenyl)methanone (5a) 73
Bis(3-bromo-4-hydroxyphenyl)methanone (5b) 76
6,8-Dibromo-5,7-dihydroxy-2-phenyl-4H-chromen-4-one (6c) 78

2-Bromo-6-(isoxazol-5-yl)phenol (7a) 81

4-Bromo-2-(isoxazol-5-yl)phenol (7b) 84

2,4-Dibromo-6-(isoxazol-5-yl)phenol (7c) 87

3.12 Thermal Shift Assay of CHEESY1 90

3.13 Thermal Stability Analysis of CHEESY1 90

3.14 Halogenation Activity Assays of Five Mutants against Selected Substrates 91

4 References 92

1. General Materials

Experimental consumables, organic solvents, chemical reagents, compounds, enzymes used in this work were purchased from Sigma Aldrich, Alfa Aesar, Fisher, Griener Bio-One, Formedium, Promega, Invitrogen or Melford Scientific. 10-200 kDa Protein Ladder was purchased from NEB. NuPAGE™ MES SDS Running Buffer (20X) was purchased from ThermoFisher. Bis-Tris Mini Protein Gels, 4-12% was purchased from ThermoFisher. Amicon® Protein centrifugal filter (30 kDa cutoff) and dialysis tubing (12-14 kDa cutoff) were purchased from Sigma Aldrich. Sypro orange dye was purchased from Invitrogen. MicroAmp™ Fast Optical 96-Well Reaction Plate was purchased from ThermoFisher. Excel Scientific ThermalSeal RT™ sealing film was purchased from ThermoFisher. Miniprep Kit and clean-up kit were purchased from Promega.

2. Methods

2.1 General Methods

Microbial work was performed in a Faster BH-EN class II vertical laminar airflow cabinet or next to a Bunsen flame. DNA sequencing was performed by GATC Biotech. Sterilisation of media was performed by autoclaving at 134 °C for 20 min unless otherwise stated, or by passage through a 0.2 μm syringe filter. Chemically competent *E. coli* cells of DH10B and BL21(DE3) were used to make the cloning and protein production strains, respectively. Strains used in this work were stored in Luria-Bertani (LB) broth with a final concentration of 25% v/v glycerol at -80 °C. Microbial culturing was performed in New Brunswick Innova 44, at 37 ℃ overnight shaking for 200 rpm with a 2.5 cm orbit. If not mentioned in purpose, protein production was conducted at 16 ℃ for 20 hrs, induced by IPTG with a final concentration of 0.5 mM. Cells containing recombinant proteins were lysed using a Constant Systems Continuous Flow Cell Disruptor at 6 °C under 30 kpsi, with lysate passaging performed 3 times. Centrifugation was carried out using a Thermo Scientific IEX CL30R centrifuge with T41 swinging bucket rotor, or Beckman JXN-26 centrifuge with JS 5.3, JA 25.50, or JLA 8.100 rotors. Optical density at 600 nm (OD600) of *E. coli* cultures was measured by BMG LABTECH FLUOstar OMEGA microplate reader using clear flat-bottomed 96-well plates. Protein concentration was determined by measuring the absorbance at 280 nm, with a quartz LVis plate using microplate reader mentioned above. Plasmid pSG181 was derived from pET28a with an 8His tag and a TEV cleavage site placed at the N-terminus, which was constructed in-house. Plasmid constructs pSG181/CHEESY1 and pET28a/PrnF were also generated in-house. UPLC (Waters) was used to detect the new product formation after halogenation assays. Samples (5 μL) were injected into Waters Acquity BEH C18 1.7 μm 2.1 × 50 mm column set at 40 °C with a gradient wash from 90% solvent A (0.1% TFA water) and 10% solvent B (HPLC grade ACN) to 5% of solvent A and 95% of solvent B, at a flow rate of 0.6 mL/min. The elution was monitored by UV absorption (190nm-700nm).

LC-HRMS^2 with Thermo Orbitrap Velos Pro system was used for detection of product. Samples (5 μL) were injected into the Waters Xbridge C18 μm 2.1 × 100 mm column set at 40 °C. Analytes were eluted by 90% solvent A (0.1% formic acid water) and 10% solvent B

(HPLC grade ACN) to 5% of solvent A and 95% of solvent B, at a flow rate of 0.35 mL/min. UV absorption was monitored by a PDA detector at 220-800 nm (2 nm resolution, 10 Hz).

^{1}H NMR and ^{13}C NMR were recorded on Bruker Ascend 500 (500 MHz) NMR spectrometer. DMSO-d6, CD_3OD, or $CDCl_3$ were used as NMR solvents.

2.2 Docking Simulations of CHEESY1

CHEESY1 structure was predicted using AlphaFold v2[1]. CHEESY1 with FAD structure was predicted using AlphaFold v3[2]. The potential tunnel was predicted using CAVER Analyst v2.0[3] to recognise substrate binding site (BS). CHEESY1 hydrophobicity was studied with BioVia Discovery Studio v21.1.0.20298[4]. Prediction of the binding site position was carried out using AutoDockFR[5] and AutoSite v1.0[6]. Model validation was performed using MolProbity[7]. Structure figures were generated using PyMol Open Source V2.6.0a0[8] and substrate binding sites were mapped using LigPlot+ v.2.2.5[9]. Docking simulations were performed using AutoDockTools-1.5[10], employing AutoGrid4 for grid pre-calculations and AutoDock4 for docking simulations[11] using CHEESY1 chain as receptor with a grid size set to 62×52×84 points with 1.0 Å (volume: 270,816 Å^3) spacing centred on the receptor (x: 3,974; y: 1,968; z: 9,153). The Lamarckian genetic algorithm (LGA) with ten runs, population size of 150, maximum number of generations of 27,000 and a maximal number of energy evaluations of 25,000,000 was employed due its robustness and efficient performance[12]. Grid box containing catalytic lysine, K79 was recognised, and a map of the possible enzyme-substrate docking was created using AutoSite v1.0.

2.3 CHEESY1 Expression Vector Construction

The gene encoding CHEESY1 was synthesised by GeneArt Gene Synthesis (Thermofisher), and codon optimised for expression in *E. coli*. strain BL21(DE3). CHEESY1 was then cloned into the pET28a-derived vector using *Nde*I and *Hind*III digestion sites to construct the CHEESY1 expression vector. CHEESY1 expression vector was transformed into chemically competent *E. coli* (BL21-DE3) cells. Single colonies were picked and cultured at 37 °C, 200 rpm with a 2.5 cm orbit, overnight.

2.4 Production and Purification of CHEESY1 and PrnF

An overnight starter culture was diluted 1:100 into LB medium (500 mL) supplemented with 50 µg/mL kanamycin. Cultures were incubated at 37 °C, 200 rpm (2.5 cm orbit) until OD_{600} reached 0.4-0.6. Cultures were then cooled to 16 °C and induced by IPTG at a final concentration of 0.5 mM. Cells were harvested by centrifugation (6 °C, 10 min, 4000 ×*g*). Cell pellets were resuspended in lysis buffer (50 mM sodium phosphate, 250 mM NaCl, pH 7.8) and lysed using a cell disruptor at 6 °C under 30 kpsi for 3 runs. The crude lysate was clarified by centrifugation at 20,000 ×*g* at 6 °C for 50 min (Avanti JXN-26, Beckman Coulter, rotor JA 25.5). Proteins were purified by Ni-NTA column using HisPur Ni-NTA resin (ThermoFisher), eluted with 250 mM imidazole. The purification process was monitored qualitatively using the Bradford reagent (Bio-Rad). Imidazole was removed by overnight dialysis at 4 °C in protein storage buffer (50 mM HEPES, 10% glycerol v/v, pH 8.0). Dialysed proteins were concentrated using Amicon Ultra-15 Centrifugal Filter units at 4000 ×*g* at 4 °C. Concentrated proteins were filtered using a 0.22 µM syringe membrane (MF-Millipore™), then purified by size exclusion

chromatography (SEC) using a HiLoad 16/600 Superdex 200 column (GE Healthcare Life Science) at 1 mL/min flowrate using protein storage buffer as the running buffer. Elution fractions from SEC were analysed by SDS-PAGE.

The production and purification of PrnF followed the same procedure as described above. PrnF expression vector was derived from pET21a, constructed in-house, with 6xHistag at the C-terminal.

2.5 Protein Identification by SDS-PAGE and MS

Protein solution sample from the elution fractions (5 µL) was mixed with an equal volume of 2× LDS loading buffer (prediluted from the original 4× LDS loading buffer) and heated at 95 °C for 10 minutes. 5 µL of heated sample was loaded in the precast protein gel (NuPAGE, ThermoFisher) and was ran at 200V, 180 mA for 30 min. The protein gel was stained using Instant Blue (Expedeon) at RT for 1 h. A volume of 5 µL of a 10-200 kDa unstained protein ladder (NEB) was used to monitor the size of protein bands. The band of the target protein was excised from the gel and sent to MALDI MS & MSMS (Mass spectrometry and proteomics facility, University of St Andrews) to confirm the identity of the protein. Elution fractions showing pure protein, were combined and concentrated. Protein concentration was measured under 280 nm using the plate reader (BMG Labtech FLUOstar Omega). Proteins were finally divided into small aliquots (100 µL-1 mL for each aliquot), frozen by liquid nitrogen and stored at -80 °C until use.

2.6 Small-Scale Halogenation Assays

Small-scale halogenation assays (100 µL) were conducted in V-bottom 96 well plates (Griener Bio-One) using 50 mM HEPES, pH 8.0 as the reaction buffer. Frozen reagents were thawed on ice before use. All the reagents were added into the wells in turn, with the final concentrations of reagents as follows: 50 µM CHEESY1, 1 µM PrnF, 10 µM FAD, 2.5 mM NADH, 10 mM NaCl/NaBr, 500 µM substrate, 200 U/mL catalase and 10 U/mL SOD. The plate was covered by a gas-permeable film and was incubated at 30 °C, 180 rpm (orbit 2.5 cm) overnight. Reactions were quenched with 100 µL HPLC grade MeOH and centrifuged at 6000 ×*g* for 20 min. 100 µL of clarified supernatant of each reaction was carefully transferred to a new V-bottom 96 well plate for detection.

2.7 Monochlorodimedone (MCD) assays

Each assay was performed in triplicate. MCD exhibits a λmax at 270 nm. The peak area of MCD in each sample was measured at 270 nm. The final concentration of MCD in all assays was 1 mM. Reaction conditions of Assay 1, Assay 2, and Assay 3 were listed in Table S1. Assay 1: MCD + standard halogenation components excluding substrate. Assay 2: MCD + standard halogenation components including substrate. Assay 3: MCD + all components except CHEESY1 and substrate. Reactions (100 µL final volume) were performed in 96-well plates as described in SI section 2.6.

Table S1. Reaction components of MCD Assay1, Assay 2 and Assay 3.

	Concentration	ASSAY1	ASSAY2	ASSAY3
CHEESY1	50 µM	√	√	**X**
FAD	10 µM	√	√	√
NADH	2.5 mM	√	√	√
PrnF	1 µM	√	√	√
NaBr/NaCl	10 mM	√	√	√
SUBSTRATE	500 µM	**X**	√	**X**
CATALASE	200 U/mL	√	√	√
SOD	10 U/mL	√	√	√
**MCD**	1 mM	√	√	√
HEPES buffer (pH 8.0)	50 mM	√	√	√

2.8 Chemical Bromination Using N-Bromosuccinimide (NBS) or Sodium Hypobromide (NaOBr) against All Substrates

Reactions were performed in 100 µL volumes with a substrate concentration of 1 mM and brominating agents (NBS or NaOBr) of 1 mM or 5 mM (i.e., 1 equiv or 5 equiv). The reactions were carried out in a 96-well plate and incubated at room temperature for 3 hours, then quenched with 100 µL of HPLC-grade MeOH and centrifuged at 6000 ×*g* for 20 min. After centrifugation, 100 µL of the clarified supernatant from each reaction was transferred to a new V-bottom 96-well plate for UPLC analysis. UPLC conversions were measured at specific wavelengths to optimise peak detection: 254 nm for substrates **1** and **4**, 245 nm for **2**, and 300 nm for **3**, **5**, **6**, and **7**. UPLC conversion (%) represents the % of product peak area at the specified wavelength.

2.9 Large Scale Halogenation Assays

The final concentrations of reagents for scale-up was as follows: 30-50 µM CHEESY1, 2 µM PrnF, 10 µM FAD, 500 µM NADH, 10 mM NaCl/NaBr, 1-3 mM substrate, 200 U/mL catalase, 10 U/mL SOD, 1% IPA, and 20 U/mL ADH. 20 mM sodium phosphate, pH 8.0 was used as the reaction buffer. A total of 10 mg substrate was added into the reaction. Glass vials containing reaction mixtures were sealed with gas permeable film and incubated at 30 °C, 180 rpm (orbit 2.5 cm) for 2-3 days until no more conversion was observed on UPLC.

2.10 Kinetics

All measurements were conducted in triplicate. The conditions for measuring the kinetics of the reaction were as follows: 25 μM CHEESY1, 1 μM PrnF, 10 μM FAD, 2.5 mM NADH, 10 mM NaCl/NaBr, 10 μM-10 mM substrate, 200 U/mL catalase and 10 U/mL SOD, with 50 mM HEPES buffer, pH 8.0, in a total volume of 100 μL. Reactions were conducted at 30 °C, 180 rpm (orbit 2.5 cm). Reactions were quenched at 6, 12, 18, and 24 min by adding 100 μL HPLC-grade MeOH and cooled on ice. Quenched reactions were centrifuged (6000 ×*g* for 20 min) and analysed by UPLC. Product formation was calculated based on the product standard curve. A range of time points and corresponding rate of product formation were fitted to generate Michaelis-Menten equation by Prism 9.4.0 (GraphPad Software, USA).

2.11 Product Purification and Identification

An equal volume of ethyl acetate was added into reaction solution for quenching and extraction purpose. The extracted mixture was vortexed and centrifuged at 4000 ×*g* for 5 min. Supernatant was transferred to a clean round bottom flask. This extraction procedure was repeated 3-4 times until no product was seen in the reaction solution (as monitored by UPLC). Extract was dried by Genevac (SP Genevac EZ-2, Sysmex Belgium N.V.) under Medium+Low BP mode at 40 ℃, then purified using a Biotage Isolera 4 by normal phase chromatography using a silica column (10 g) (100 % Hexane to 100 % DCM 20-30 column volumes as required), or reverse phase chromatography (Biotage® Sfär C18 D - Duo 100 Å 30 μm, gradient from pure H_2O to 100% MeOH, 30 column volumes). The fractions containing product were combined and dried by Genevac under HPLC mode. Dried product was dissolved in appropriate deuterated solvents (Chloroform-d or Methanol-d4) and detected by ^{1}H NMR and ^{13}C NMR (conditions were shown in NMR spectrums).

2.12 Thermal Shift Assay

Thermal shift assays were conducted in triplicate at a volume of 25 μL for each assay. Assays were set up using different protein concentrations (2,4,6 and 8 μM) and Sypro Orange protein dye (1x, 2x, 3x and 4x concentrations) in 50 mM HEPES pH 8.0 as the test buffer. A real-time PCR machine (Stratagene Mx3005P with software MxPro v 4.01) was used to scan the fluorescence signals from denatured proteins over a temperature range of 25 °C to 95 °C, ramping at 0.5 °C per min.

2.13 Thermal Stability Assay of CHEESY1 with Th-Fre and PrnF

Small-scale halogenation assays were conducted for CHEESY1 thermal stability test. Assay conditions were followed as described in SI-2.6. Th-Fre (1 μM) was used as the flavin reductase to test CHEESY1 thermal stability, with PrnF with CHEESY1 as a positive control.

2.14 Site-Directed Mutagenesis

Mutagenesis was performed according to the protocol by Liu *et al* (2008) [13]. The primers designed for generating mutation variants are listed in Table S2, and the PCR procedure was carried out as described in Table S3.

Table S2. Primers used for site-directed mutagenesis of CHEESY1 WT to generate Y170I, Y170V, S311I, S311V, and S311A.

Y170I	Forward Primer	GCGGTATTGGTCGTGT TCTGGCACGTCTGGAAGC
	Reverse Primer	ACACGACCAATACCGC TTGCATCCAGAACAAAACGTGC
Y170V	Forward Primer	GCGGTGTTGGTCGTGT TCTGGCACGTCTGGAAGC
	Reverse Primer	ACACGACCAACACCGC TTGCATCCAGAACAAAACGTGC
S311I	Forward Primer	CCGGTTTTTATCAGCG GTGTTACCATTGCACTGCATAGCG
	Reverse Primer	CGCTGATAAAAACCGG ATCCAGAAATTCACCGGCATTACC
S311V	Forward Primer	CCGGTTTTTGTCAGCG GTGTTACCATTGCACTGCATAGCG
	Reverse Primer	CGCTGACAAAAACCGG ATCCAGAAATTCACCGGCATTACC
S311A	Forward Primer	GGTTTTTAGCAGCGCTGTTACCATTGCACTGCATAGCGC
	Reverse Primer	CAGCGCTGCTAAAAACCGGATCCAGAAATTCACCGGCATTAC

Table S3. PCR cycles for site-directed mutagenesis.

<table>
<tr><th rowspan="2">Components</th><th rowspan="2">Concentration</th><th colspan="3">Procedure</th></tr>
<tr><th>Ram 1</th><th>Ram 2</th><th>Template digestion</th></tr>
<tr><td>5 × Phusion HF buffer</td><td>1x</td><td rowspan="5">1. 98 °C for 30 s
2. 98 °C for 10 s
3. app. annealing T, 30 s
4. 72 °C for 3min 30 s
5. Repeat 2-4 for 12 cycles</td><td rowspan="5">6. 98 °C for 30 s
7. 98 °C for 10 s
8. app. annealing T, 30 s
9. 72 °C for 3min 30 s
Repeat 2-4 for 12 cycles</td><td rowspan="5">Add 1 µL DpnI, incubate at 37 °C for 1 h</td></tr>
<tr><td>10 mM dNTPs</td><td>200 µM</td></tr>
<tr><td>Primers</td><td>0.5 µM</td></tr>
<tr><td>Template DNA</td><td>/</td></tr>
<tr><td>Phusion DNA polymerase</td><td>0.5 µL</td></tr>
</table>

Nuclease-free water	up to 50 μL			

3 Supporting Results

3.1 Sequence Alignment Results of CHEESY1

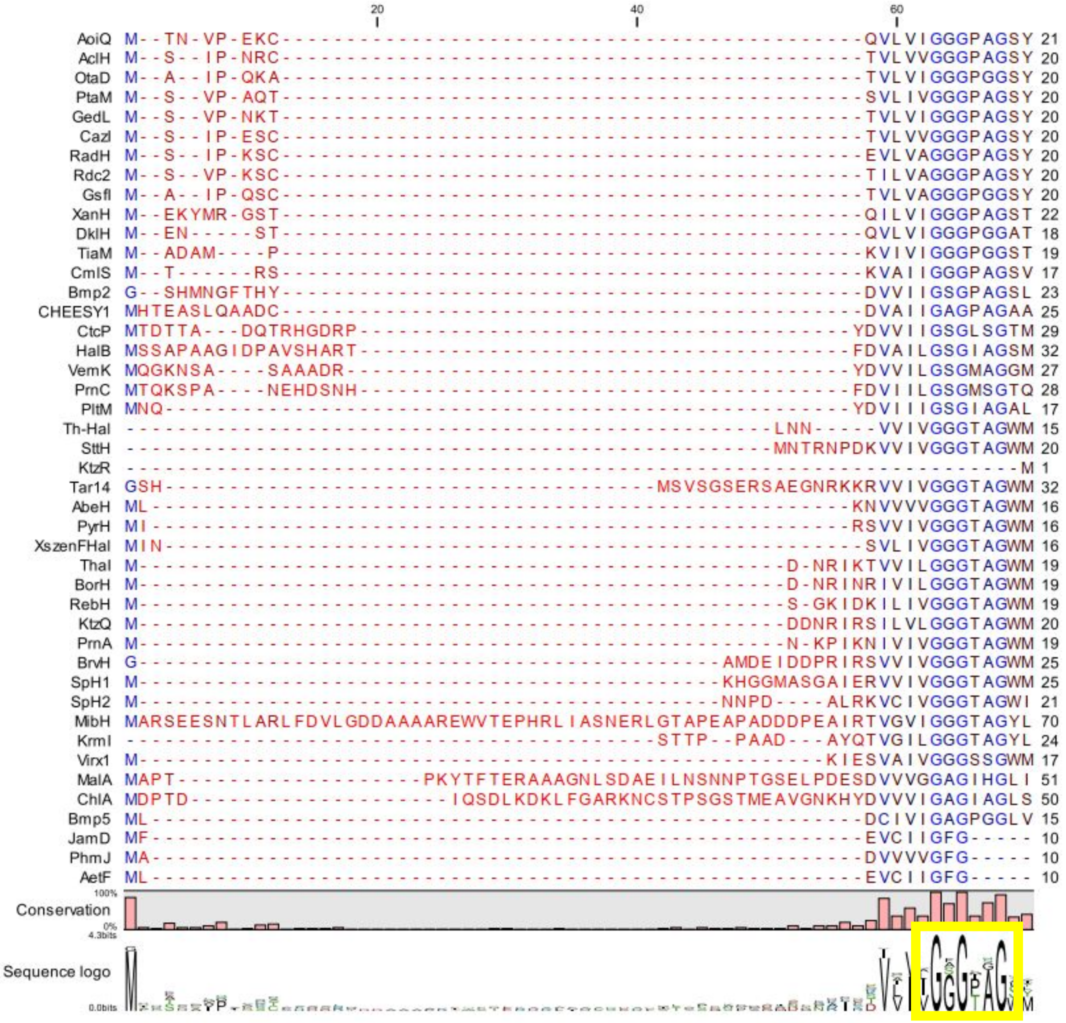

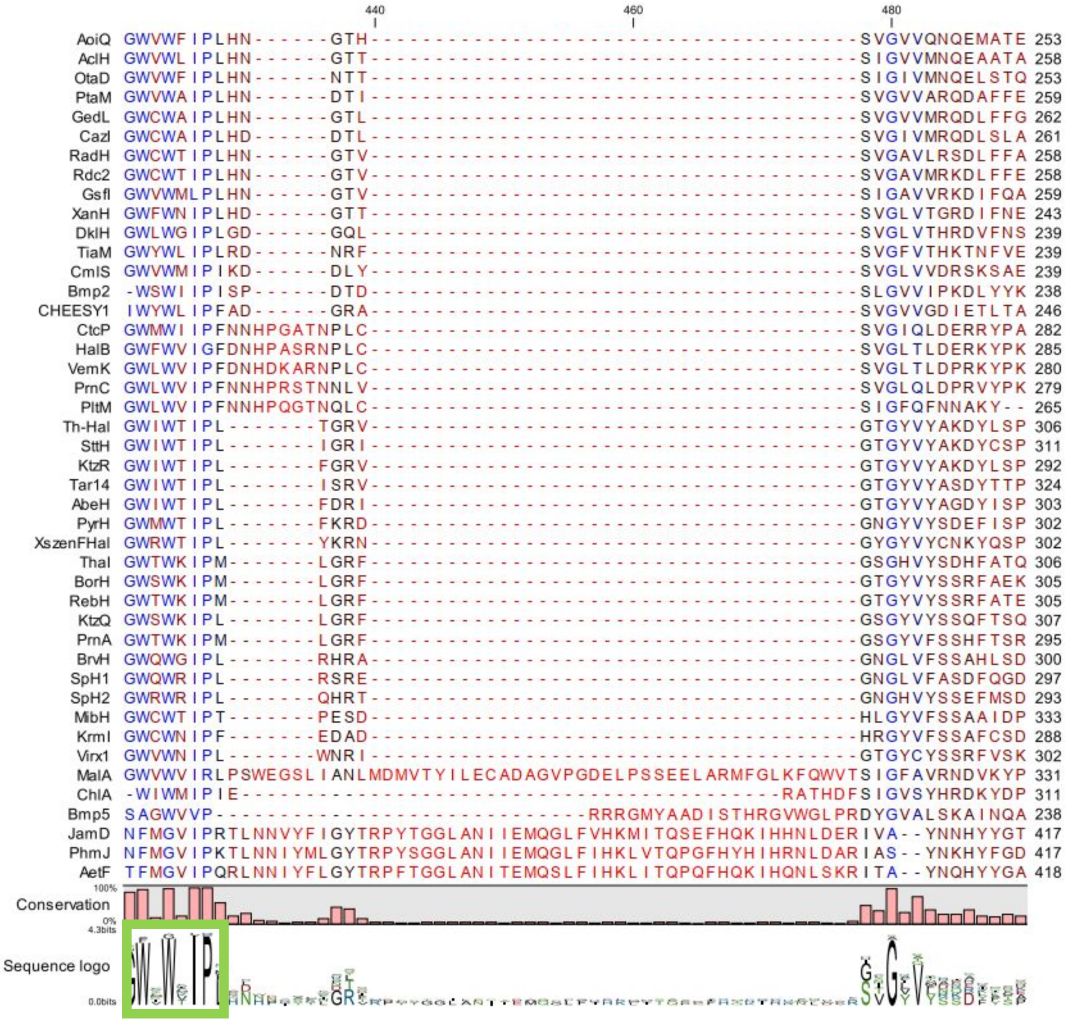

440 460 480
AoiQ GWVWFIPLHN------GTH--------------------------------------SVGVVQNQEMATE 253
AclH GWVWLIPLHN------GTT--------------------------------------SIGVVMNQEAATA 258
OtaD GWVWFIPLHN------NTT--------------------------------------SIGIVMNQELSTQ 253
PtaM GWVWAIPLHN------DTI--------------------------------------SVGVVARQDAFFE 259
GedL GWCWAIPLHN------GTL--------------------------------------SVGVVMRQDLFFG 262
Cazl GWCWAIPLHD------DTL--------------------------------------SVGIVMRQDLSLA 261
RadH GWCWTIPLHN------GTV--------------------------------------SVGAVLRSDLFFA 258
Rdc2 GWCWTIPLHN------GTV--------------------------------------SVGAVMRKDLFFE 258
Gsfl GWVWMLPLHN------GTV--------------------------------------SIGAVVRKDIFQA 259
XanH GWFWNIPLHD------GTT--------------------------------------SVGLVTGRDIFNE 243
DklH GWLWGIPLGD------GQL--------------------------------------SVGLVTHRDVFNS 239
TiaM GWYWLIPLRD------NRF--------------------------------------SVGFVTHKTNFVE 239
CmlS GWVWMIPIKD------DLY--------------------------------------SVGLVVDRSKSAE 239
Bmp2 -WSWIIPISP------DTD--------------------------------------SLGVVIPKDLYYK 238
CHEESY1 IWYWLIPFAD------GRA--------------------------------------SVGVVGDIETLTA 246
CtcP GWMWIIPFNNHPGATNPLC--------------------------------------SVGIQLDERRYPA 282
HalB GWFWVIGFDNHPASRNPLC--------------------------------------SVGLTLDERKYPK 285
VemK GWLWVIPFDNHDKARNPLC--------------------------------------SVGLTLDPRKYPK 280
PrnC GWLWVIPFNNHPRSTNNLV--------------------------------------SVGLQLDPRVYPK 279
PltM GWLWVIPFNNHPQGTNQLC--------------------------------------SIGFQFNNAKY-- 265
Th-Hal GWIWTIPL-------TGRV--------------------------------------GTGYVYAKDYLSP 306
SttH GWIWTIPL-------IGRI--------------------------------------GTGYVYAKDYCSP 311
KtzR GWIWTIPL-------FGRV--------------------------------------GTGYVYAKDYLSP 292
Tar14 GWIWTIPL-------ISRV--------------------------------------GTGYVYASDYTTP 324
AbeH GWIWTIPL-------FDRI--------------------------------------GTGYVYAGDYISP 303
PyrH GWMWTIPL-------FKRD--------------------------------------GNGYVYSDEFISP 302
XszenFHal GWRWTIPL-------YKRN--------------------------------------GYGYVYCNKYQSP 302
Thal GWTWKIPM-------LGRF--------------------------------------GSGHVYSDHFATQ 306
BorH GWSWKIPM-------LGRF--------------------------------------GTGYVYSSRFAEK 305
RebH GWTWKIPM-------LGRF--------------------------------------GTGYVYSSRFATE 305
KtzQ GWSWKIPL-------LGRF--------------------------------------GSGYVYSSQFTSQ 307
PrnA GWTWKIPM-------LGRF--------------------------------------GSGYVFSSHFTSR 295
BrvH GWQWGIPL-------RHRA--------------------------------------GNGLVFSSAHLSD 300
SpH1 GWQWRIPL-------RSRE--------------------------------------GNGLVFASDFQGD 297
SpH2 GWRWRIPL-------QHRT--------------------------------------GNGHVYSSEFMSD 293
MibH GWCWTIPT-------PESD--------------------------------------HLGYVFSSAAIDP 333
KrmI GWCWNIPF-------EDAD--------------------------------------HRGYVFSSAFCSD 288
Virx1 GWVWNIPL-------WNRI--------------------------------------GTGYCYSSRFVSK 302
MalA GWVWVIRLPSWEGSLIANLMDMVTYILECADAGVPGDELPSSEELARMFGLKFQWVTSIGFAVRNDVKYP 331
ChlA -WIWMIPIE--RATHDFSIGVSYHRDKYDP 311
Bmp5 SAGWVVP-----------------------------RRRGMYAADISTHRGVWGLPRDYGVALSKAINQA 238
JamD NFMGVIPRTLNNVYFIGYTRPYTGGLANIIEMQGLFVHKMITQSEFHQKIHHNLDERIVA--YNNHYYGT 417
PhmJ NFMGVIPKTLNNIYMLGYTRPYSGGLANIIEMQGLFIHKLVTQPGFHYHIHRNLDARIAS--YNKHYFGD 417
AetF TFMGVIPQRLNNIYFLGYTRPFTGGLANITEMQSLFIHKLITQPQFHQKIHQNLSKRITA--YNQHYYGA 418
100%
Conservation
0%
4.3bits
Sequence logo
0.0bits

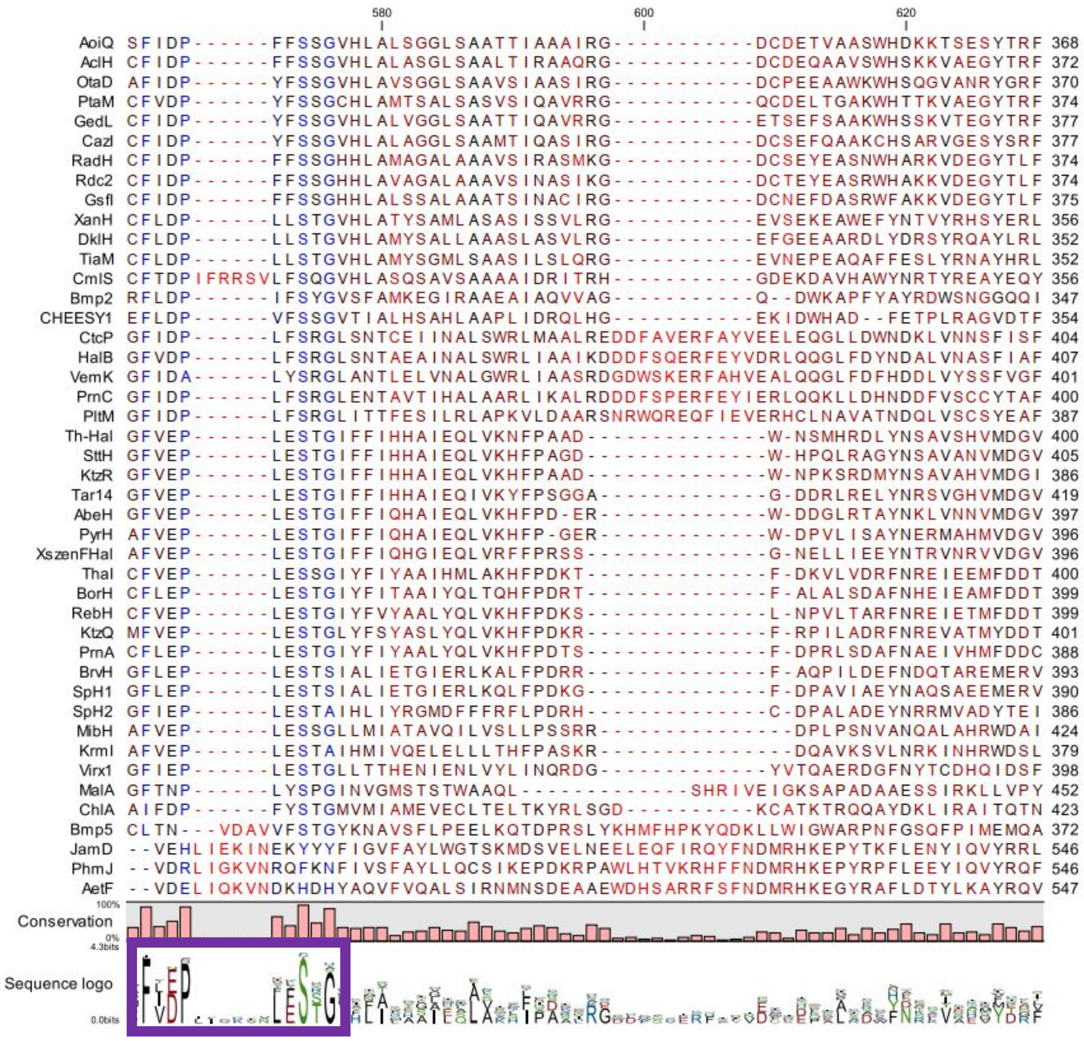

Figure S1. Sequence alignment of CHEESY1 with known FDHs. Only segments containing motifs are shown here. Flavin binding motif GxGxxG is shown in a yellow box, WxWxIP motif is shown in a green box and the Fx.Px.Sx.G motif, shown in a purple box.

3.2 Docking Simulations of CHEESY1 with Selected Substrates

3.2.1 Procedure for Substrate Binding Site Determination

First, by visualization of the 3D structure of CHEESY1 with catalytic lysine highlighted, we found that there were four possible locations of the substrate binding sites (BS) with approximate distances from the Nitrogen of the catalytic residue K79: pocket 1 (P1) was about 4Å, pocket 2 (P2) was 7Å, pocket 3 (P3) was 10Å, and pocket 4 (P4) was 13 Å (Figure S2 A). Pockets P3 & P4 were far enough away to be ignored. P1 & P2 had high hydrophobicity, while FAD groups located in the hydrophilic area (Figure S2B).

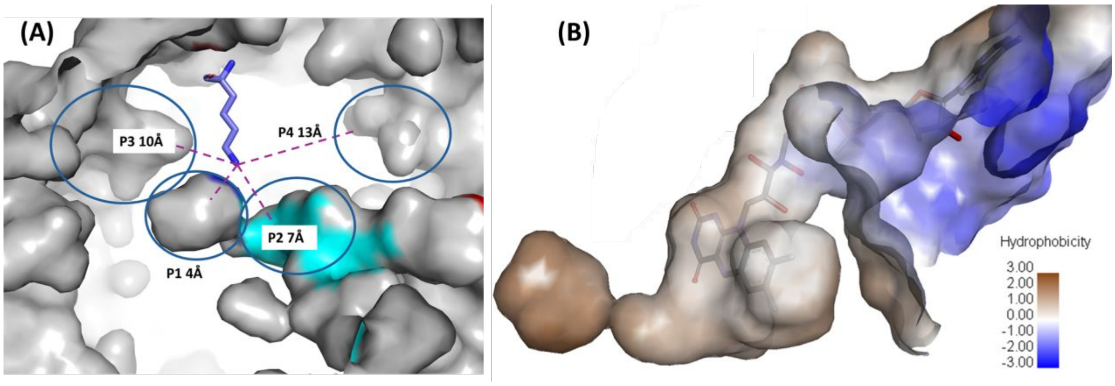

Figure S2. Surface organization of CHEESY1. (A) Surface representation of CHEESY1 pockets. CHEESY1 – grey coloured, catalytic lysine K79 – blue stick, FAD binding site (BS): 1st motif GxGxxG – red; 2nd motif WxWxIP – magenta; 3rd motif Fx.Px.SxG – cyan, Putative Substrate BSs – P1, P2, P3, P4 – blue circles. (B) CHEESY1 showing substrate BS and hydrophobicity with FAD bound and putative substrate BS hydrophobicity. Highly hydrophobic area – brown, highly hydrophilic area – blue (see scale).

To better recognize and understand the substrate BS, we explored the potential tunnel. The tunnel was 10.9 Å length, 2.79Å maximal radius and did not reach the P1 site (Figure S3). The tunnel-forming residues were R47, S49, I50, G51, E52, S53, L54, K79, N301, G169, Y170, S311, S312, G313, V314. Residues S53, L54, S311, S312 were involved in both P1 and P2 surface forming.

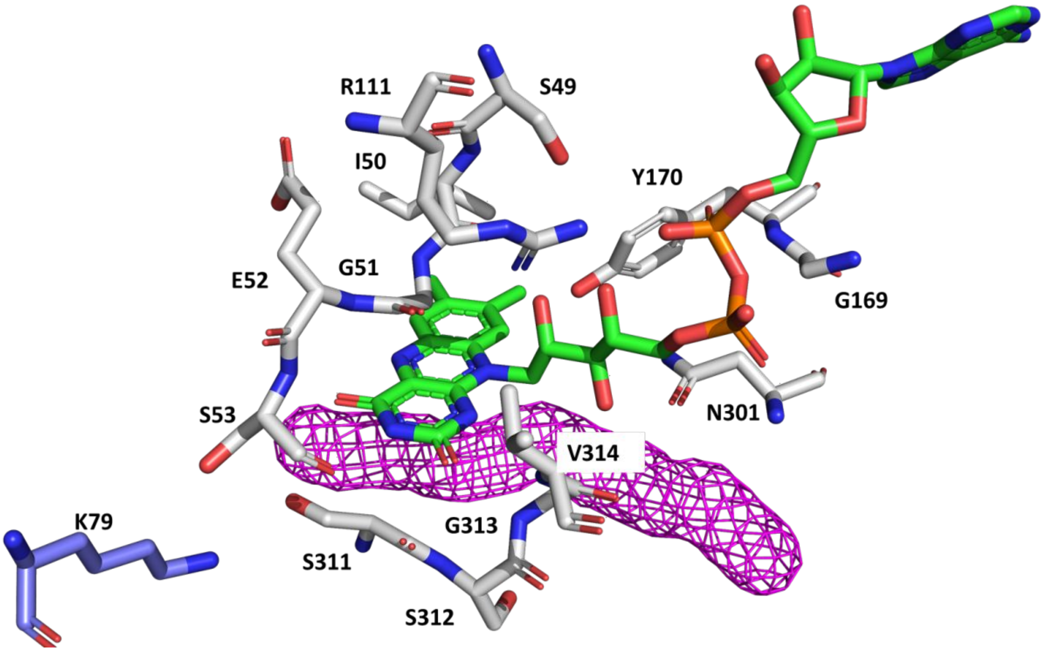

Figure S3. Modelled tunnel (magenta mesh) with FAD (green), tunnel-forming residues (grey), & K79 (blue).

Next, using rigid docking to determine possible binding position, we selected boxes encompassing P1 & P2, and tested with a group of aromatic compounds all containing Nitrogen and/or Oxygen (Figure S4). We collected two scores, which were binding energy and distance

from catalytic lysine residue. By comparison of these scores, we could make a conclusion about a more probable BS of these different substrates (Table S4).

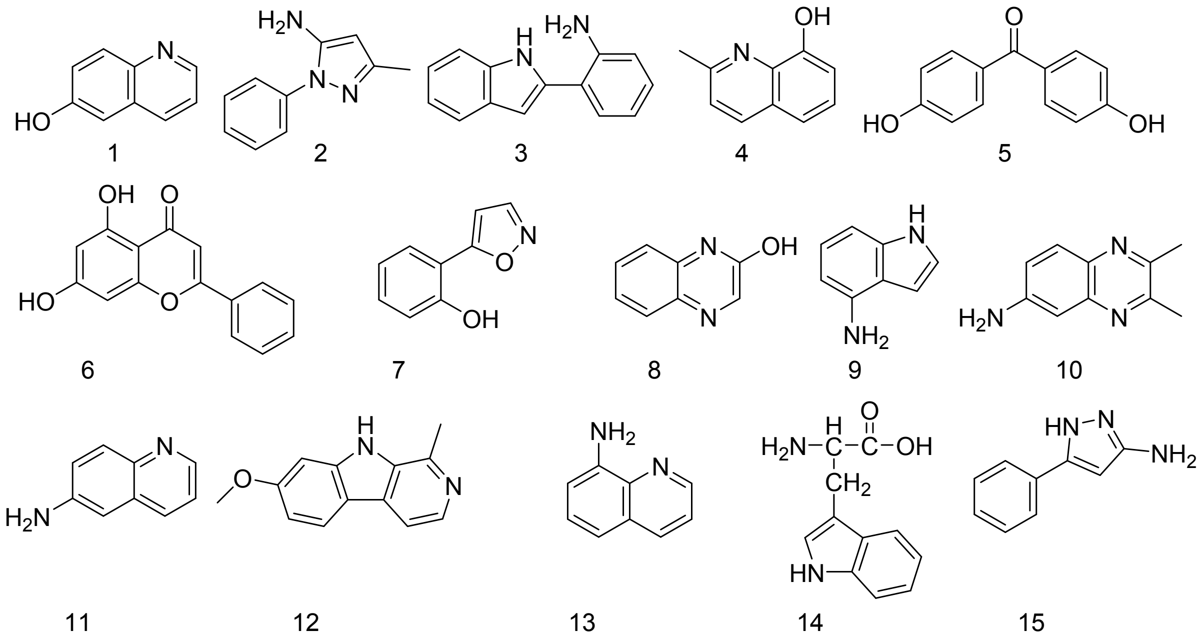

Figure S4. List of compounds for quick molecular docking test.

Table S4. Comparation of the pockets P1 & P2 with scores: distance from K79 and binding energy.

Compound	Rigid docking						Flexible docking		
	Pocket P	Distance from K79 (Å)	Binding energy (kJ/mol)	Pocket P	Distance from K79 (Å)	Binding energy (kJ/mol)	Pocket P	Distance from K79 (Å)	Binding energy (kJ/mol)
1		4.9	-4.6		7.7	-26.4		4.9	-6.3
2		3.7	-4.2		9.0	-25.9		4.5	+8.0
3		4.4	-8.8		8.3	-30.1		3.7	+8.8
4		3.5	+7.1		8.0	-22.5		3.5	+3.8
5		4.1	-8.8		7.5	-29.3		3.8	-13.4
6		4.3	+10.0		7.9	-36.0		3.9	+11.3
7		4.3	-13.4		7.5	-24.3		3.7	+6.3
8	1	3.5	-2.1	2	7.1	-24.7	1	3.5	-4.6
9		4.6	-6.7		8.7	-23.9		4.6	-6.3
10		3.9	-0.4		9.0	-25.9		5.4	-1.3
11		3.5	-0.8		7.8	-22.5		4.1	-0.4
12		3.5	+9.2		7.1	-25.9		3.9	+18.0
13		3.9	-6.3		7.6	-26.4		3.5	+1.7
14		3.4	-0.0		10.6	-27.2		3.5	+5.9
15		4.1	+15.1		12.4	-29.3		4.0	+8.4

To understand whether P1 could be a substrate binding site, we proved the flexible docking with pocket-forming residues S53, L54, P56, F84, K79, F95, S311, S312, for a group of compounds (Figure S4). Although flexible docking had shown that even bulky compounds could fit into P1 (Figure S5), positive binding energy indicated that the substrates would not be accepted/bound within this pocket (Table S4).

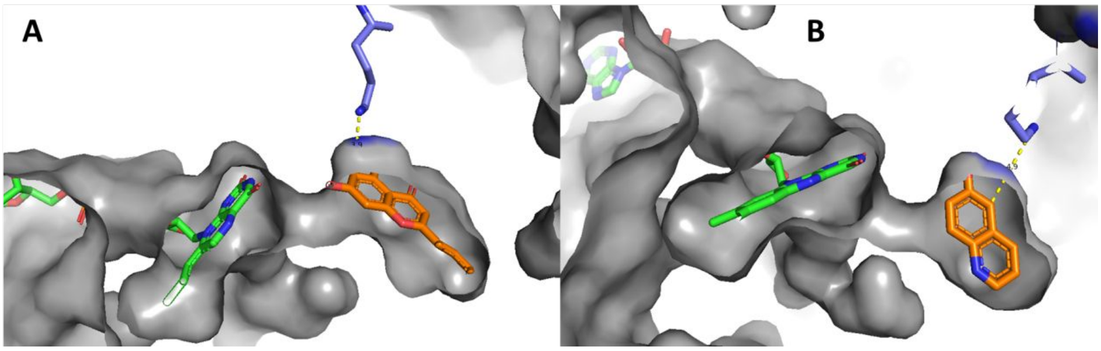

Figure S5. Positions substrate in the pocket P1. (A) chrysin (6), (B) 6-hydroxyquinoline (1). CHEESY1 – grey coloured, catalytic Lysine K 79 – blue stick, FAD molecule – green, substrate molecules – orange.

In any case, P1 showed quite high binding energies, which indicated unlikely formation of the ligand-protein complexes. Thus, the only possibility of substrate binding remained in P2.

By analysis of FDH crystal structures, we identified a number of FDHs with similar surface organization to that of P2, these being RadH, PltM, Myp16, Bmp2, Cmls, ChB4, and FasV (PDB code: 8GU0, 5DBJ, 5BUK, 5BVA, 3I3L, 7FCO, 8ZOP respectively). If we consider that for structures Bmp2, ChlB4, and FasV, the substrate BS is located close to the outer surface interface, we could assume the possibility of conformational changes in the structure of the active site. However, for the group of RadH, PltM, Myp16, and CmlS, such an assumption is extremely unlikely or would require dramatical conformational change. In work by Menon *et al* [14] and Peh *et al* [15], a similar problem was observed when studying RadH. The authors docked 6-hydroxyquinoline (substrate **1**) using DiffDock and suggested that the active site of RadH was the space currently occupied by the flavin moiety of FAD, and this caused overlap with the FAD structure [15]. Although RadH did not have high identity with CHEESY1(46 % / 27 %, similarity / identity), analysis of the surface of the crystal structure and molecular docking[14, 15] revealed a certain similarity, which is quite unique for the group of phenolic two-component flavin-dependent halogenases (Figure S6).

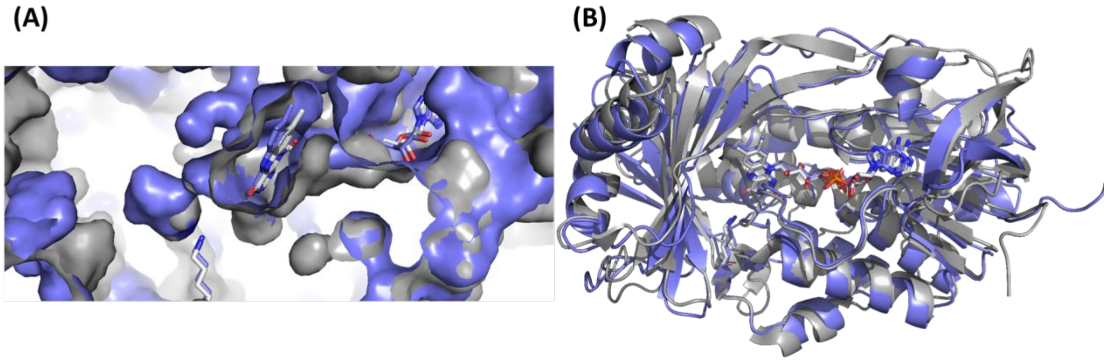

Figure S6. CHEESY1 AlphaFold with FAD structure – blue, RadH crystal structure with FAD (PDB code 8gu0) – grey, (A) surface of the proteins, (B) cartoon of the proteins

Next, by analysis of the substrate-protein interaction using rigid docking with P2, we reduced the list of pocket-forming residues by selecting more common residues, followed by, more targeted docking with the next compounds (Table S5).

Table S5. Flexible docking prediction for Pocket 2 (see Figure S2 A).

Compound		Distance from K79 (Å)	Binding energy (kJ/mol)	Compound		Distance from K79 (Å)	Binding energy (kJ/mol)
No	Formula			No	Formula		
1	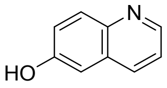	8.6	-21.8	28	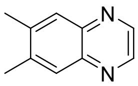	7.8	-24.3
2	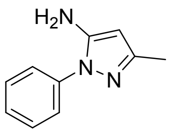	9.5	-31.8	29	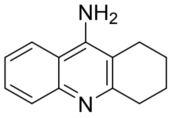	10.0	-29.3
3	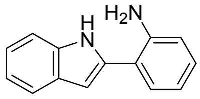	9.8	-24.7	30	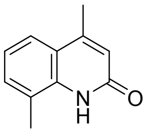	10.0	-28.0
4	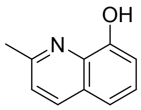	8.8	-26.0	31	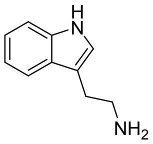	7.6	-16.0
5	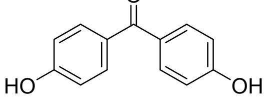	7.8	-31.9	32	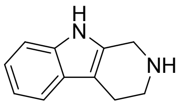	7.5	-28.5

6	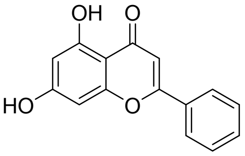	7.8	-36.0	33	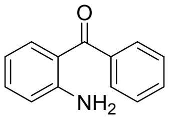	8.8	-29.3
7	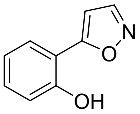	8.3	-24.7	34	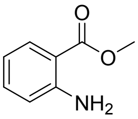	6.8	-17.6
8	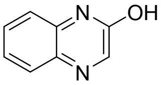	10.4	-22.5	35	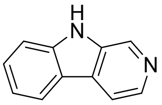	9.5	-20.8
9	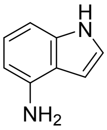	7.6	-19.3	36	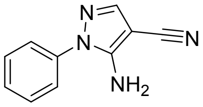	13.4	-29.7
10	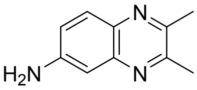	7.8	-18.8	37	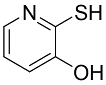	8.1	-18.8
11	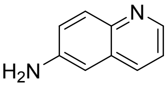	7.5	-15.9	38	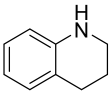	8.4	-19.7
12	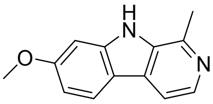	8.1	-20.1	39	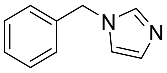	8.2	-17.6
13	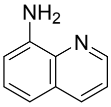	7.1	-12.6	40	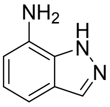	8.3	-20.8
14	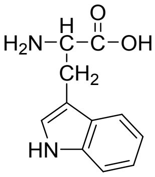	11.4	-31.8	41	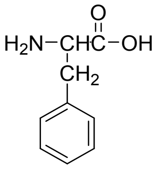	7.8	-23.4
15	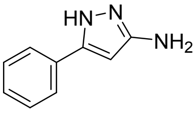	7.8	-18.8	42	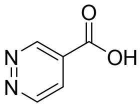	8.5	-20.5
16	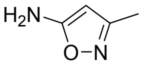	8.9	-18.0	43	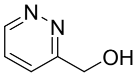	8.3	-20.5
17	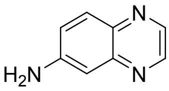	10.5	-25.1	44	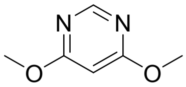	8.6	-19.3
18	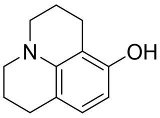	11.7	-31.4	45	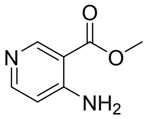	7.8	-15.9

19	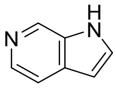			46	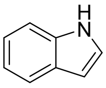	9.5	-20.5
20	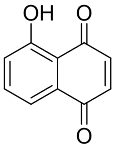	7.9	-25.5	47	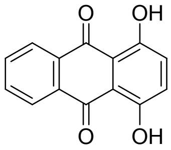	11.5	-30.5
21	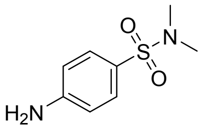	10.8	-22.6	48	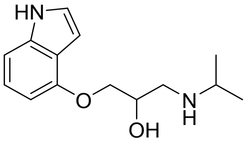	7.5	-22.5
22	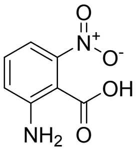	8.8	-13.4	49	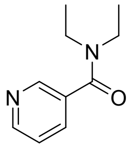	7.3	-10.9
23	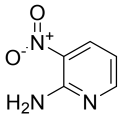	7.7	-18.0	50	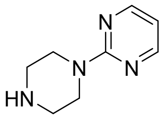	7.1	-17.6
24	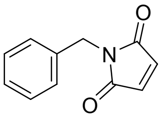	10.5	-22.5	51	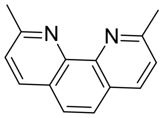	7.3	-20.8
25	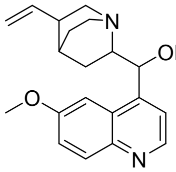	7.9	-15.5	52	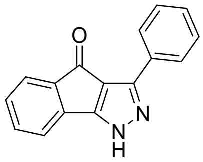	7.2	-33.9
26	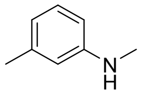	7.1	-25.9	53	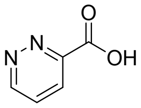	8.4	-20.8
27	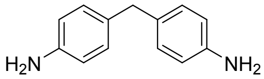	8.2	-21.8				

CELL – orange-coloured cells are not correctly predicted (False Positive prediction, also known as Type I Error or False Alarm, 9)

CELL – green coloured cells are true positive predicted (True Positive prediction, 7)

CELL – pink coloured cells are true negative predicted (True Negative prediction, 37)

Finaly, by selection scores, which were binding energies (BE) (-20.9 >BE> -41.8 kJ/mol) and distances from catalytic residues (<10 Å) with regioselective analysis (Table S5), we applied compounds for protein assay (total number of compounds is 53), 37 from them were not transformed by CHEESY1 but 16 were. Wet validation mostly confirmed our suggestion: from 16 positive predicted, 9 were wrong predicted as positive (Indicated by orange colour in Table S5). Overall, the accuracy of our model was 83.02%.

Based on these results, we can cautiously assume the putative substrate active site. In any case, we could claim that the docking model we developed was successful in assessing the possibility of biotransformation of substrates and their possible regioselectivity.

3.2.2 Application of Substrate Molecular Docking to Selected Substrates

The legend of Figure S7 provides a comprehensive explanation and illustration of the docking result. For subsequent figures, similar explanations have been simplified and the variations specific to each figure have been maintained.

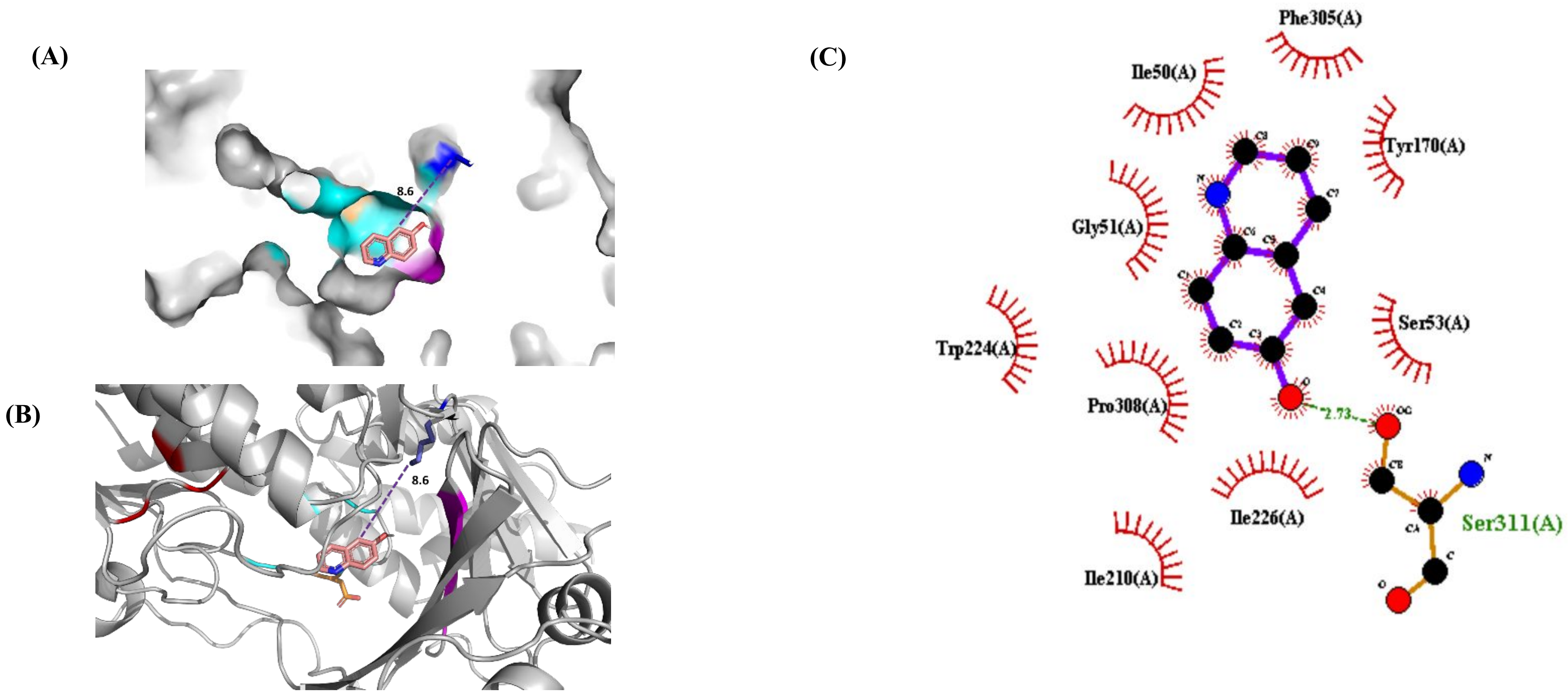

Figure S7. Structural overview of CHEESY1 substrate binding site with bound compound **1** from flexible docking. (A) Surface representation of CHEESY1 with bound compounds (stick style) in binding cleft. (B) Close up of CHEESY1 binding site, binding residues and compounds were depicted as sticks. For (A)-(B), CHEESY1 surface style, cartoon style and carbons were coloured in grey, K79 was represented as a blue stick, D307 in orange, second motif WxWxIP in magenta, third motif Fx.Px.Sx.G in cyan. Compounds were coloured in pink (carbons). Nitrogen, oxygen and hydrogen atoms were coloured in blue, red and white, respectively. (C) LigPlot+ v.2.2.5 derived representation of CHEESY1 substrate binding site. Dashed half circles represent hydrophobic contacts and dashed lines hydrogen bonds. Carbons, nitrogen and oxygen atoms were coloured in black, blue, and red, respectively.

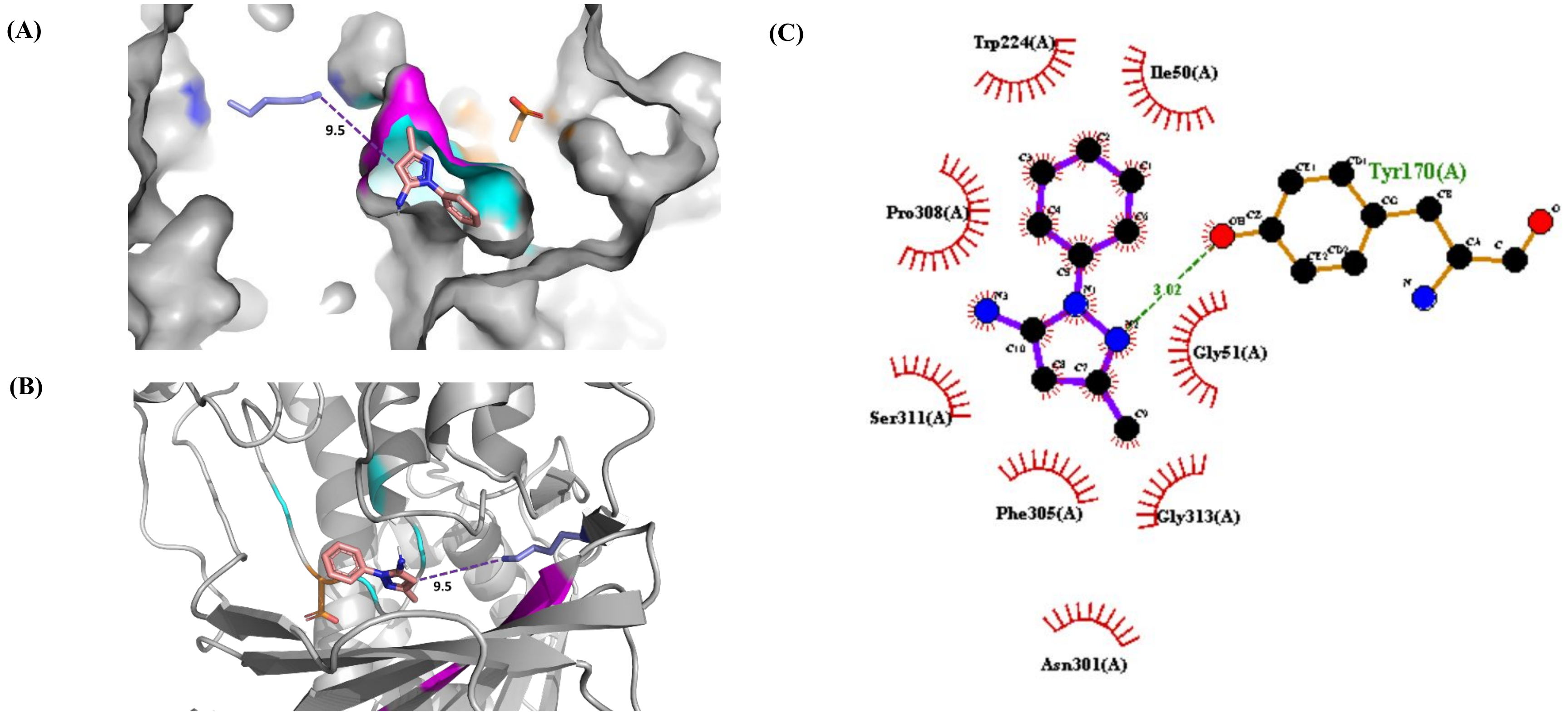

Figure S8. Structural overview of CHEESY1 substrate binding site with bound compound **2**.

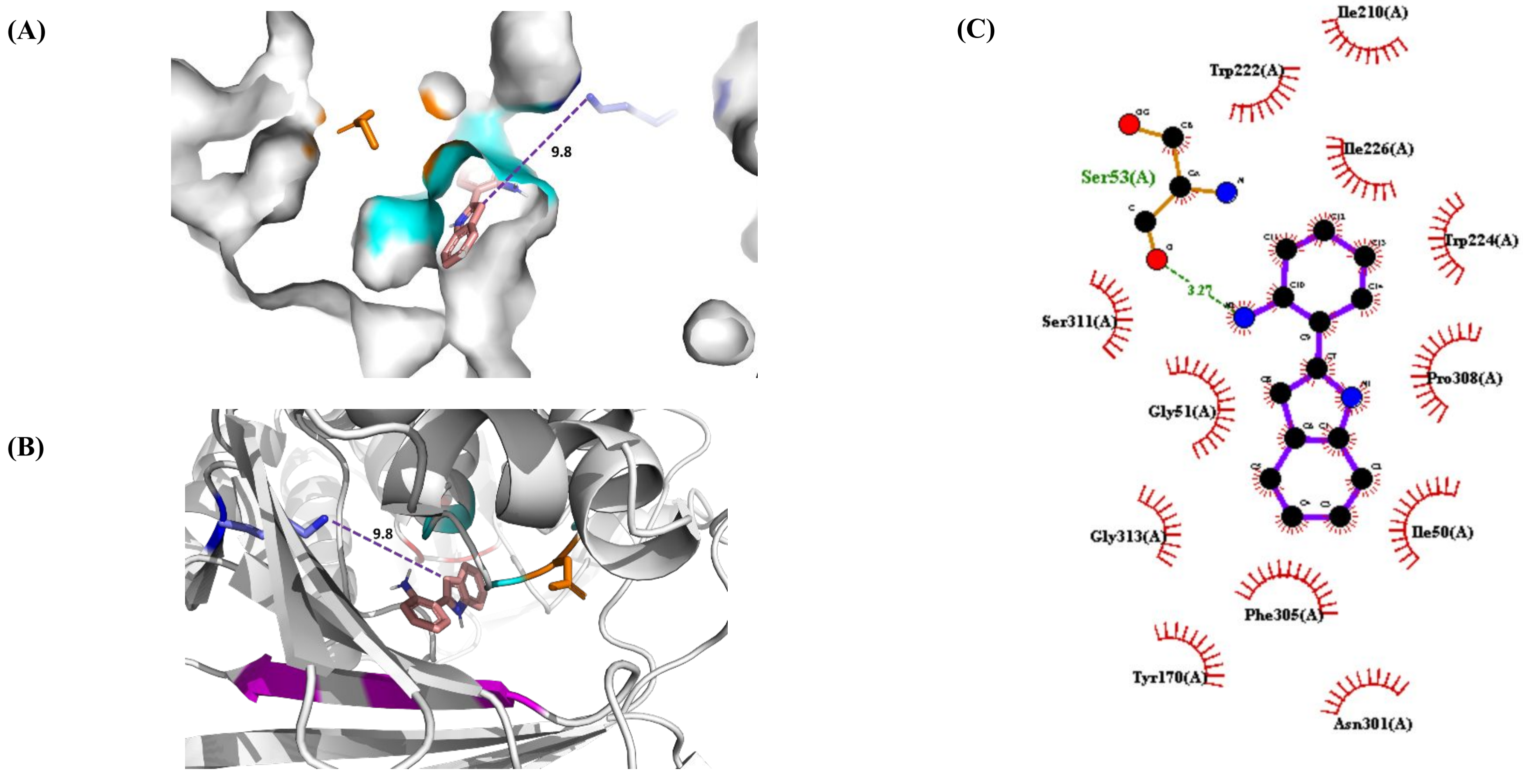

Figure S9. Structural overview of CHEESY1 substrate binding site with bound compound **3**.

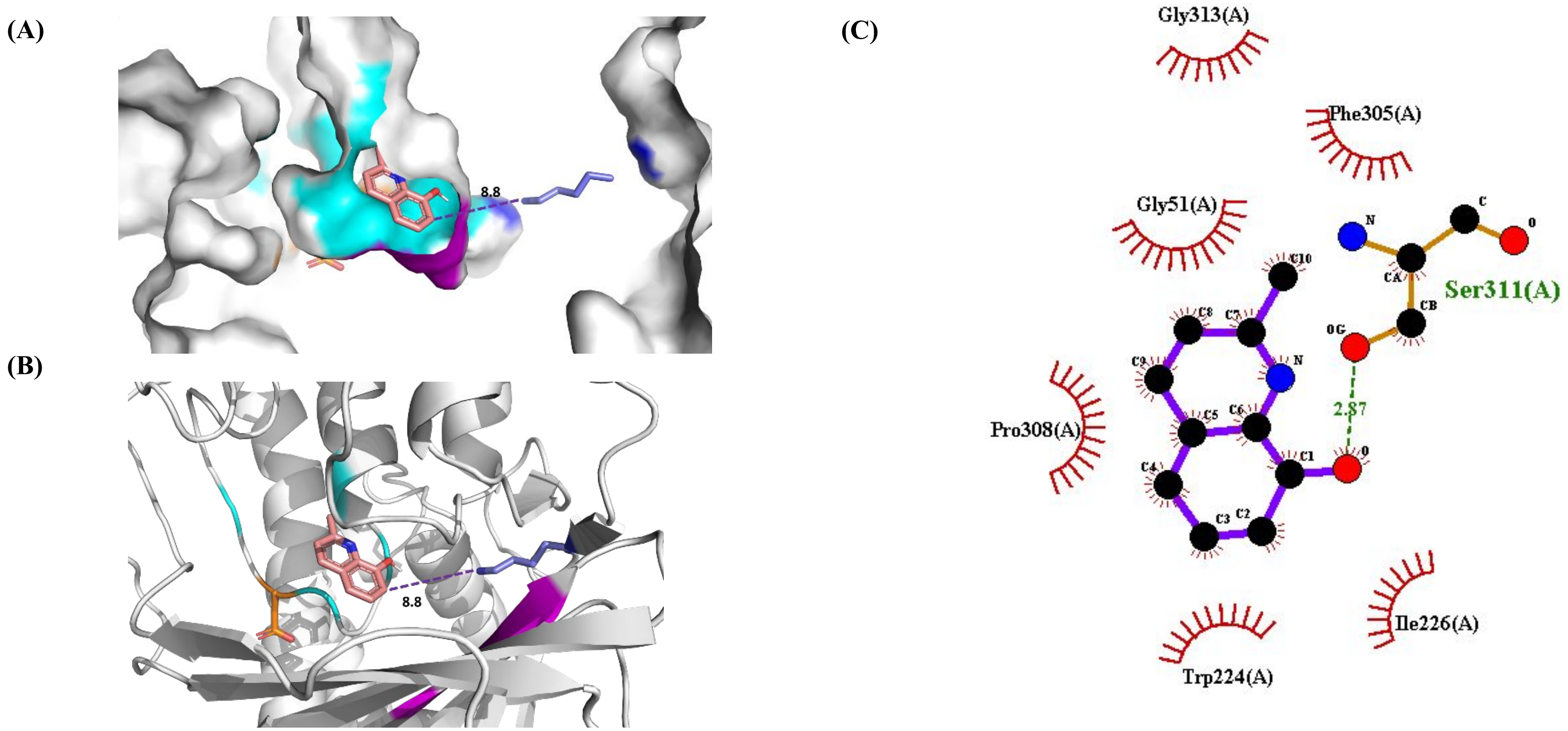

Figure S10. Structural overview of CHEESY1 substrate binding site with bound compound **4**.

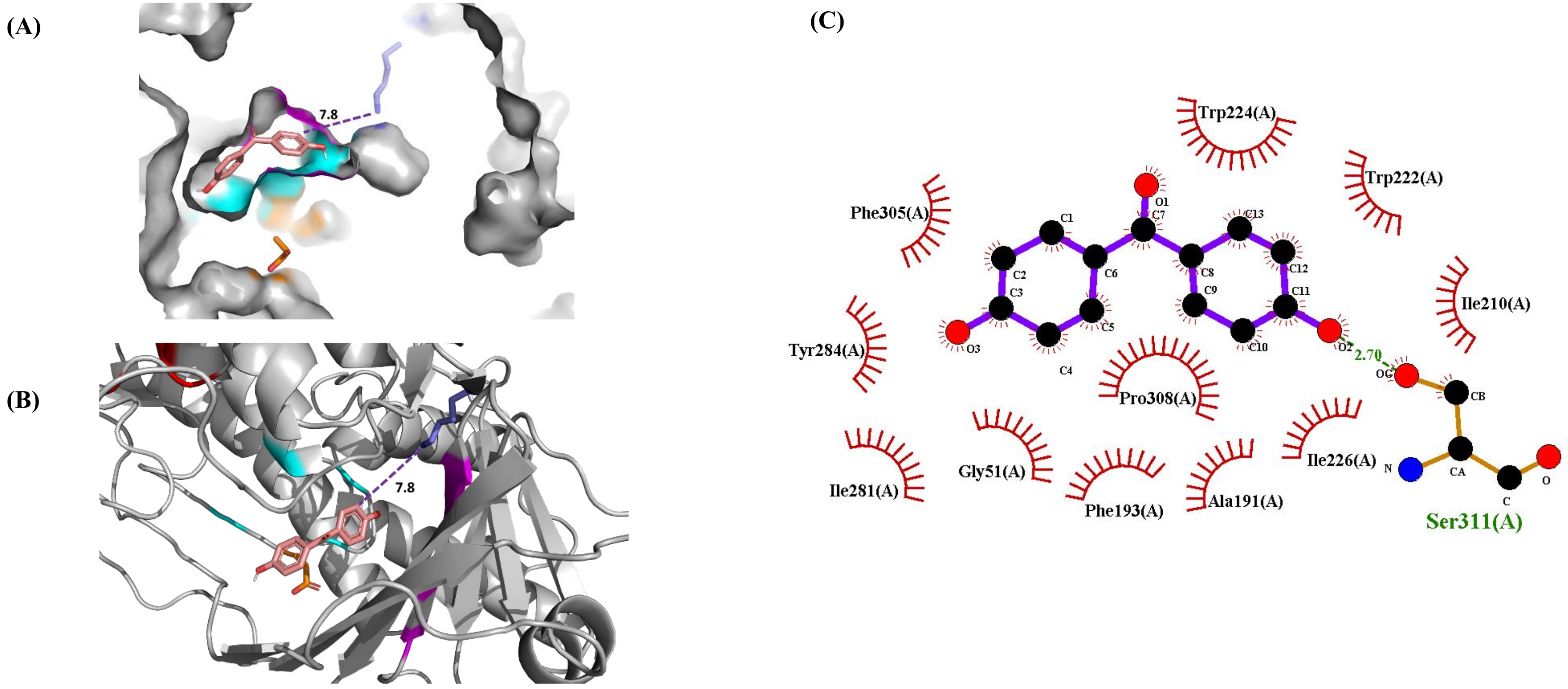

Figure S11. Structural overview of CHEESY1 substrate binding site with bound compound **5**.

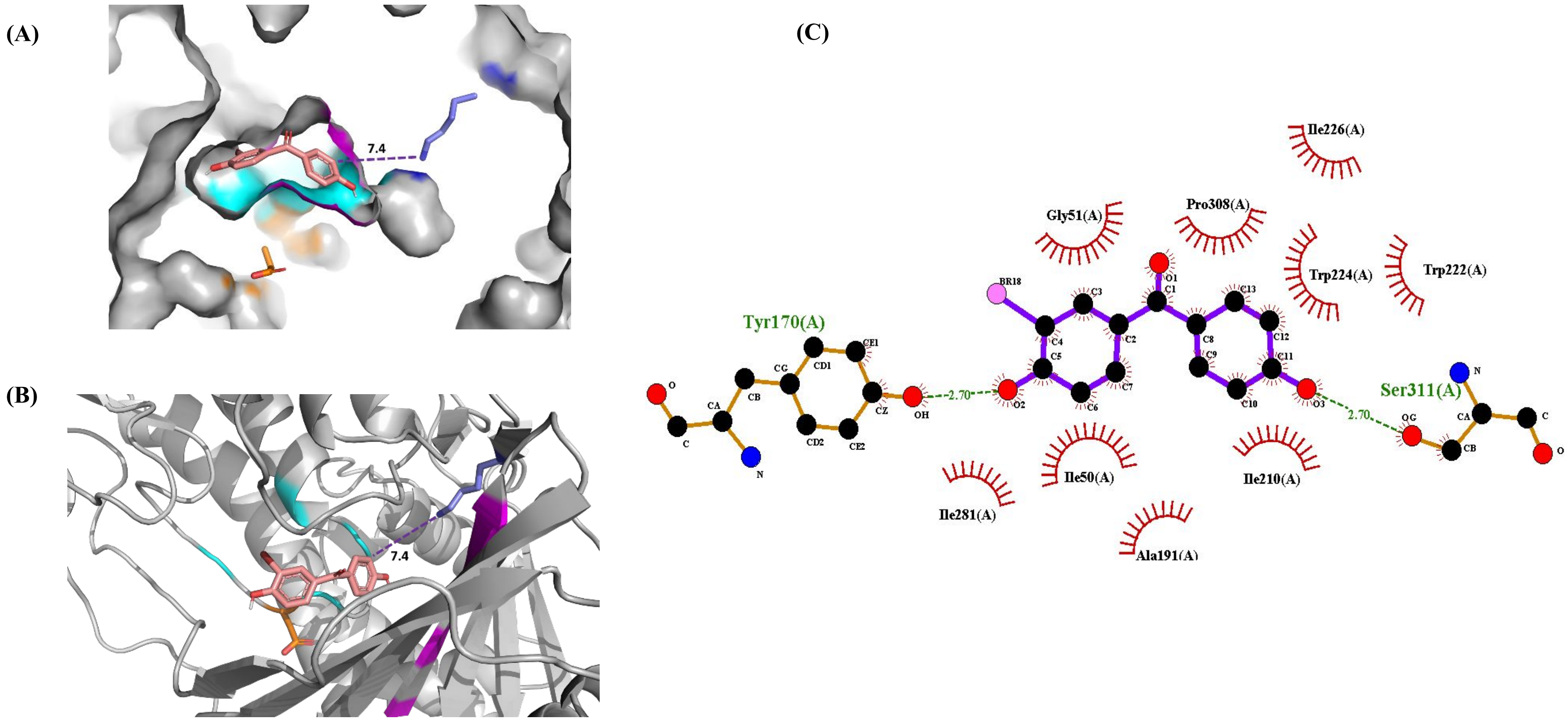

Figure S12. Structural overview of CHEESY1 substrate binding site with bound compound **5a.** For (A)-(B), Bromine atom was coloured in brown. (C) Bromine atom was coloured in pink.

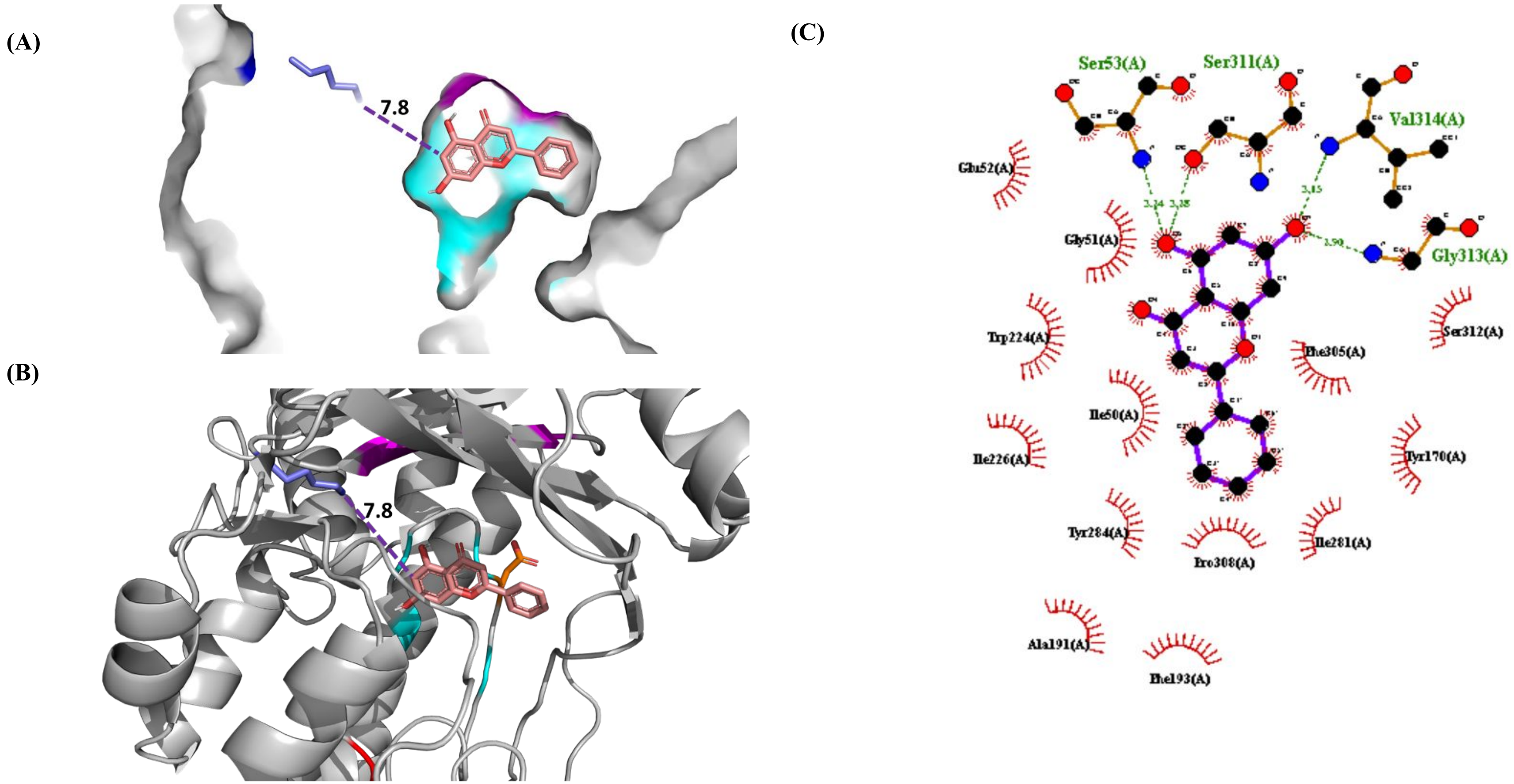

Figure S13. Structural overview of CHEESY1 substrate binding site with bound compound **6**.

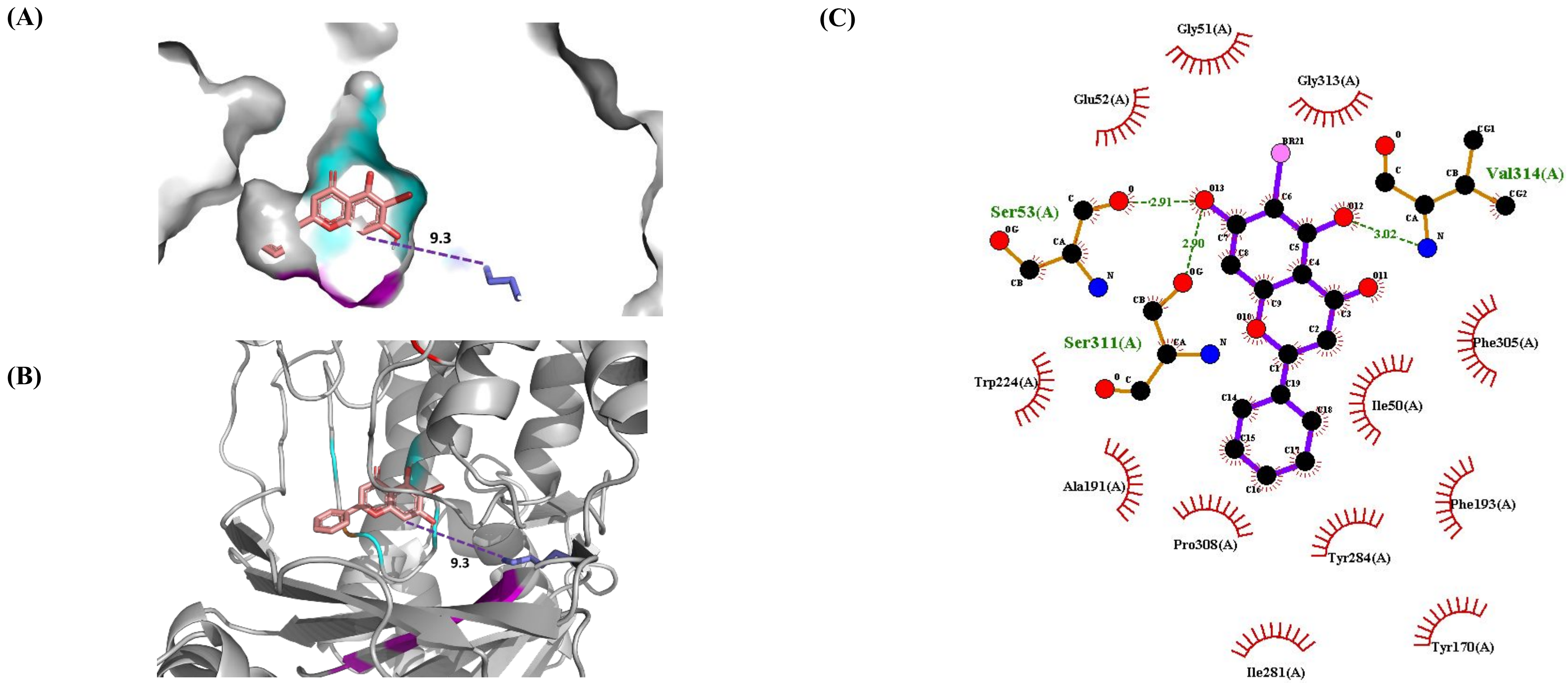

Figure S14. Structural overview of CHEESY1 substrate binding site with bound compound **6a.** For A-B, Bromine atom was coloured in brown. (C), Bromine atom was coloured in pink.

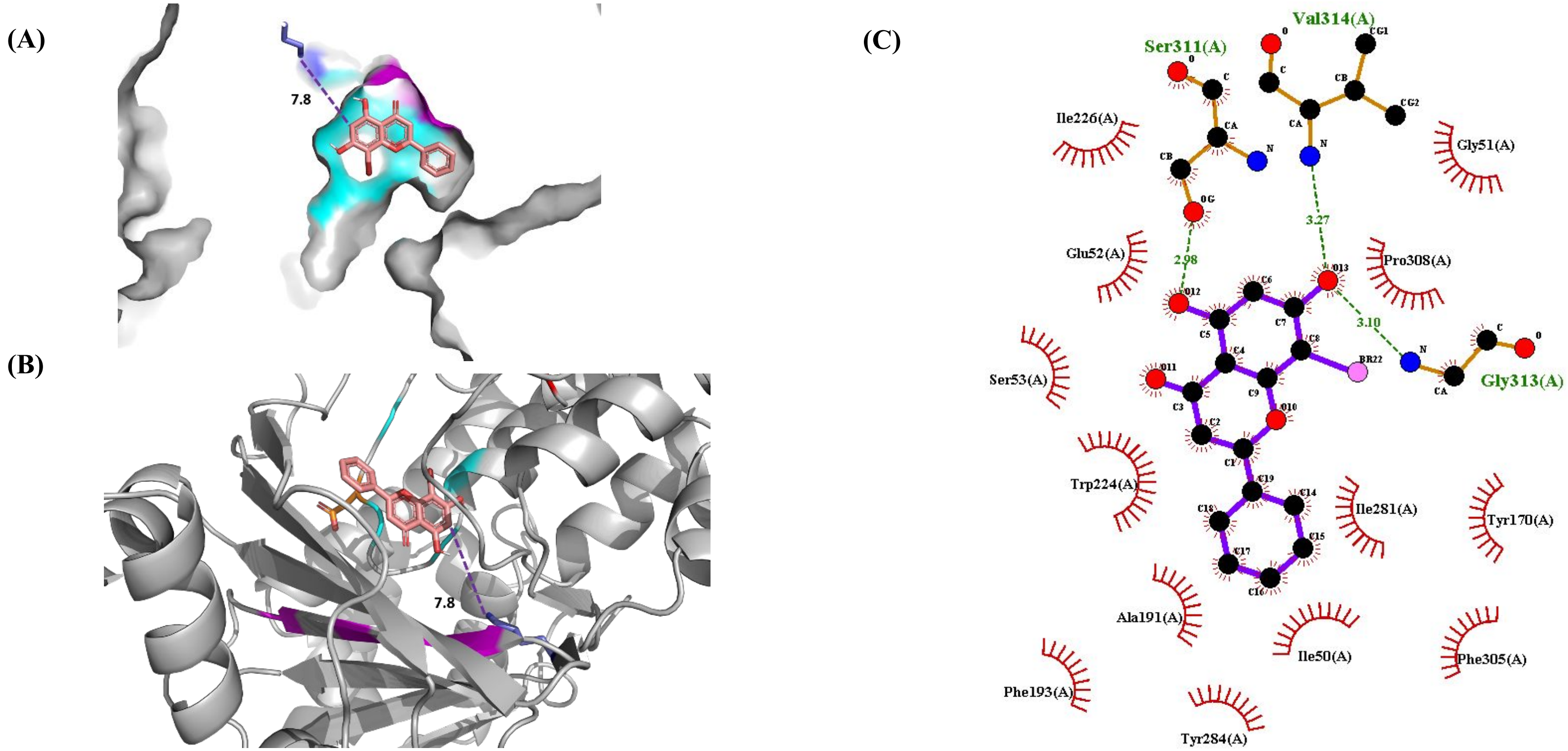

Figure S15. Structural overview of CHEESY1 substrate binding site with bound compound **6b**. For A-B, Bromine atom was coloured in brown. (C), Bromine atom was coloured in pink.

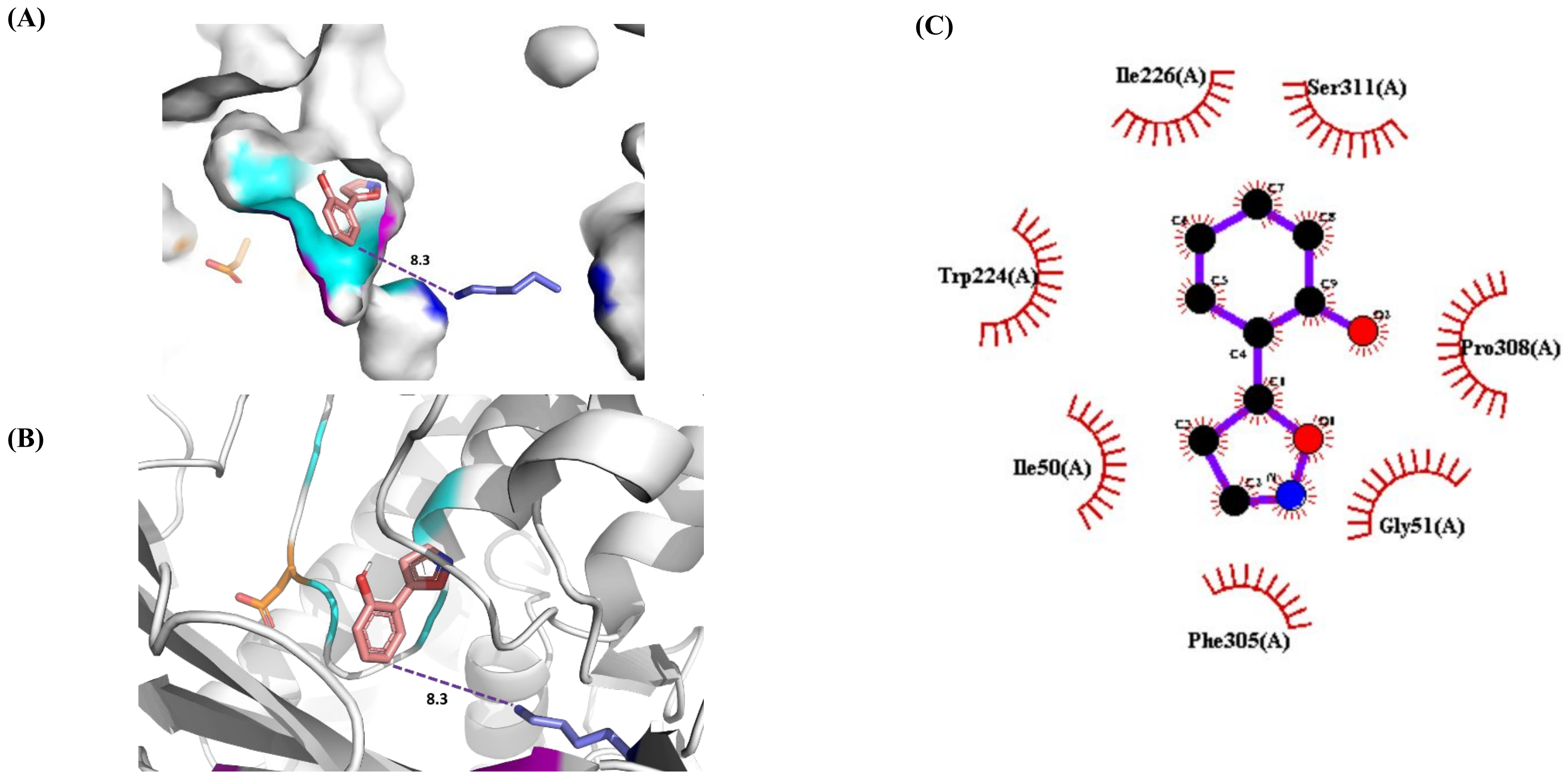

Figure S16. Structural overview of CHEESY1 substrate binding site with bound compound **7** with para position (X_2) to generate **7b.**

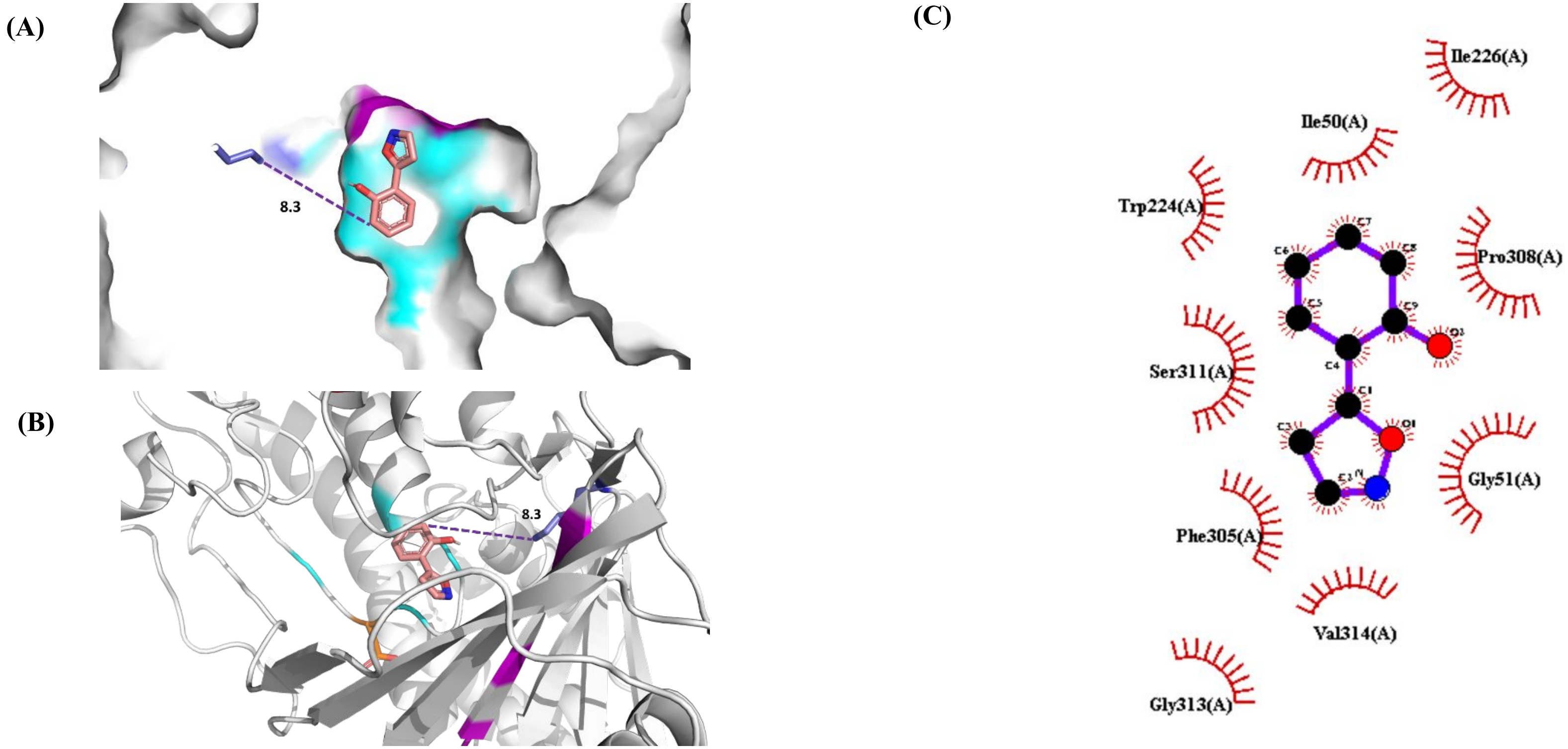

Figure S17. Structural overview of CHEESY1 substrate binding site with bound compound **7** with ortho position (X_1) generating **7a**.

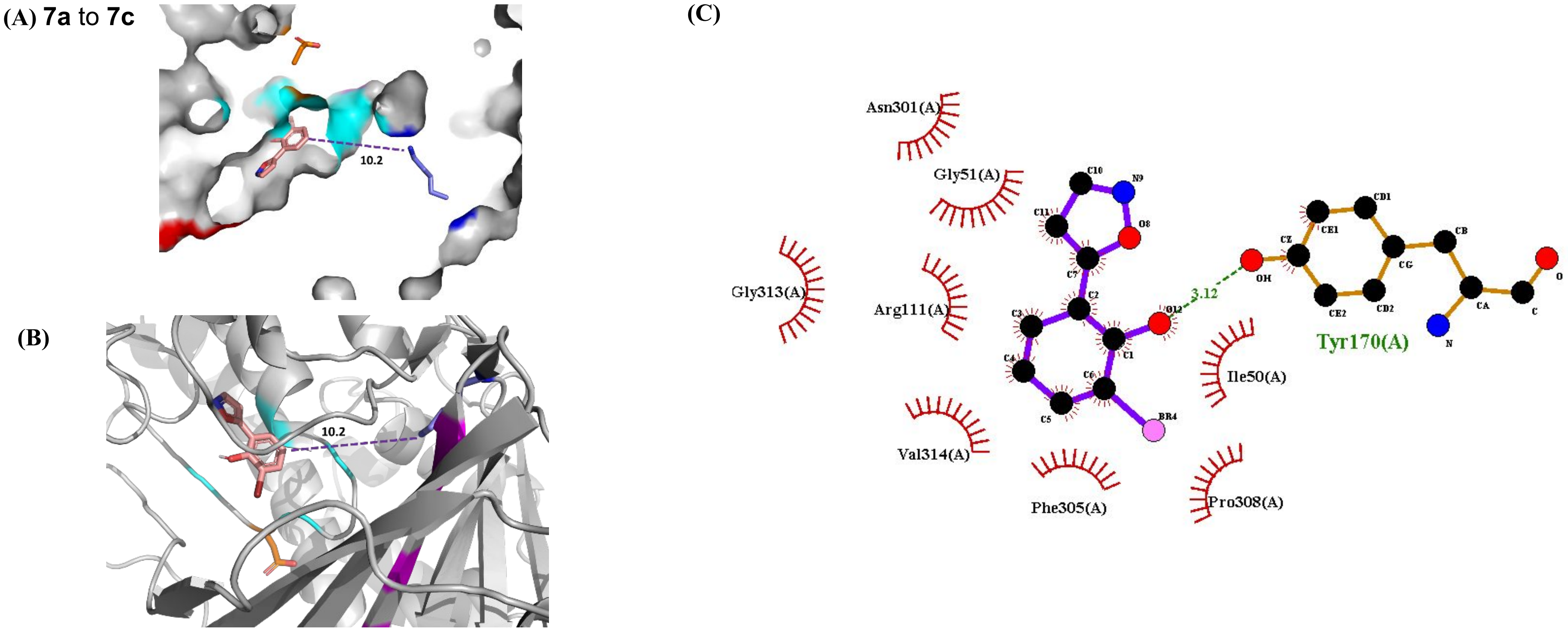

Figure S18. Structural overview of CHEESY1 substrate binding site with bound compound **7a** from flexible docking to generate **7c**. For A-B, Bromine atom was coloured in brown. (C), Bromine atom was coloured in pink.

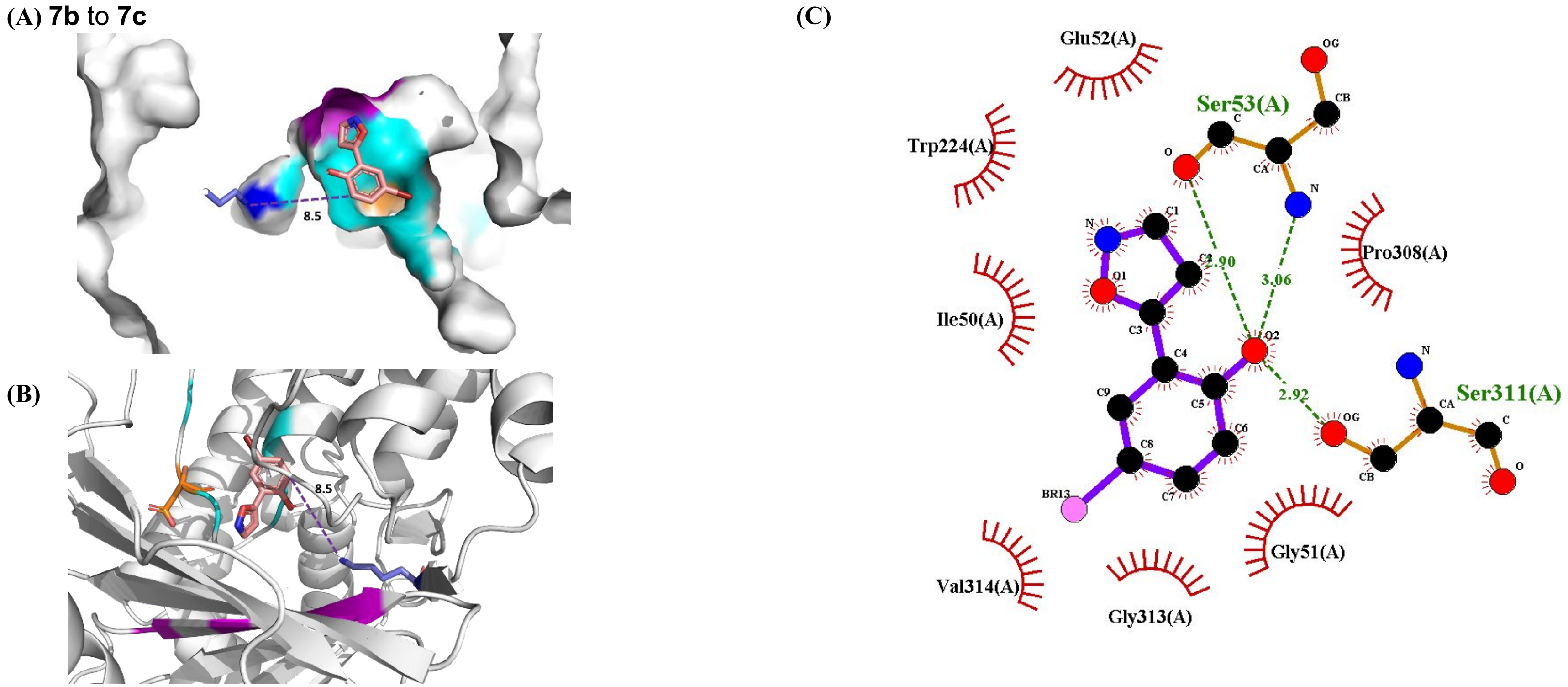

Figure S19. Structural overview of CHEESY1 substrate binding site with bound compound **7b.** For A-B, bromine atom was coloured in brown. (C), Bromine atom was coloured in pink.

3.3 Gene String Information of CHEESY1

Gene encoding CHEESY1 was synthesized by GeneArt (Thermo Fisher Scientific) with codon optimization for heterologous expression in *E. coli* BL21(DE3). The nucleotide and translated amino acid sequences are provided in Figure S20.

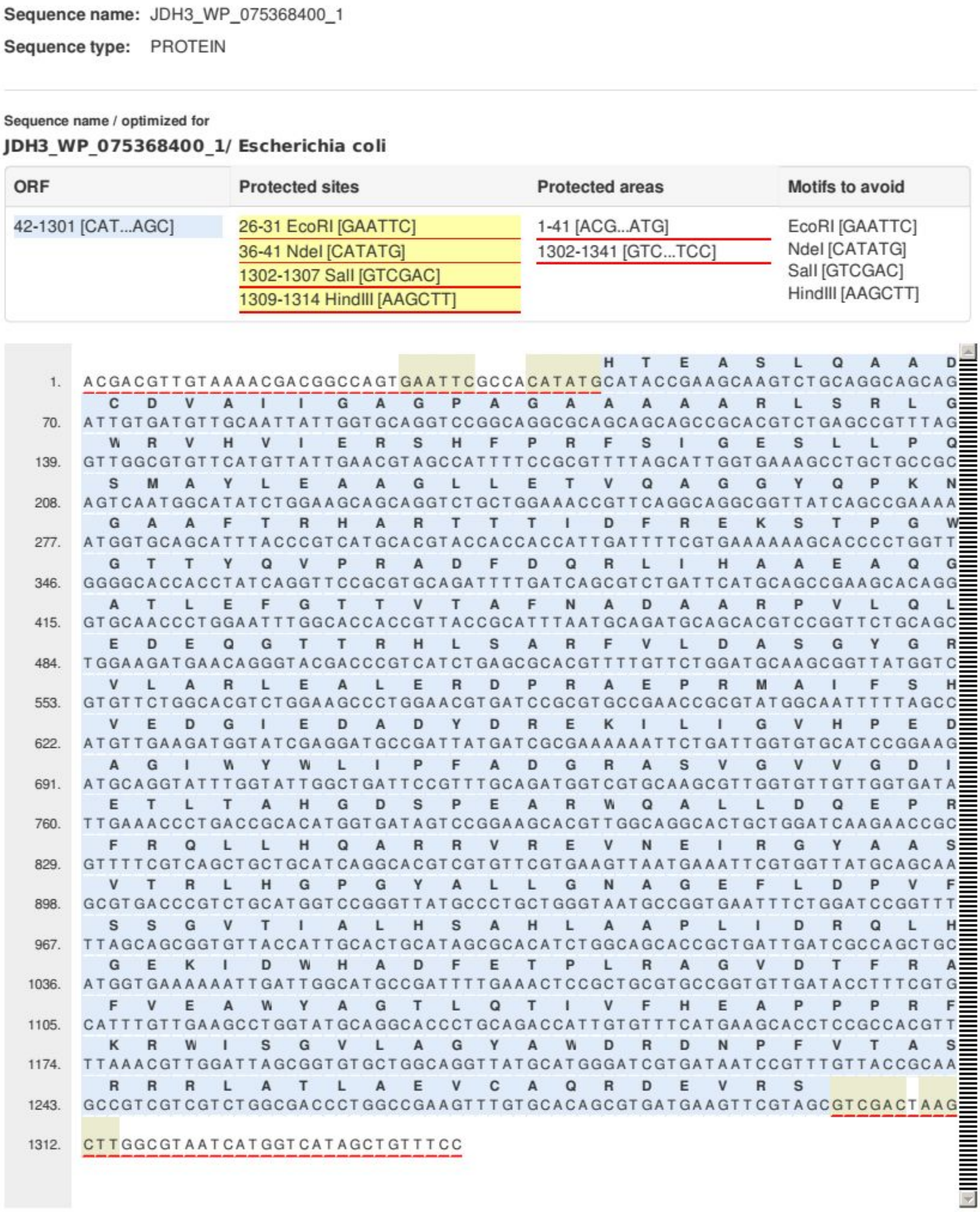

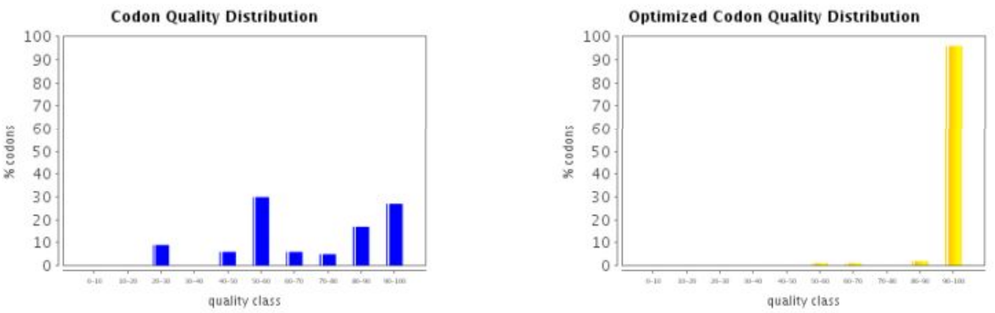

The histograms show the percentage of sequence codons which fall into a certain quality class. The quality value of the most frequently used codon for a given amino acid in the desired expression system is set to 100, the remaining codons are scaled accordingly (see also Sharp, P.M., Li, W.H., Nucleic Acids Res. 15 (3),1987).

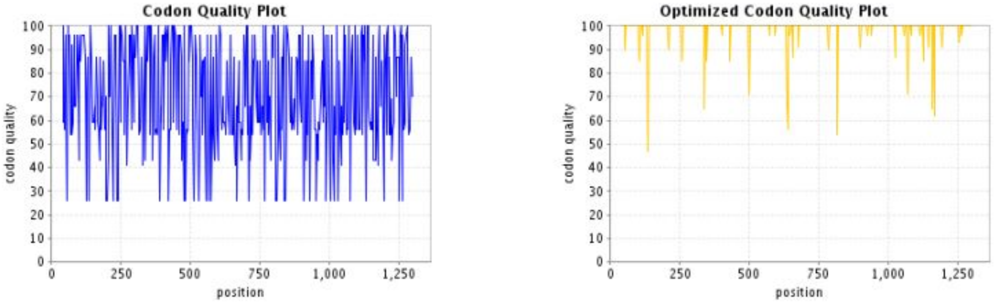

The plots show the quality of the used codon at the indicated codon position.

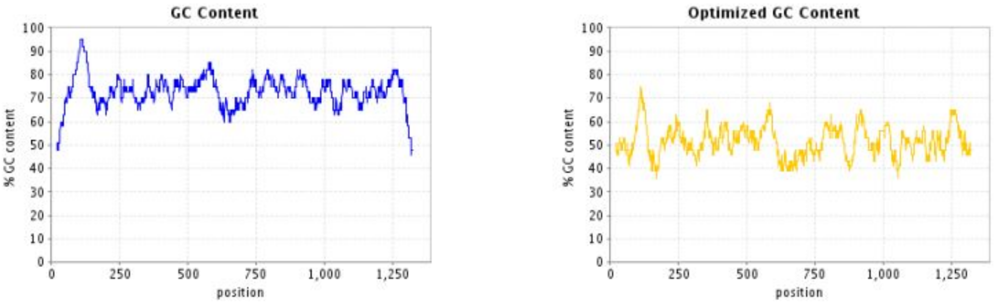

The plots show the GC content in a 40 bp window centered at the indicated nucleotide position.

Figure S20. Synthesised gene string information of CHEESY1.

3.4 SDS-PAGE of CHEESY1 and PrnF

Pure CHEESY1 yielded a concentration of 300-500 µM and was stored at -80 ℃. PrnF also produced an acceptable yield, with a stock concentration of 100-200 µM.

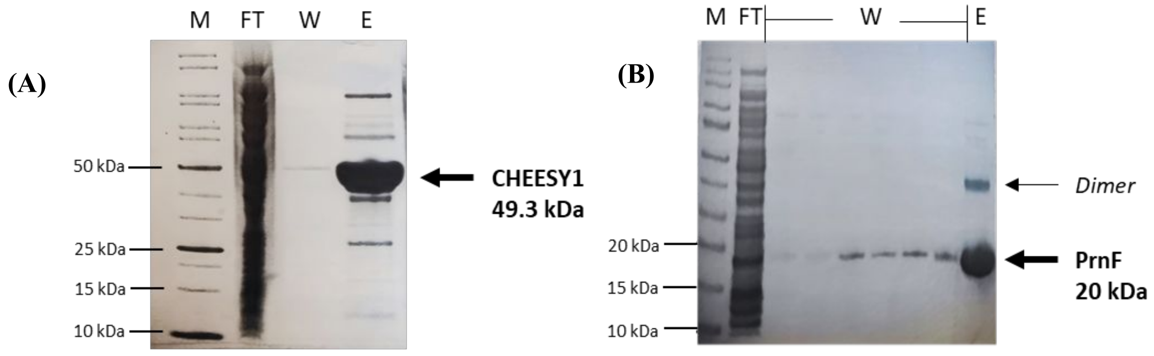

Figure S21. (A): SDS-PAGE (10%) of CHEESY1; (B): SDS-PAGE (10%) of PrnF. M: NEB unstained protein ladder 10-200 kDa.

3.5 UPLC Chromatograms of Substrate **1** to **7**

Unless otherwise indicated, the halogenation assays were conducted with NaBr. Reaction of halogenation assay system: 50 µM CHEESY1, 1 µM PrnF, 10 µM FAD, 2.5 mM NADH, 10 mM NaBr, 200 U/mL catalase, 10 U/mL SOD, 500 µM substrate, in 50 mM HEPES buffer, pH 8.0. Control was set up with no CHEESY1 added in. The assay system for control and reaction is shown in Table S6.

UPLC chromatograms of CHEESY1 against 7 substrates and controls (with no CHEESY1 in) are shown in Figure S22-S29.

Table S6. Assay system for control and reaction with substrate **1** to **7**.

Control (Ctr)	Reaction (Rxn)	Concentration	Conditions
X	CHEESY1	50 µM	Total volume of 100 µL, incubate at 30 °C overnight. Quench with 100 µL MeOH. Analyse clarified reaction mixture on UPLC.
Substrate	Substrate	500 µM	
PrnF	PrnF	1 µM	
FAD	FAD	10 µM	
NADH	NADH	2.5 mM	
NaBr	NaBr	10 mM	
Catalase	Catalase	200 U/mL	
SOD	SOD	10 U/mL	
HEPES buffer	HEPES buffer	50 mM	

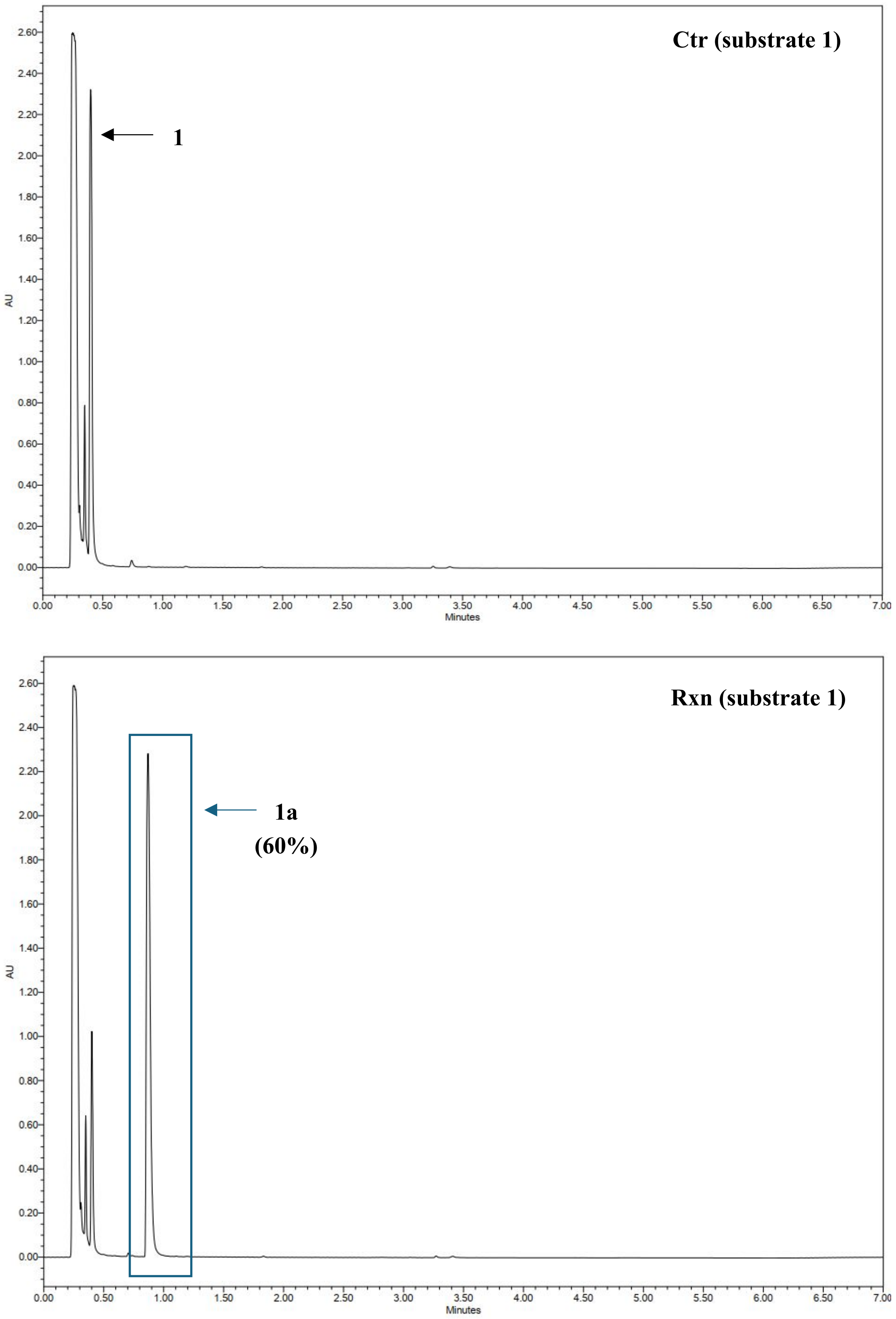

Figure S22. UPLC chromatogram of small-scale bromination on substrate 6-hydroxyquinoline **1**. The product 5-Bromo-6-hydroxyquinoline **1a** is highlighted with a blue box and a blue arrow.

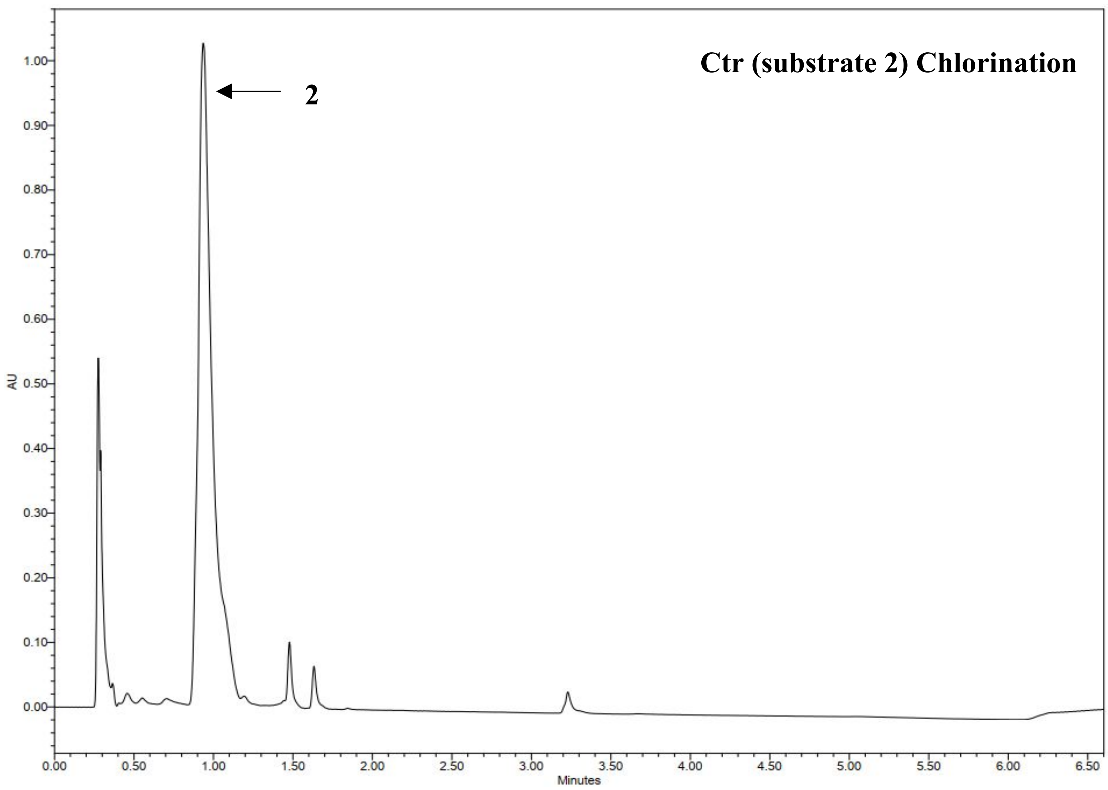

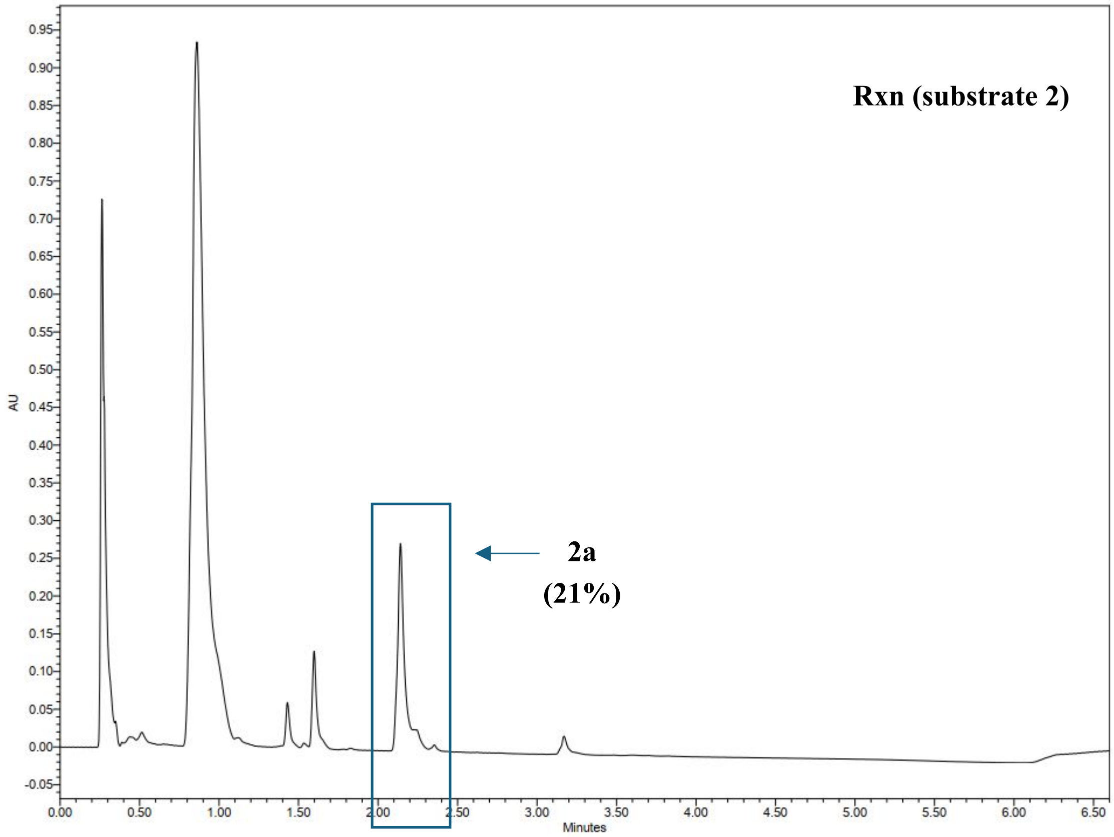

Figure S23. UPLC chromatogram of small-scale chlorination on substrate 5-amino-3-methyl-1-phenylpyrazole **2**. The 4-chloro-3-methyl-1-phenyl-1H-pyrazol-5-amine **2a** is highlighted with a blue box and a blue arrow.

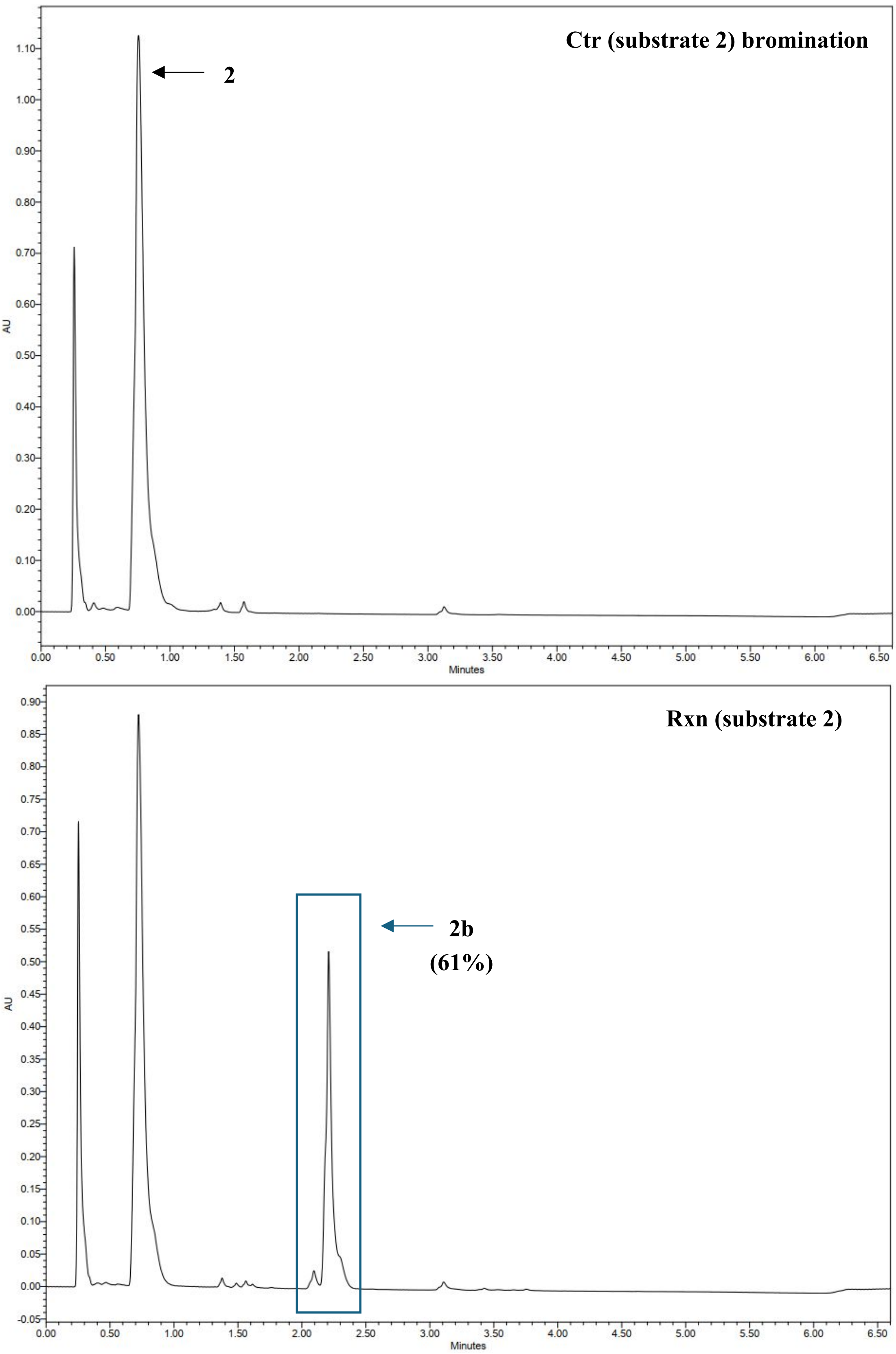

Figure S24. UPLC chromatogram of small-scale bromination on substrate 5-amino-3-methyl-1-phenylpyrazole **2**. The product 4-bromo-3-methyl-1-phenyl-1H-pyrazol-5-amine **2b** is highlighted with a blue box and a blue arrow.

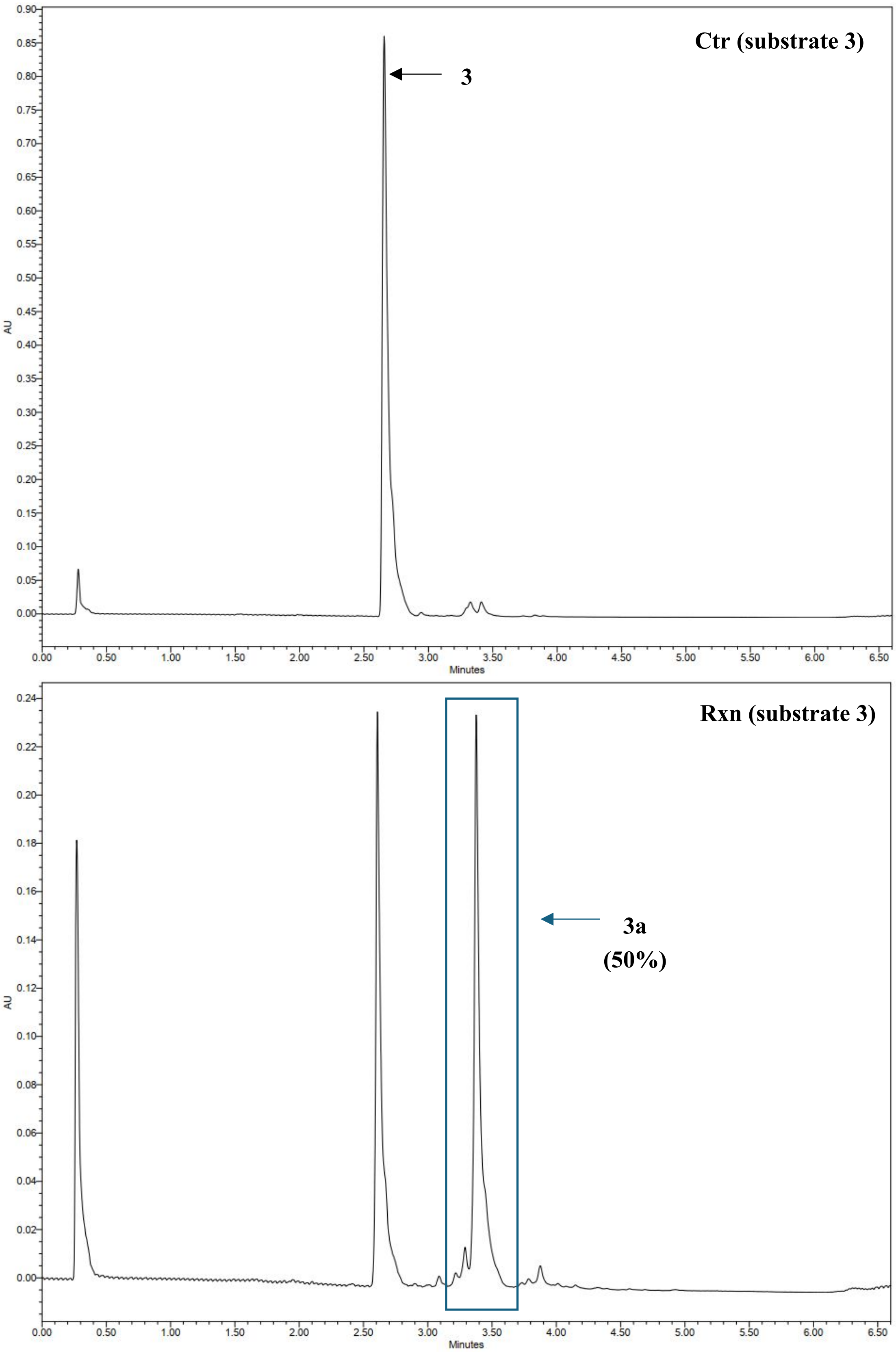

Figure S25. UPLC chromatogram of small-scale bromination on substrate 2-(1*H*-indol-2-yl)aniline **3**. The product 2-(3-bromo-1H-indol-2-yl)aniline **3a** is highlighted with a blue box and a blue arrow.

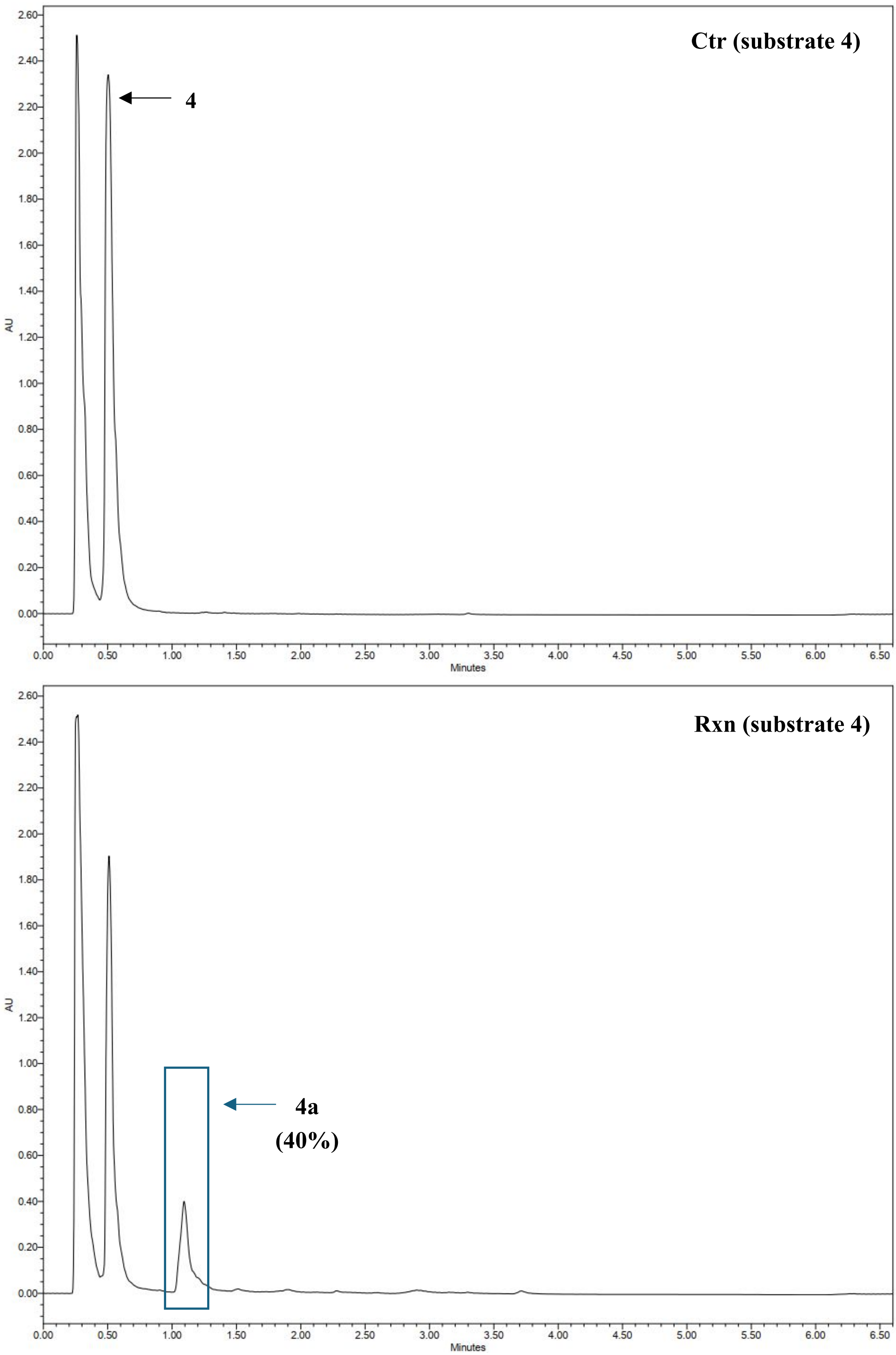

Figure S26. UPLC chromatogram of small-scale bromination on substrate 2-methyl-8-quinolinol **4**. The product 7-bromo-2-methylquinolin-8-ol **4a** is highlighted with a blue box and a blue arrow.

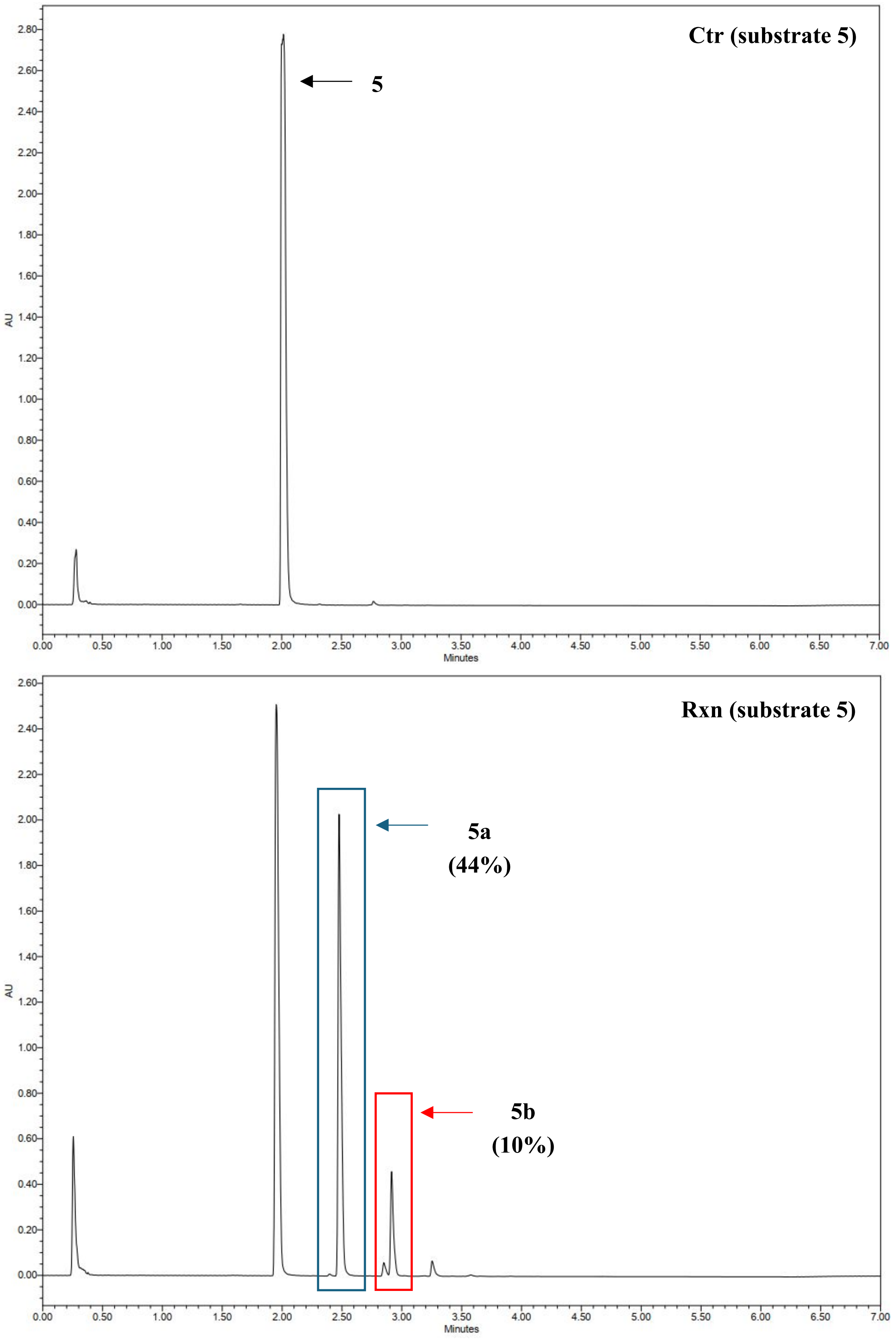

Figure S27. UPLC chromatogram of small-scale bromination on substrate 44'-dihydroxybenzophenon **5**. The product (3-bromo-4-hydroxyphenyl)(4-hydroxyphenyl)methanone **5a** is highlighted with a blue box and a blue arrow. Bis(3-bromo-4-hydroxyphenyl)methanone **5b** is highlighted with a red box and a red arrow.

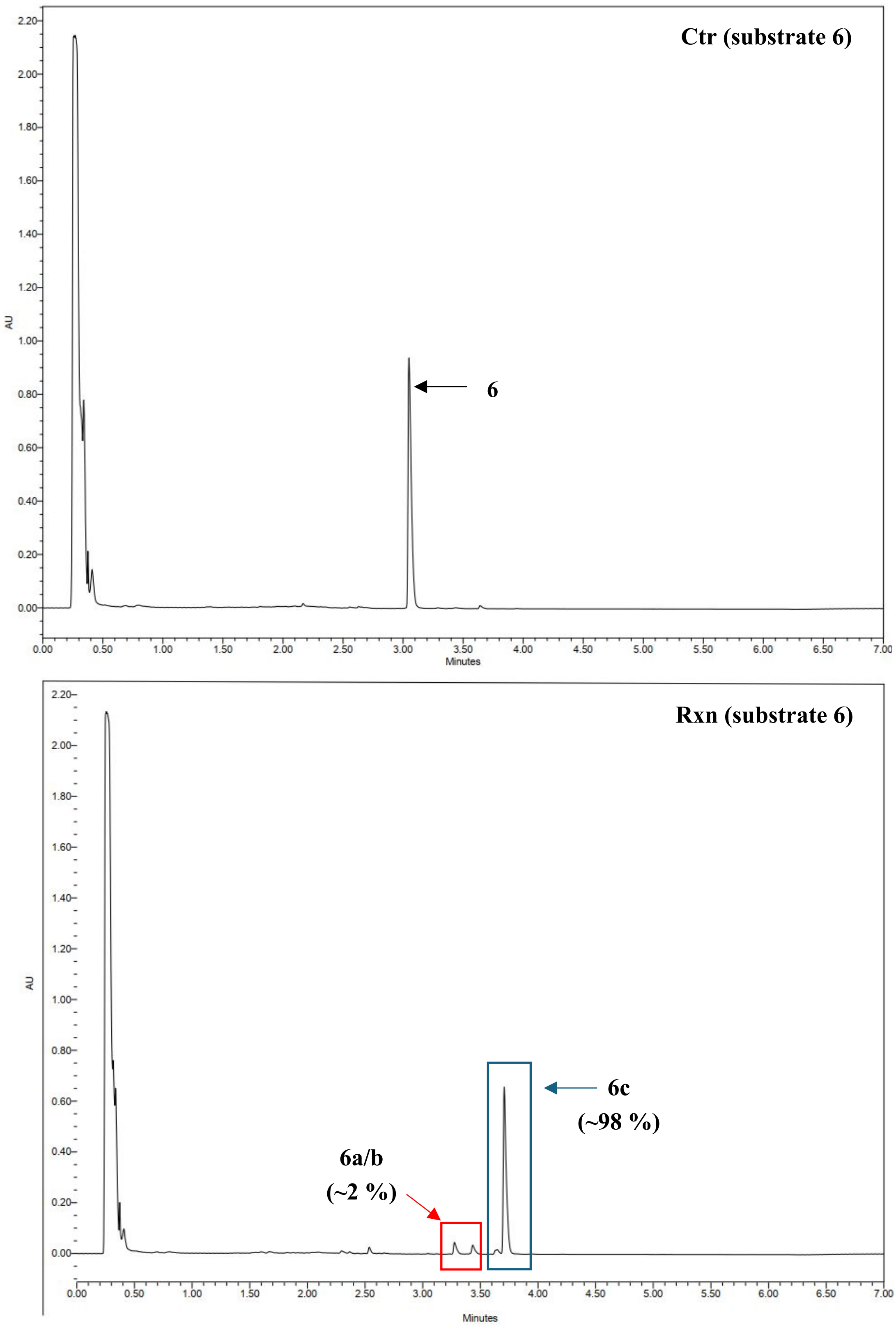

Figure S28. UPLC chromatogram of small-scale bromination on substrate chrysin **6**. The product 6-Br-chrysin **6a** / 8-Br-chrysin **6b** is highlighted with a red box and a red arrow (these two products were not structurally identified in this study). 6,8-dibromo-5,7-dihydroxy-2-phenyl-4H-chromen-4-one **6c** is highlighted with a blue box and a blue arrow.

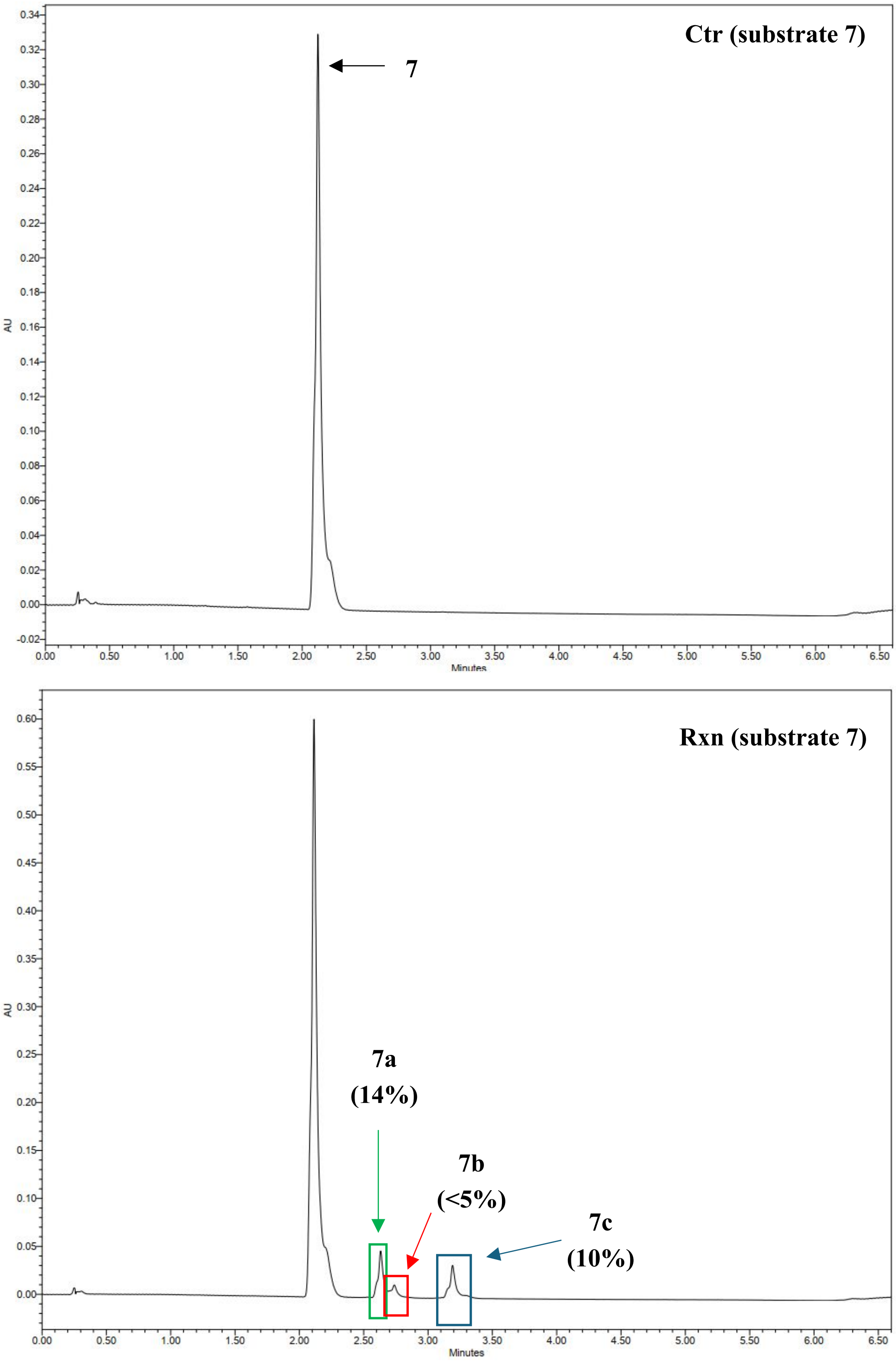

Figure S29. UPLC chromatogram of small-scale bromination on substrate 2-(5-Isoxazolyl)phenol **7**. The product 2-(3-bromo-1H-indol-2-yl)aniline **7a** is highlighted with a green box and a green arrow. 4-Bromo-2-(isoxazol-5-yl)phenol **7b** is highlighted with a red box and a red arrow (these two products were not structurally identified in this study). 2,4-Dibromo-6-(isoxazol-5-yl)phenol **7c** is highlighted with a blue box and a blue arrow.

3.6 LC-MS Interpretation of Halogenation Reactions

Compound	$[M+H]^+$ calculated
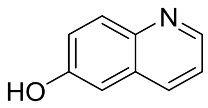	Chemical Formula: $C9H8NO^+$<br>Exact Mass: 146.0600<br>Molecular Weight: 146.1685<br>m/z: 146.0601 (100.0%), 147.0634 (9.7%)
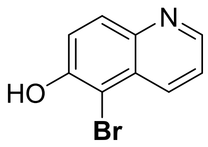	Chemical Formula: $C9H7BrNO^+$<br>Exact Mass: 223.9706<br>Molecular Weight: 225.0645<br>m/z: 223.9706 (100.0%), 225.9686 (97.3%), 224.9740 (9.7%), 226.9719 (9.5%)

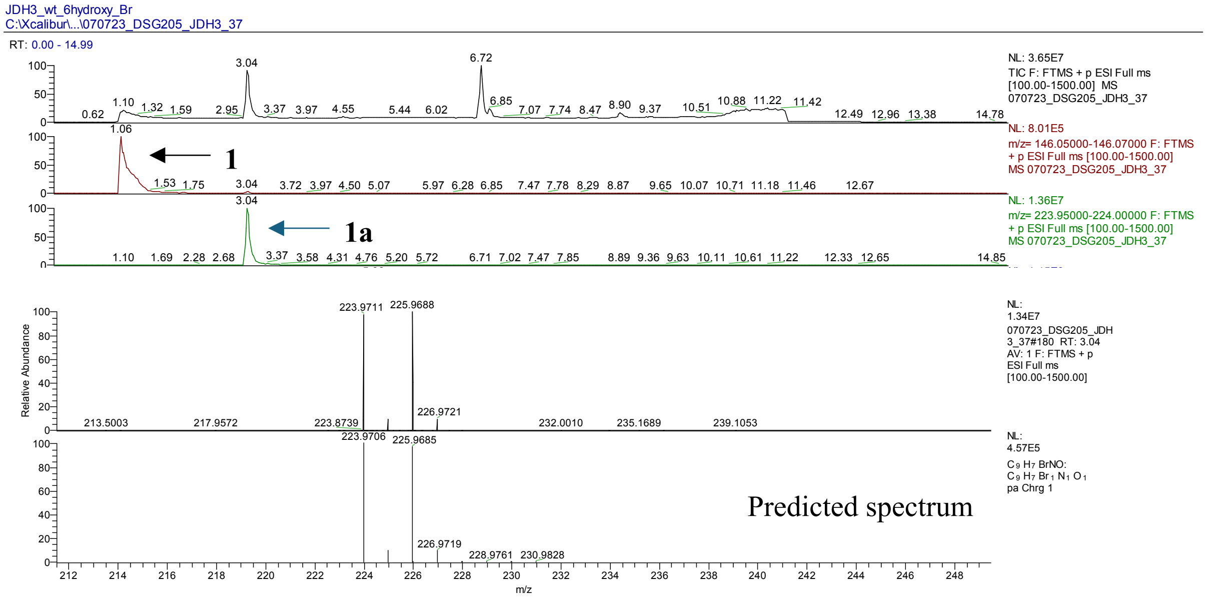

Figure S30. LC-MS analysis of substrate **1** bromination. Only mono-brominated product was observed on EIC chromatogram, while MS confirmed formation of the depicted product. This product was isolated from the biotransformation and fully characterised as **1a**. Regiochemistry was determined by NMR, the product was shown to be brominated at the 5-position. Mass spectrum for **1a** and predicted spectrum comparison are shown at the bottom panel.

Compound	$[M+H]^+$ calculated

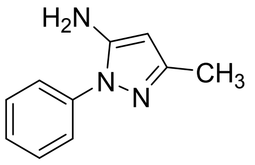	Chemical Formula: $C_{10}H_{12}N3^+$<br>Exact Mass: 174.1026<br>Molecular Weight: 174.2265<br>m/z: 174.1026 (100.0%), 175.1060 (10.8%), 175.0997 (1.1%)
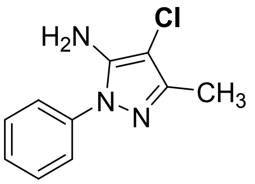	Chemical Formula: $C_{10}H_{11}ClN_3^+$<br>Exact Mass: 208.06<br>Molecular Weight: 208.67<br>m/z: 208.06 (100.0%), 210.06 (32.1%), 209.07 (10.9%), 211.06 (3.8%), 209.06 (1.1%)
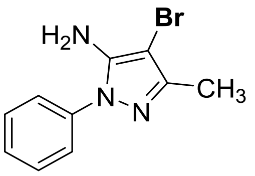	Chemical Formula: $C_{10}H_{11}BrN_3^+$<br>Exact Mass: 252.01<br>Molecular Weight: 253.12<br>m/z: 252.01 (100.0%), 254.01 (97.4%), 255.01 (11.6%), 253.02 (10.9%), 253.01 (1.1%)

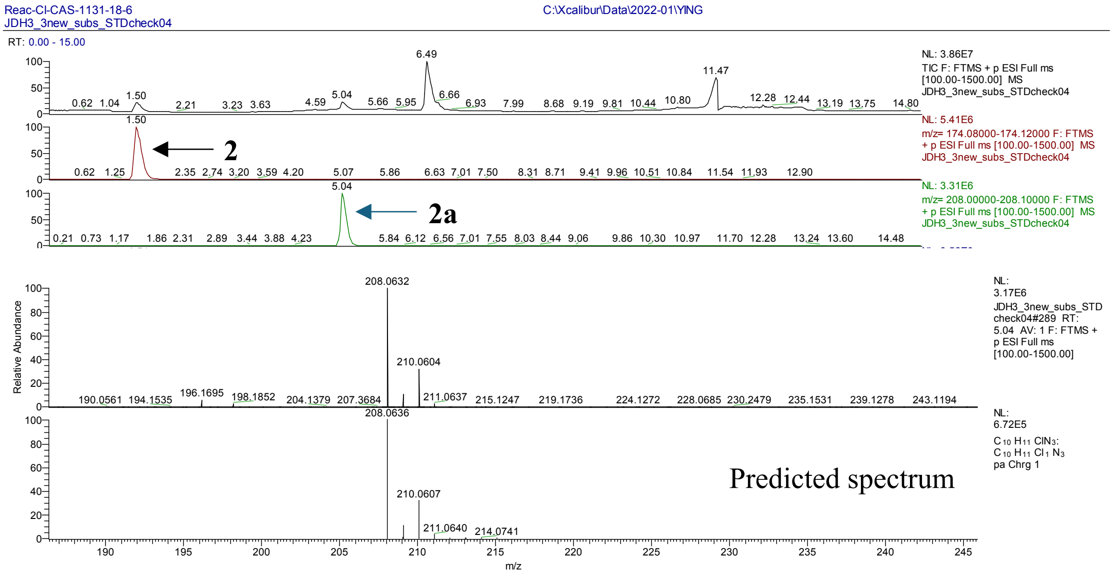

Figure S31. LC-MS analysis of substrate **2** chlorination. Only mono-chlorinated product was observed on EIC chromatogram, while MS confirmed formation of the depicted product. This product was isolated from the biotransformation and fully characterised as **2a**. Regiochemistry was determined by NMR, the product was shown to be chlorinated at the 4-position. Mass spectrum for **2a** and predicted spectrum comparison are shown at the bottom panel.

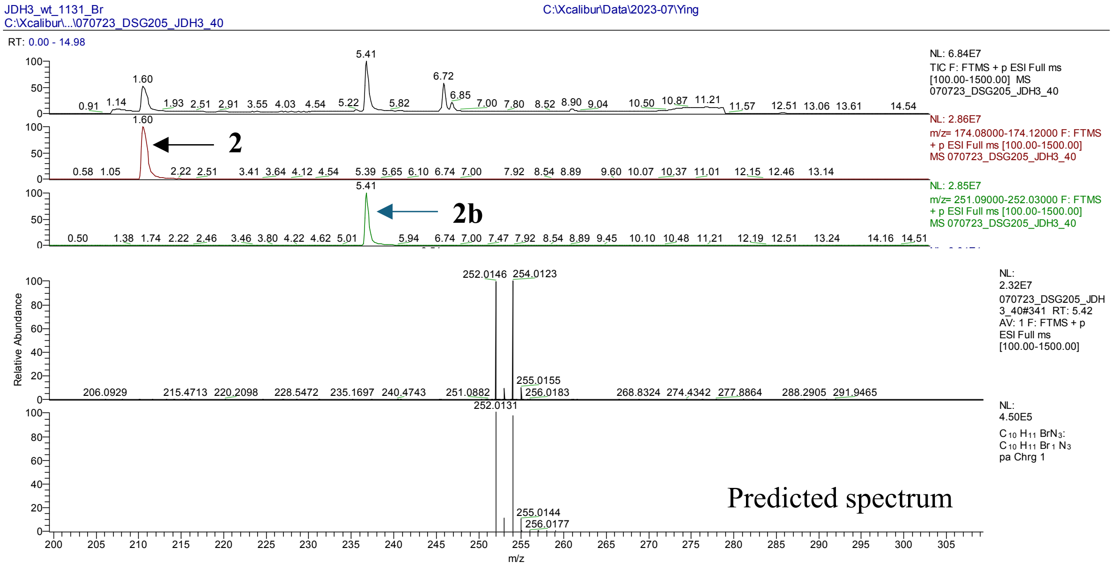

Figure S32. LC-MS analysis of substrate **2** bromination. Major mono-brominated product and a tracing amount of di-brominated product were observed on EIC chromatogram. Mono-brominated product was isolated from the biotransformation and fully characterised as **2b**. Regiochemistry was determined by NMR, the product was shown to be brominated at the 4-position. Mass spectrum for **2b** and predicted spectrum comparison are shown at the bottom panel.

Compound	$[M+H]^+$ calculated
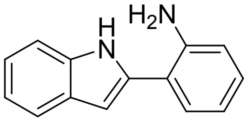	Chemical Formula: $C_{14}H_{13}N_2^+$<br>Exact Mass: 209.1073<br>Molecular Weight: 209.2715<br>m/z: 209.1074 (100.0%), 210.1107 (15.1%), 211.1141 (1.1%)
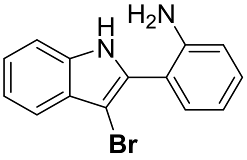	Chemical Formula: $C_{14}H_{12}BrN_2^+$<br>Exact Mass: 287.02<br>Molecular Weight: 288.17<br>m/z: 287.02 (100.0%), 289.02 (97.4%), 288.02 (16.0%), 290.02 (14.9%), 291.02 (1.1%), 289.03 (1.1%)

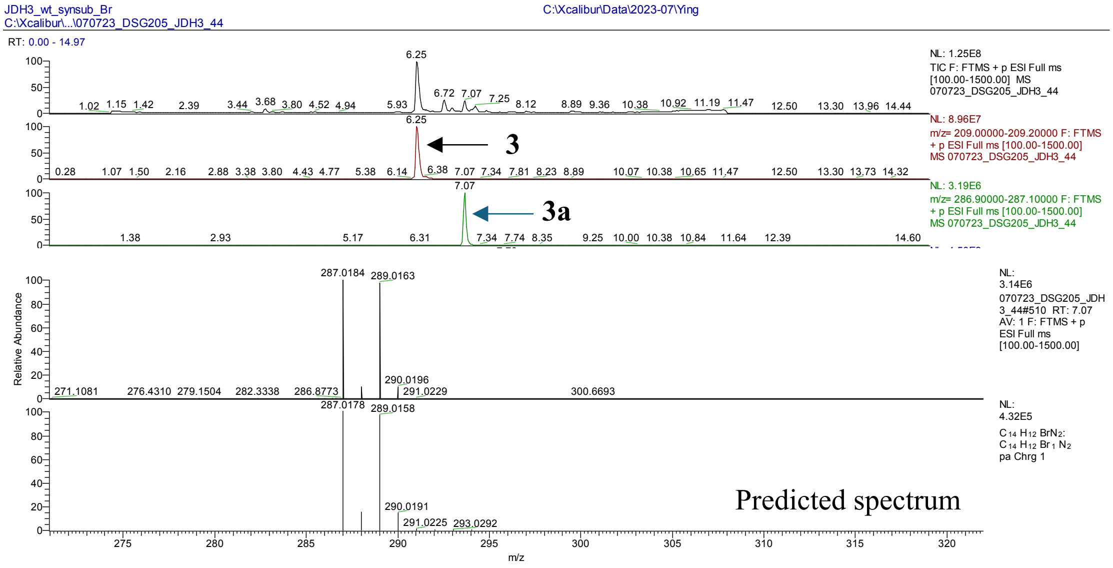

Figure S33. LC-MS analysis of substrate **3** bromination. Mono-brominated product was observed on EIC chromatogram. Mono-brominated product was isolated from the biotransformation and fully characterised as **3a**. Regiochemistry was determined by NMR, the product was shown to be brominated at the 3-position. Mass spectrum for **3a** and predicted spectrum comparison are shown at the bottom panel.

Compound	$[M+H]^+$ calculated
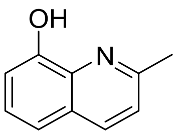	Chemical Formula: $C_{10}H_{10}NO^+$<br>Exact Mass: 160.0757<br>Molecular Weight: 160.1955<br>m/z: 160.0757 (100.0%), 161.0791 (10.8%)
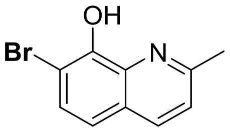	Chemical Formula: $C_{10}H_9BrNO^+$<br>Exact Mass: 237.99<br>Molecular Weight: 239.09<br>m/z: 237.99 (100.0%), 239.98 (97.3%), 238.99 (11.0%), 240.99 (10.7%)

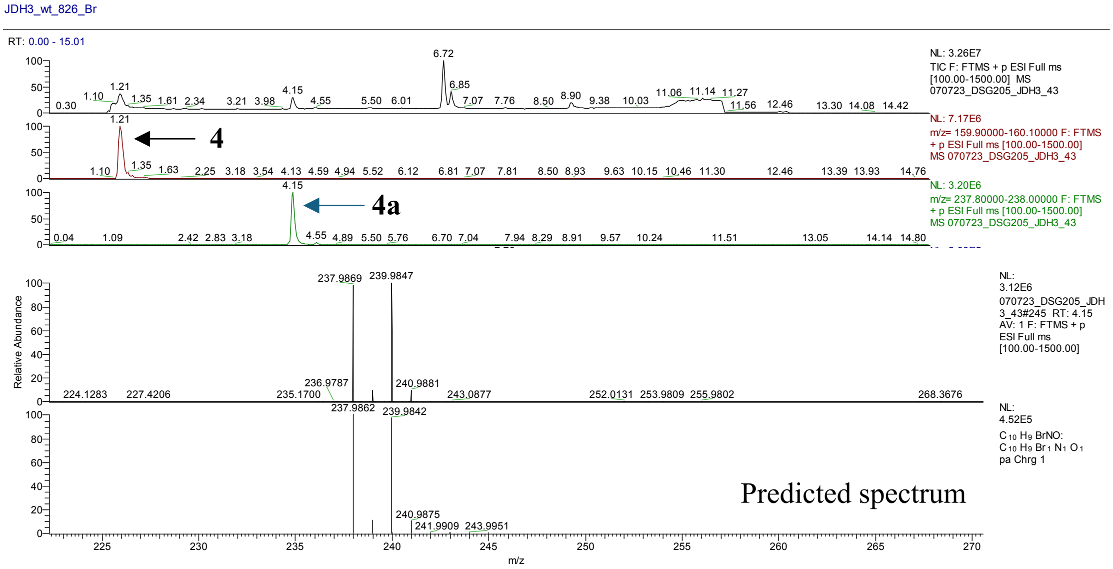

Figure S34. LC-MS analysis of substrate **4** bromination. Mono-brominated product was observed on EIC chromatogram. Mono-brominated product was isolated from the biotransformation and fully characterised as **4a**. Regiochemistry was determined by NMR, the product was shown to be brominated at the 7-position. Mass spectrum for **4a** and predicted spectrum comparison are shown at the bottom panel.

Compound	$[M+H]^+$ calculated

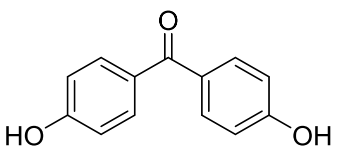	Chemical Formula: $C_{13}H_{11}O_3^+$<br>Exact Mass: 215.0703<br>Molecular Weight: 215.2275<br>m/z: 215.0703 (100.0%), 216.0737 (14.1%)
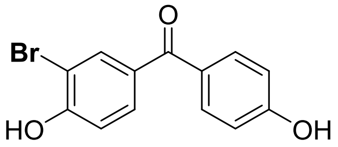	Chemical Formula: $C_{13}H_{10}BrO_3^+$<br>Exact Mass: 292.98<br>Molecular Weight: 294.12<br>m/z: 292.98 (100.0%), 294.98 (97.3%), 293.98 (14.1%), 295.98 (13.8%), 294.99 (1.6%)
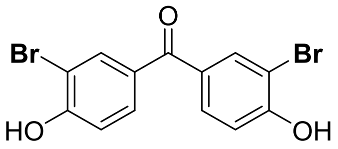	Chemical Formula: $C_{13}H_9Br_2O_3^+$<br>Exact Mass: 370.89<br>Molecular Weight: 373.02<br>m/z: 372.89 (100.0%), 370.89 (51.4%), 374.89 (49.3%), 373.89 (14.2%), 371.90 (7.3%), 375.89 (6.9%)

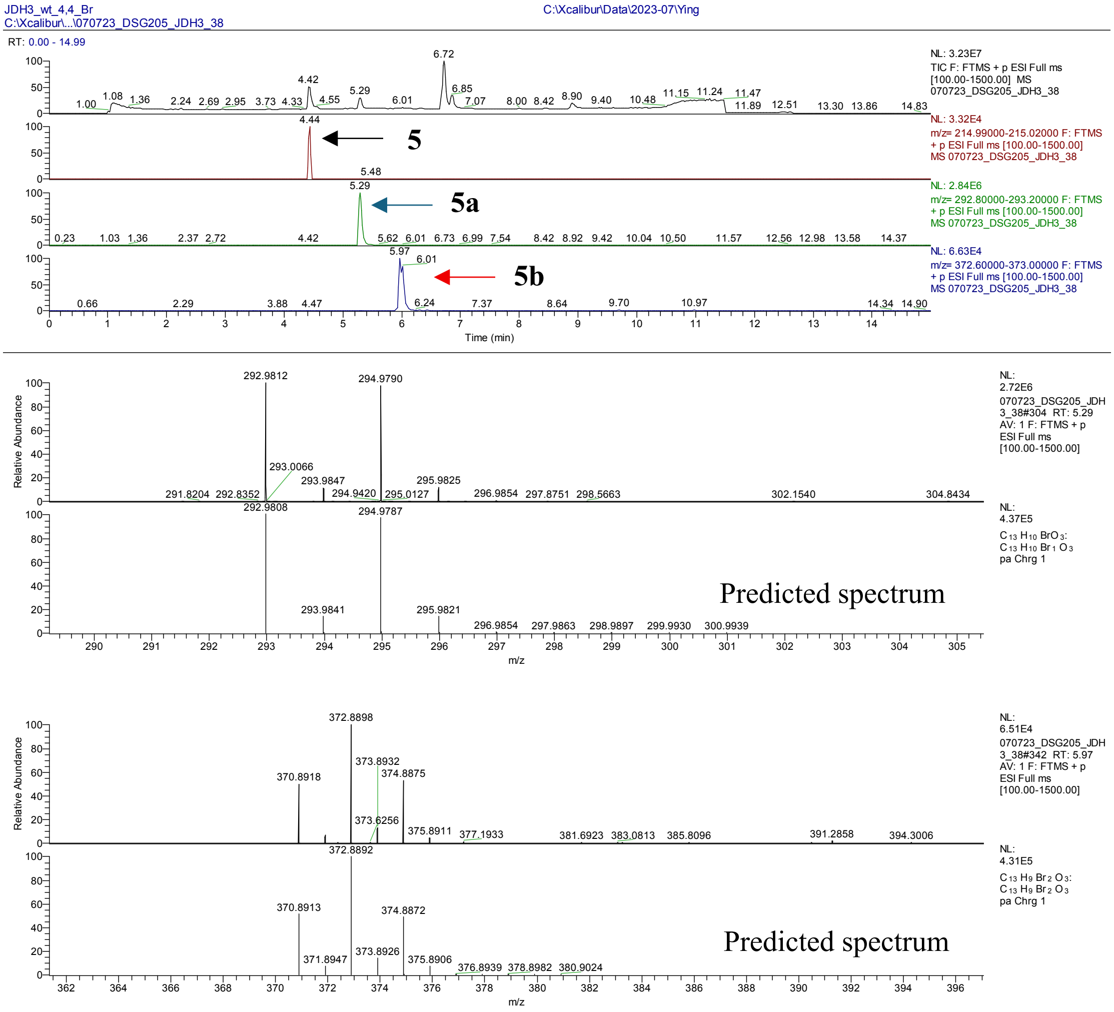

Figure S35. LC-MS analysis of substrate **5** bromination. Mono-brominated product and di-brominated product were observed on EIC chromatogram. Mono-brominated product was isolated from the biotransformation and fully characterised as **5a** and di-brominated product as **5b**.

Regiochemistry was determined by NMR, mass spectra for **5a**, **5b** and predicted spectrum comparison respectively are shown at the bottom panel.

Compound	$[M+H]^+$ calculated
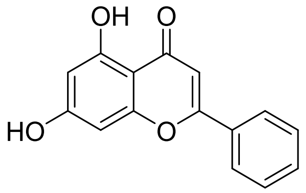	Chemical Formula: $C_{15}H_{11}O^{4+}$<br>Exact Mass: 255.0652<br>Molecular Weight: 255.2485<br>m/z: 255.0652 (100.0%), 256.0686 (16.2%), 257.0719 (1.2%)
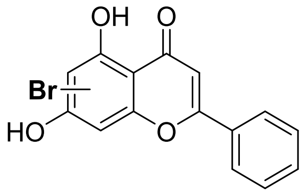	Chemical Formula: $C_{15}H_{10}BrO_4^+$<br>Exact Mass: 332.9757<br>Molecular Weight: 334.1445<br>m/z: 332.9757 (100.0%), 334.9737 (97.3%), 333.9791 (16.2%), 335.9771 (15.8%), 334.9825 (1.2%), 336.9804 (1.2%)
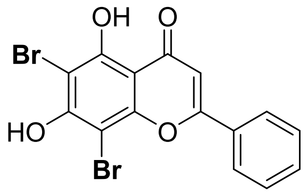	Chemical Formula: $C_{15}H_9Br_2O_4^+$<br>Exact Mass: 412.90<br>Molecular Weight: 415.06<br>m/z: 414.90 (100.0%), 412.90 (51.4%), 416.90 (49.5%), 415.90 (16.4%), 413.91 (8.5%), 417.90 (8.0%), 416.91 (1.3%), 414.91 (1.1%)

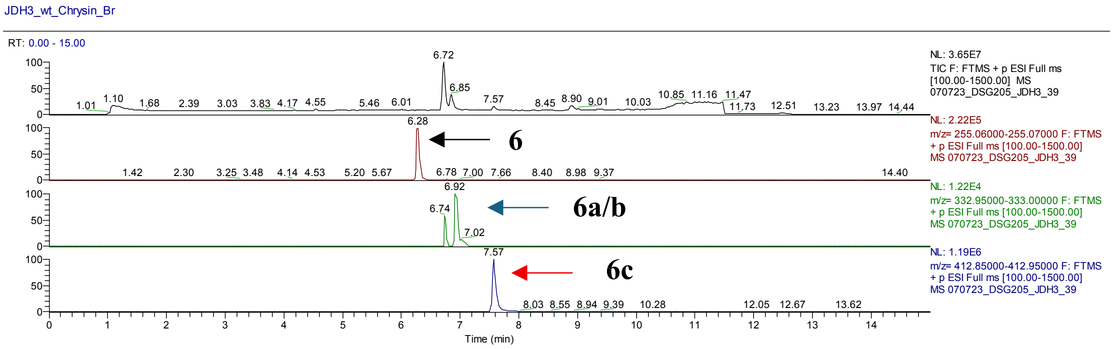

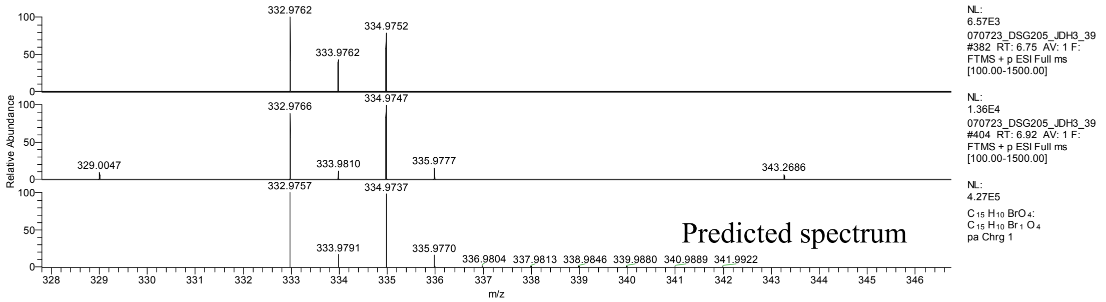

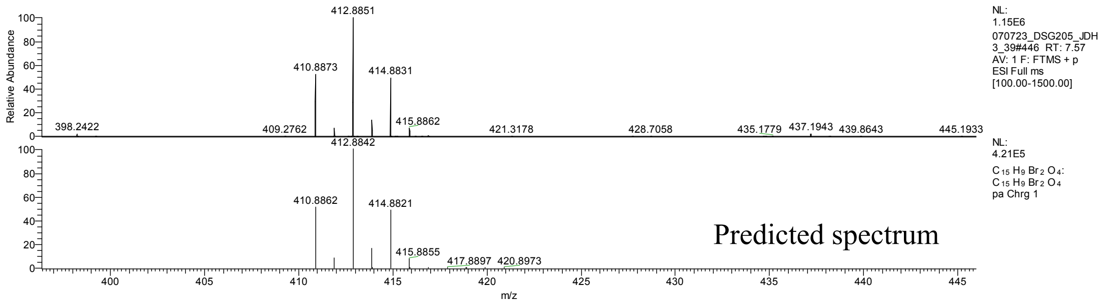

Figure S36. LC-MS analysis of substrate **6** bromination. Mono-brominated product and di-brominated product were observed on EIC chromatogram. Only di-brominated product was isolated from the biotransformation and fully characterised as **6c**. Regiochemistry was determined by NMR. Mass spectra for the mono-brominated product **6a**/**6b**, di-brominated product **6c**, and the corresponding predicted spectrum comparison are shown at the bottom panel.

Compound	$[M+H]^+$ calculated
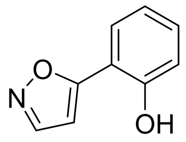	Chemical Formula: $C_9H_8NO_2^+$<br>Exact Mass: 162.0550<br>Molecular Weight: 162.1675<br>m/z: 162.0550 (100.0%), 163.0584 (9.7%)
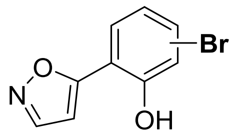	Chemical Formula: $C_9H_7BrNO_2^+$<br>Exact Mass: 239.97<br>Molecular Weight: 241.06<br>m/z: 239.97 (100.0%), 241.96 (97.3%), 240.97 (9.9%), 242.97 (9.7%)
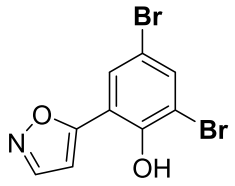	Chemical Formula: $C_9H_6Br_2NO_2^+$<br>Exact Mass: 317.88<br>Molecular Weight: 319.96<br>m/z: 319.87 (100.0%), 317.88 (51.4%), 321.87 (48.7%), 320.88 (9.9%), 318.88 (5.1%), 322.88 (4.9%)

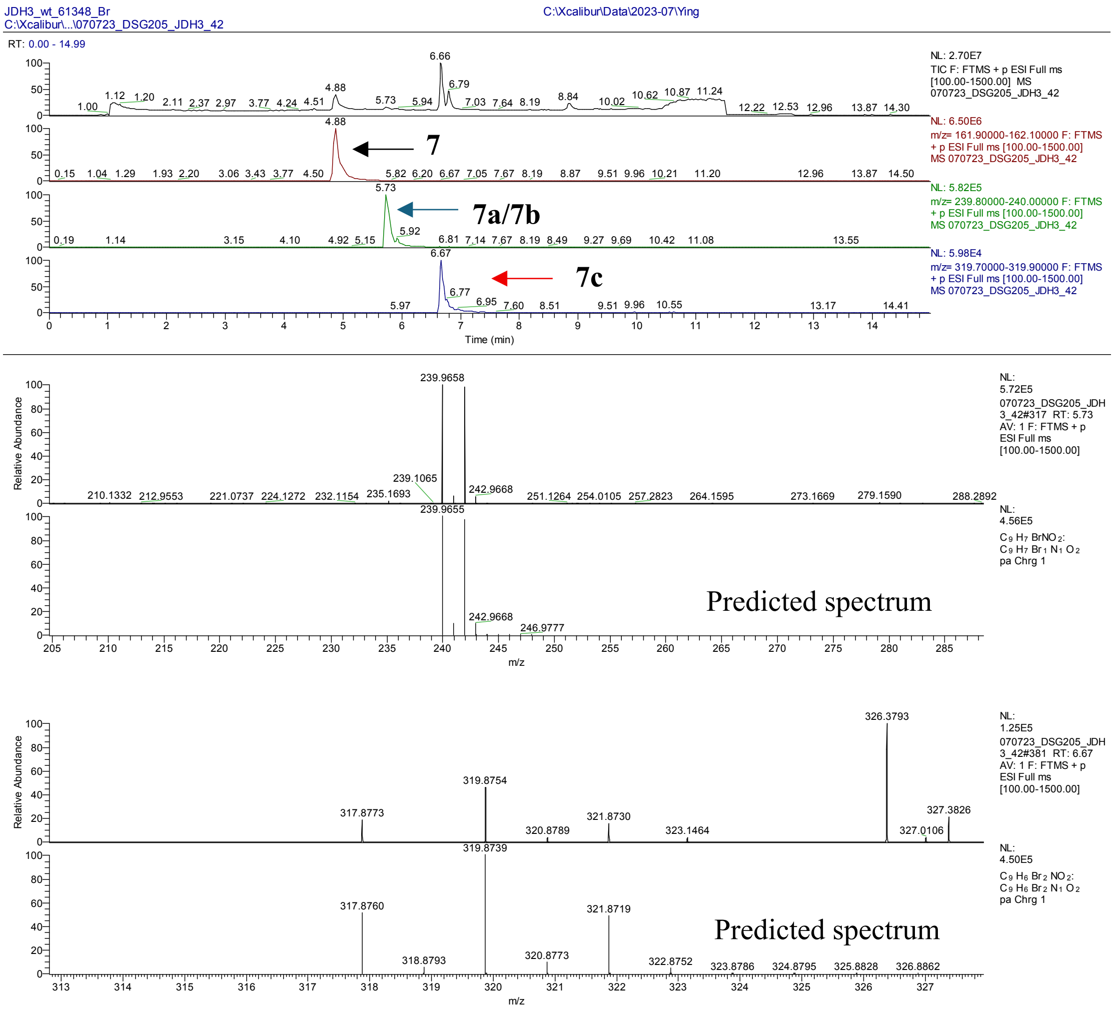

Figure S37. LC-MS analysis of substrate **7** bromination. Mono-brominated product and di-brominated product were observed on EIC chromatogram. Two mono-brominated and one di-brominated products were isolated from the biotransformation and fully characterised as **7a** at 2-position, **7b** at 4-posistion, and **7c** at 2 and 4 position. Regiochemistry was determined by NMR. Mass spectra for the mono-brominated product **7a** and **7b**, di-brominated product **7c**, and the corresponding predicted spectrum comparison are shown at the bottom panel.

3.7 MCD Assays

MCD assay has been widely employed to determine haloperoxidase activity[16, 17]. In addition, MCD can be used as a marker to detect HOX leakage from FDHs[17]. Sometimes, HOX leakage can be promoted in the presence of a particular substrate. We analysed our bromination reactions (7 substrates) and chlorination reactions (substrate **2**) as well as our enzyme in the absence of substrate, demonstrating there is no significant MCD consumption and no HOX leakage. The results show no MCD depletion/halo product formation, during enzymatic reactions with various substrates, indicating that no HOX leakage occurs.

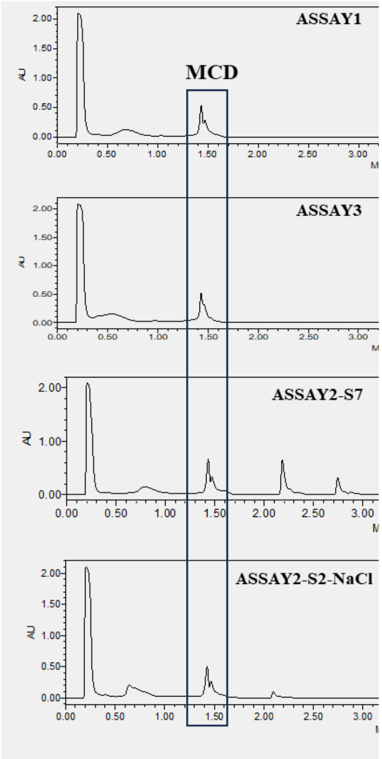

Figure S38. UPLC chromatograms of selected MCD assays. MCD peaks are highlighted by a black box.

3.8 Chemical Bromination Using NBS or NaOBr

The chemical UPLC data is presented in Table S7. Reactions generating new peaks (distinct from enzymatic halogenation products) are highlighted and the corresponding UPLC chromatograms are shown in Figure S39-S41.

Table S7. Summary of UPLC analysis for chemical bromination using NBS or NaOBr.

**Substrate**	**Brominating agent (equiv.)**	**UPLC conversion (%)**	**Product peak retention time matched with enzymatic reaction product**
**1**	NaOBr (1 equiv.)	32%	Yes
	NaOBr (5 equiv.)	38%	Yes
	NBS (1 equiv.)	80%	Yes
	NBS (5 equiv.)	63%	Yes
**2**	NaOBr (1 equiv.)	15%	Yes
	NaOBr (5 equiv.)	89% (major)<br>11% (minor)	No<br>Yes
	NBS (1 equiv.)	38%	Yes
	NBS (5 equiv.)	100%	No
**3**	NaOBr (1 equiv.)	1%	Yes
	NaOBr (5 equiv.)	0%	-
	NBS (1 equiv.)	7%	Yes
	NBS (5 equiv.)	6%	Yes
**4**	NaOBr (1 equiv.)	0%	-
	NaOBr (5 equiv.)	2%	No
	NBS (1 equiv.)	5%	Yes
	NBS (5 equiv.)	3%	Yes
**5**	NaOBr (1 equiv.)	0%	-
	NaOBr (5 equiv.)	4%	No
	NBS (1 equiv.)	14%	Yes
	NBS (5 equiv.)	100%	No
**6**	NaOBr (1 equiv.)	0%	-
	NaOBr (5 equiv.)	0%	-
	NBS (1 equiv.)	3%	Yes (di-Br **6c**)
	NBS (5 equiv.)	73%	No (mixture of mono-Br **6a/6b**)

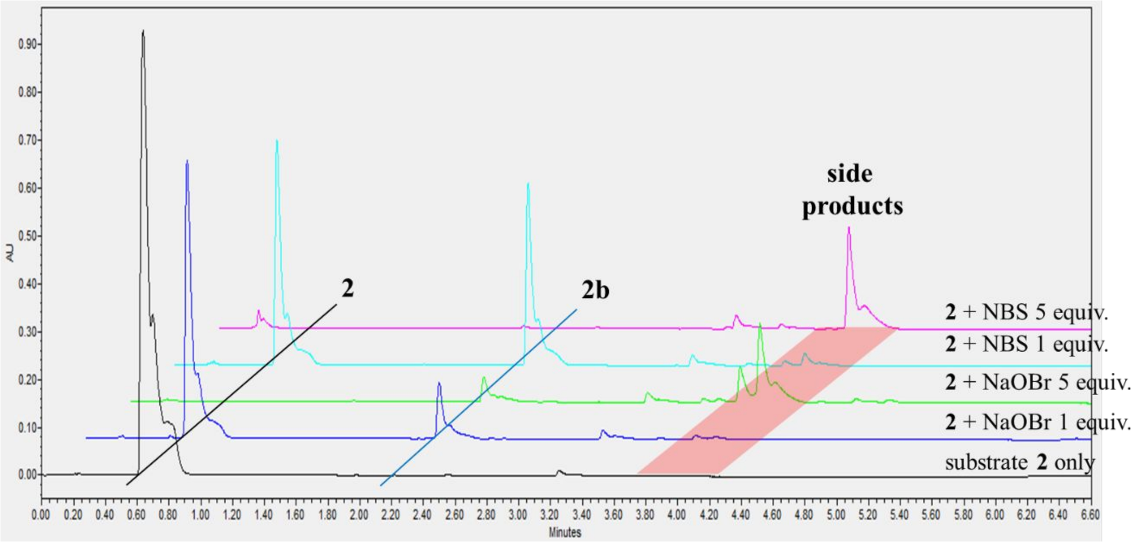

Figure S39. UPLC chromatograms of chemical bromination using NBS or NaOBr. The black line corresponds to substrate **2**, the blue line represents product **2b**, the pink area indicates side products (new peaks not matching with what from enzymatic reactions). Assay conditions are listed at the right hand side.

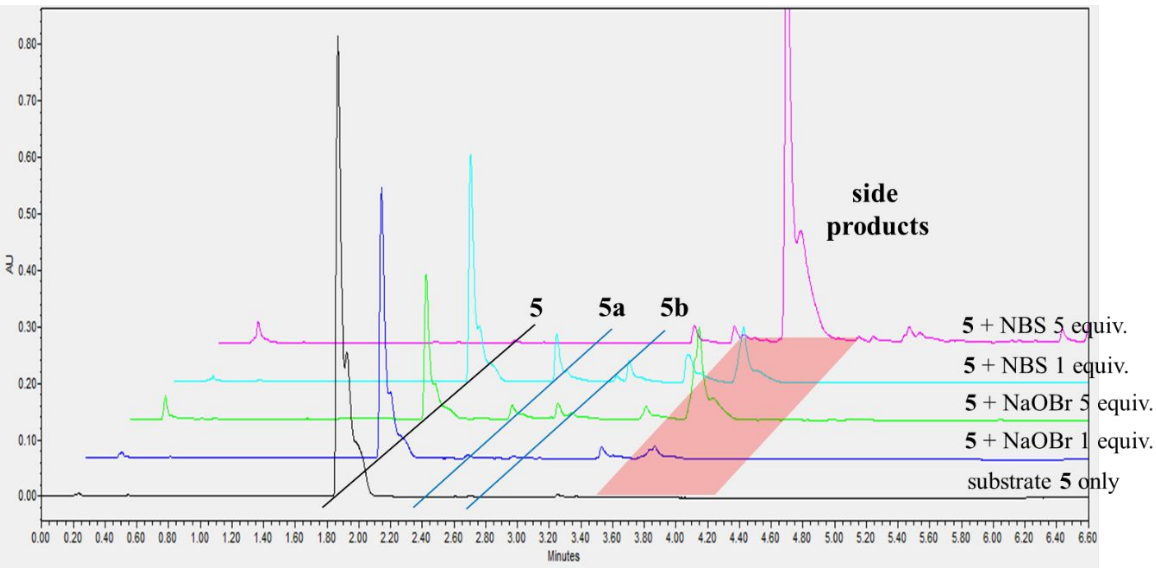

Figure S40. UPLC chromatograms of chemical bromination using NBS or NaOBr. The black line corresponds to substrate **5**, the blue lines represents products **5a** and **5b**, the pink area indicates side products (new peaks not matching with what from enzymatic reactions). Assay conditions are listed at the right hand side.

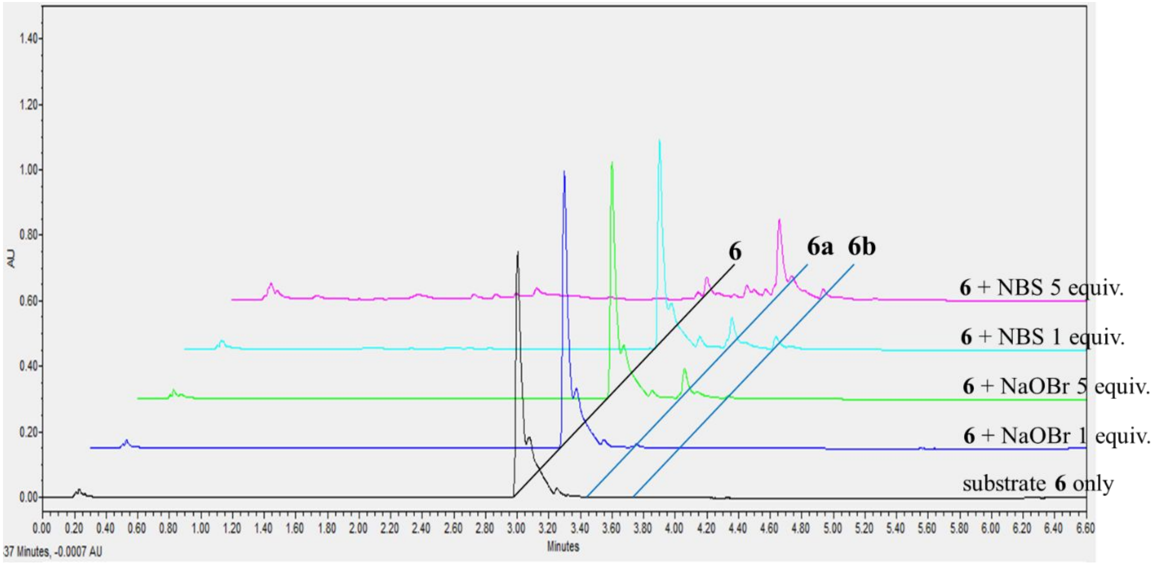

Figure S41. UPLC chromatograms of chemical bromination using NBS or NaOBr. The black line corresponds to substrate **6**, the blue lines represents products **6a** and **6b**. Assay conditions are listed at the right hand side.

3.9 Preliminary Assay Optimisation

To determine optimal conditions for the halogenase assay using CHEESY1, a selection of buffers and flavin reductases was tested using two different flavin reductases, in two buffers, with or without catalase and SOD, using compound **1** as the substrate, as **1** gave a stable and good UPLC conversion. Assays of each combination were incubated overnight and checked on UPLC. Data demonstrating the conversion of substrate **1** are shown in Table S8.

Table S8. Conversion on UPLC (measured by peak area at λmax 254 nm) of a set of CHEESY1 activity tests on substrate **1** using PrnF and Fre as the flavin reductase, with or without catalase and SOD in solution, either in HEPES buffer or in phosphate buffer at pH 8.0.

		UPLC conversions (%)			
		PrnF		Fre	
		HEPES buffer	Phosphate buffer	HEPES buffer	Phosphate buffer
Control	(-) Catalase & SOD	/	/	/	/
	(+) Catalase & SOD	/	/	/	/
10 μM CHEESY1	(-) Catalase & SOD	14	3	17	4
	(+) Catalase & SOD	20	4	21	5

50 μM CHEESY1	(-) Catalase & SOD	64	28	63	30
	(+) Catalase & SOD	83	37	65	37

3.10 Kinetics of CHEESY1

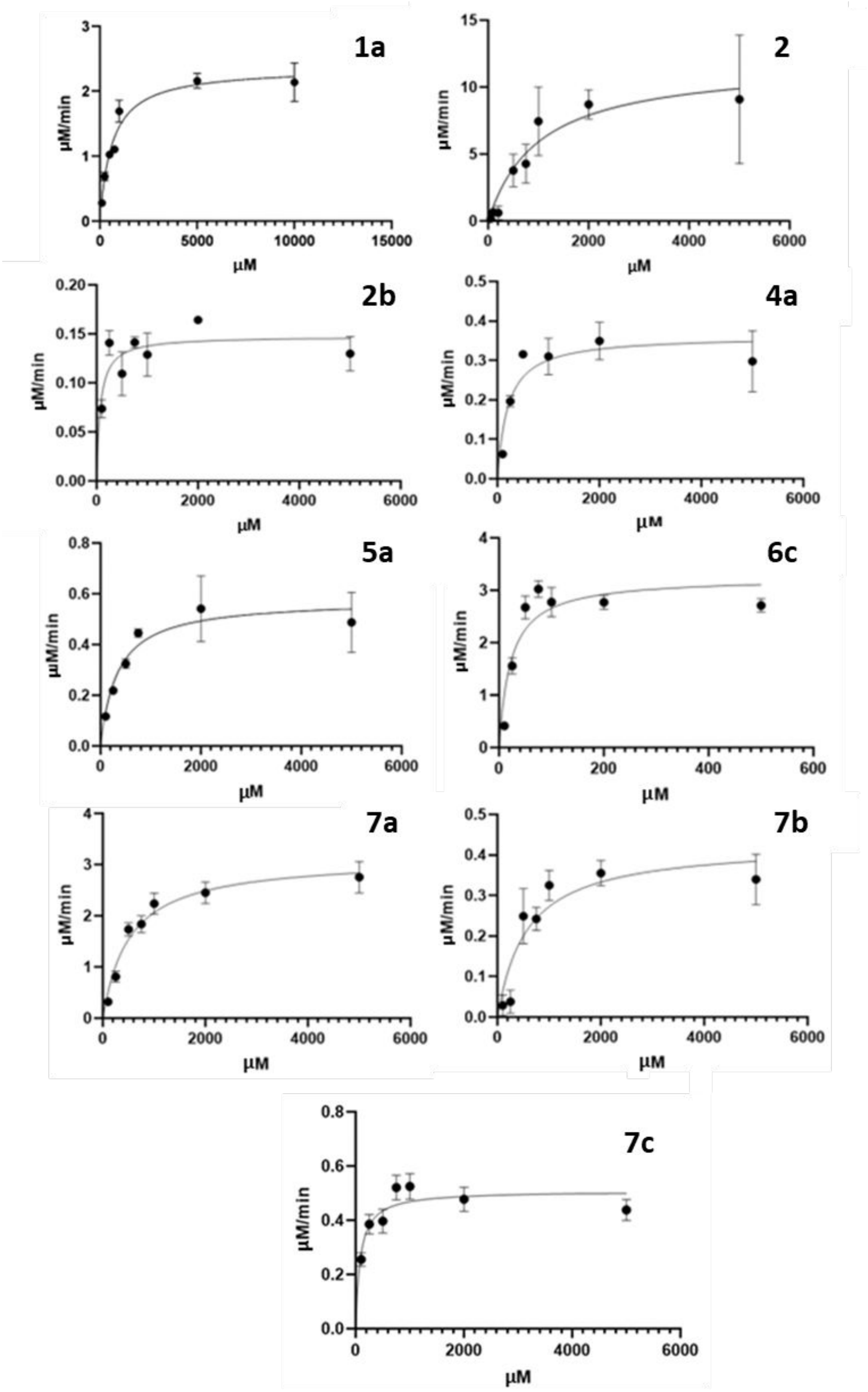

Figure S42. Michaelis–Menten (MM) kinetics of CHEESY1 with substrate **1** to **7** except **3**. Kinetics was measured by product formation, with the exception of **2** chlorination measured by substrate consumption. Each data point is a mean of triplicate measurements.

3.11 Product Characterisation and NMR Spectra

5-Bromo-6-hydroxyquinoline (1a)

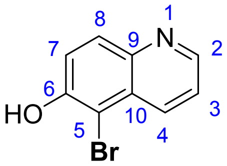

A substrate input (20 mg of **1**) was used in a total reaction volume containing 1-3 mM substrate concentration. Extraction (3 times using ethyl acetate) and purification (normal phase, 100 % n-Hexane to 100 % Dichloromethane gradient 20-30 column volumes as required) were conducted. The resulting dried product (3.1 mg, 10 %) was dissolved in CD_3OD and characterised using ^1H NMR and ^{13}C NMR.

^1H NMR (500 MHz, CD_3OD) δ 8.67 (td, *J* = 4.4, 1.3 Hz, 1H, H-2), 8.55 (dt, *J* = 9.2, 1.9, 1H, H-4), 7.89 (d, *J* = 9.1 Hz, 1H, H-7), 7.56 (ddd, *J* = 8.6, 4.4, 1.6 Hz, 1H, H-3), 7.47 (dd, *J* = 9.2, 0.9 Hz, 1H, H-8) ppm.
^{13}C NMR (126 MHz, CD_3OD) δ 154.7 (C-6), 148.5 (C-2), 144.5 (C-9), 135.8 (C-4), 130.3 (C-10), 129.9 (C-7), 123.6 (C-3), 122.9 (C-8), 105.0 (C-5) ppm.
HRMS (ESI$^+$) *m/z* C_9H_7BrNO $[M+H]^+$, found 223.9711, expected 223.9706 (for ^{79}Br).
These data are consistent with the literature characterisation for this compound[18].

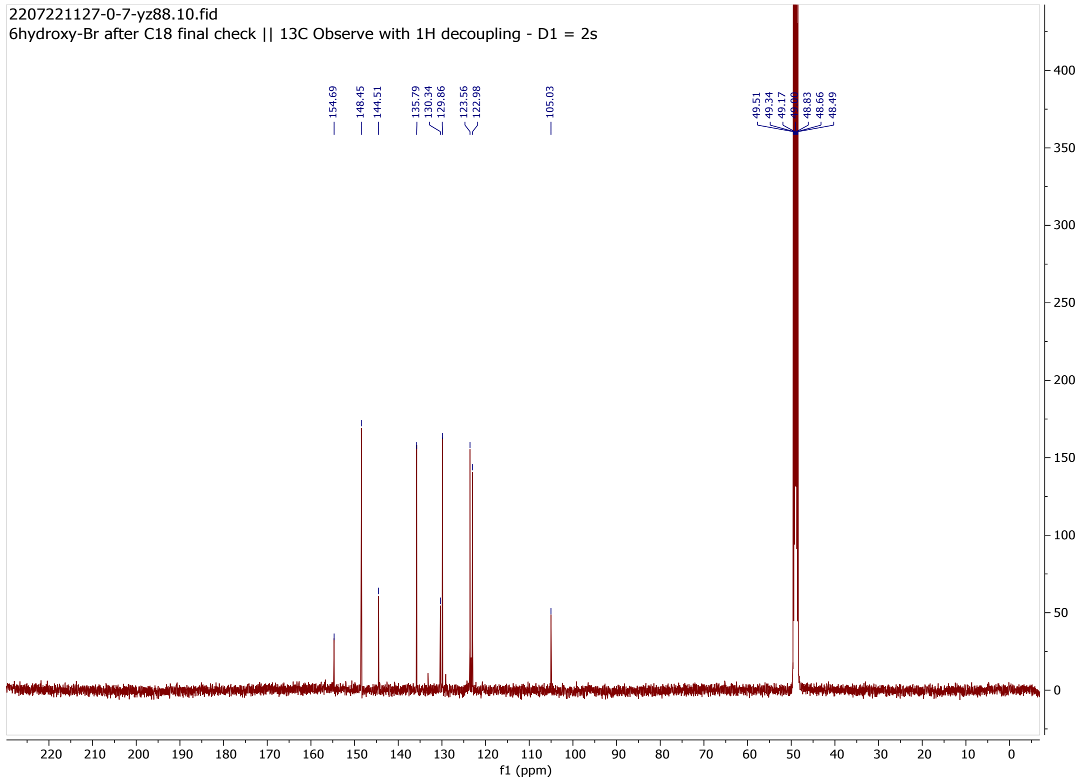
2207221127-0-7-yz88.10.fid
6hydroxy-Br after C18 final check || 13C Observe with 1H decoupling - D1 = 2s
154.69
148.45
144.51
135.79
130.34
129.86
123.56
122.98
105.03
49.51
49.34
49.17
48.83
48.66
48.49
f1 (ppm)

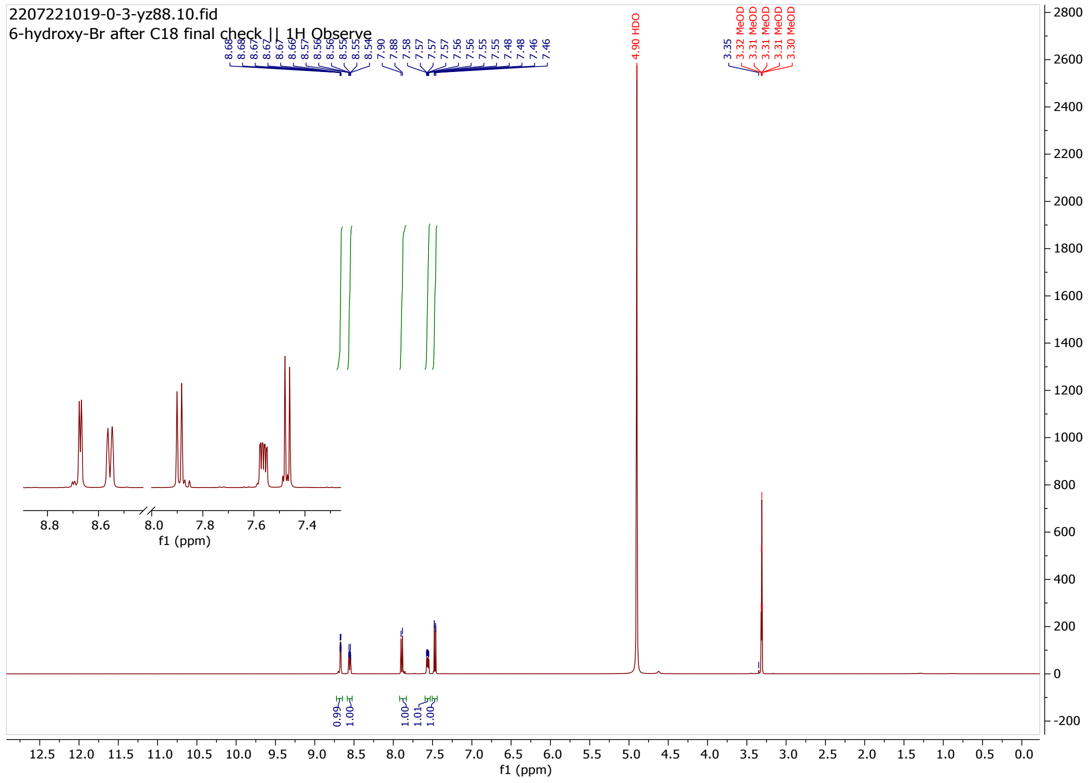

Figure S43. ^{1}H NMR (500 MHz, CD_3OD) and ^{13}C NMR (126 MHz, CD_3OD) of 5-bromo-6-hydroxyquinoline (**1a**).

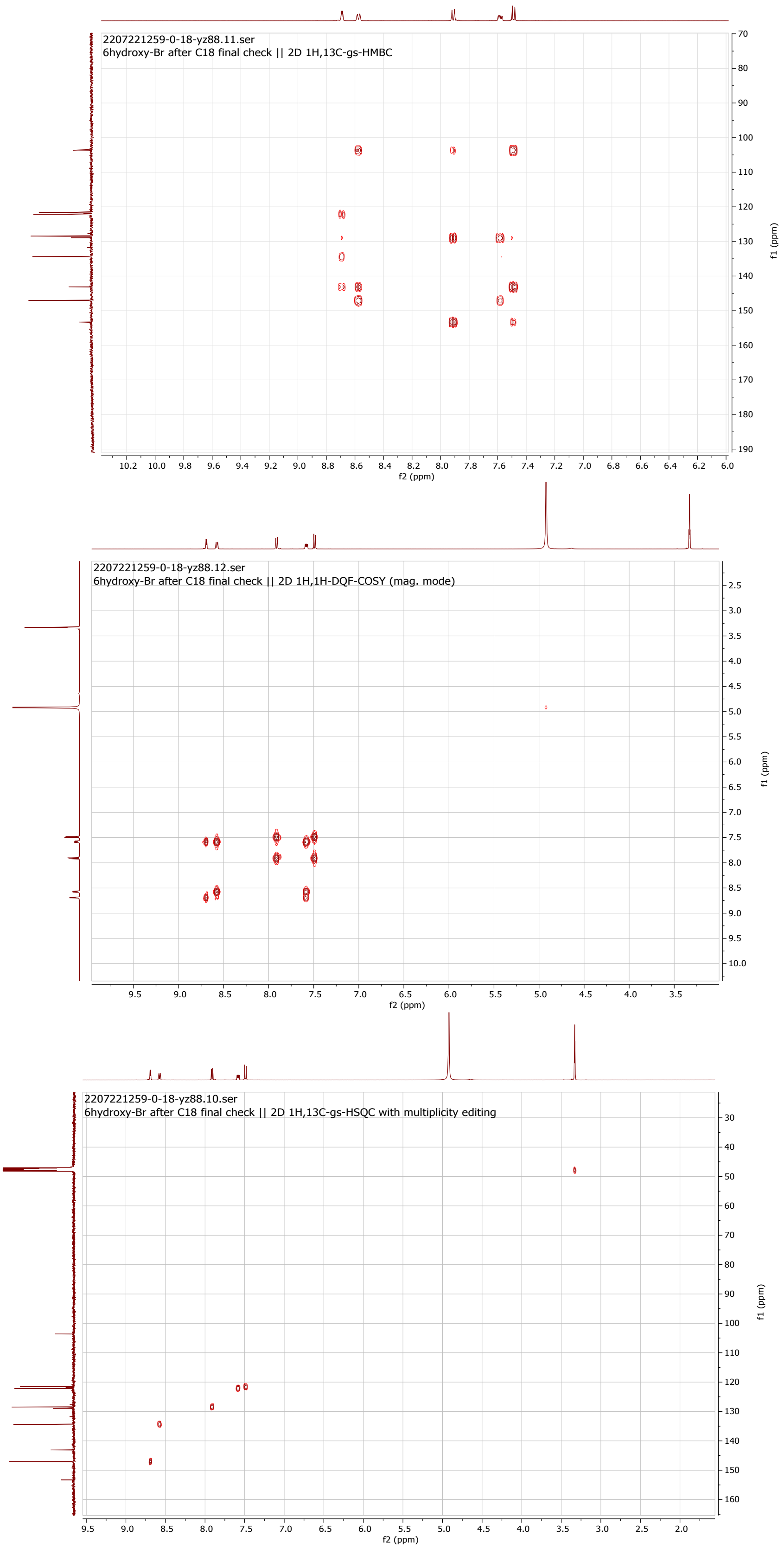

Figure S44. 2-D NMR (500 MHz, CD_3OD), HMBC, COSY and HSQC and spectra of 5-bromo-6-hydroxyquinoline (**1a**).

4-Chloro-3-methyl-1-phenyl-1H-pyrazol-5-amine (2a)

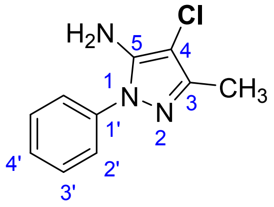

A substrate input (15 mg of **2**) was used in a total reaction volume of 150 mL. Extraction (3 times using ethyl acetate) and purification (normal phase, 100 % n-Hexane to 100 % Dichloromethane gradient 20-30 column volumes as required) were conducted. The resulting dried product (2.0 mg, 11 % yield) was dissolved in CD_3OD and characterised using ^1H NMR and ^{13}C NMR.

^1H NMR (500 MHz, CD_3OD) δ 7.57 – 7.50 (m, 4H, H-2', H-3'), 7.47 – 7.38 (m, 1H, H-4'), 2.19 (s, 3H, CH_3) ppm.

^{13}C NMR (126 MHz, CD_3OD) δ 147.1 (C-3), 144.6 (C-5), 139.9 (C-1'), 130.6 (C-2'), 128.8 (C-4'), 125.1 (C-3'), 92.8 (C-4'), 11.4 (CH_3) ppm.

HRMS (ESI^+) *m/z* chemical formula: $C_{10}H_{10}ClN_3$; found 208.0632 $[M+H]^+$, expected 208.0635 (for ^{35}Cl).

These data are consistent with the literature characterisation for this compound[19].

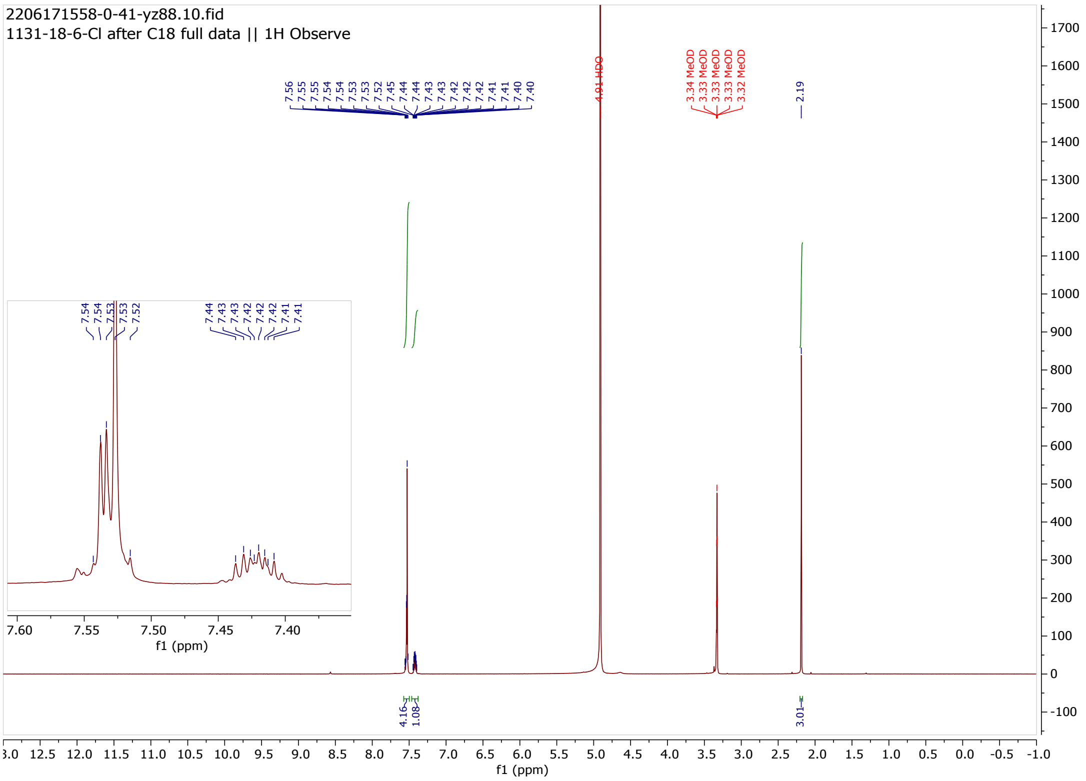

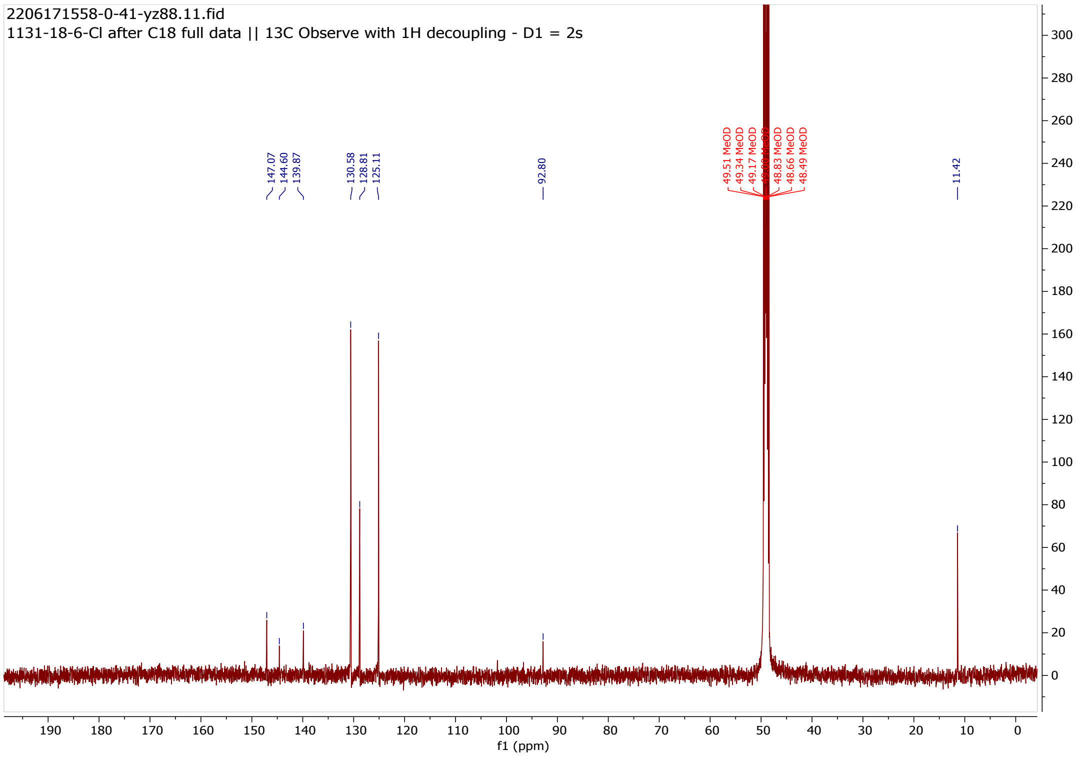

Figure S45. ^{1}H NMR (500 MHz, CD_3OD) and ^{13}C NMR (126 MHz, CD_3OD) of 4-chloro-3-methyl-1-phenyl-1H-pyrazol-5-amine (**2a**).

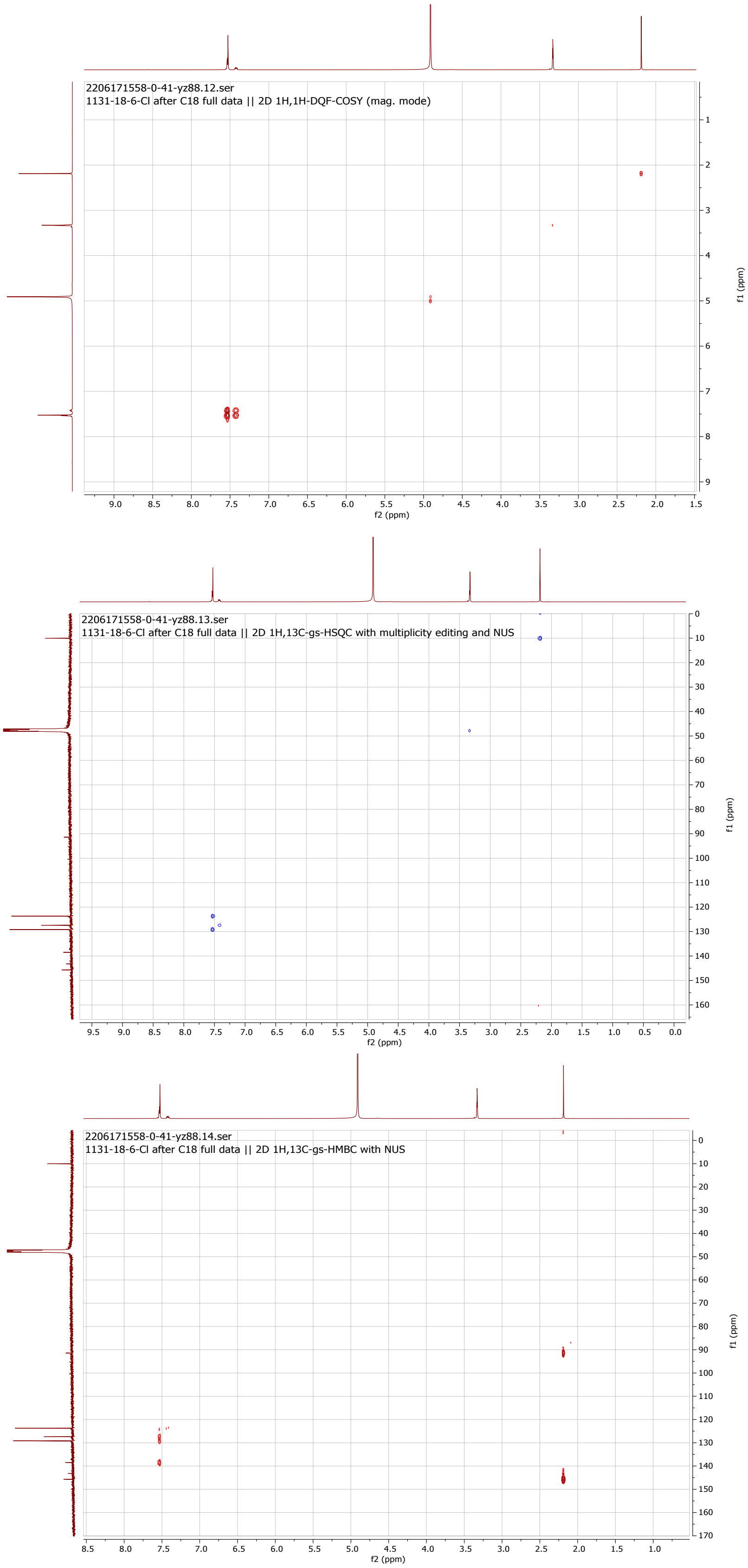

Figure S46. 2-D NMR (500 MHz, CD_3OD), COSY, HSQC and HMBC spectra of of 4-chloro-3-methyl-1-phenyl-1H-pyrazol-5-amine (**2**

4-Bromo-3-methyl-1-phenyl-1H-pyrazol-5-amine (2b)

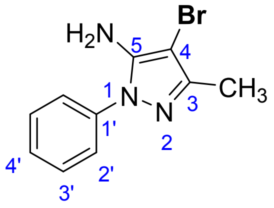

A substrate input of 17 mg of **2** was used, resulting in a total volume containing 1-3 mM substrate concentration. Extraction (3 times using ethyl acetate) and purification (normal phase, 100 % n-Hexane to 100 % Dichloromethane gradient 20-30 column volumes as required) were conducted. The resulting dried product (4.2 mg, 19.2 % yield) was characterised using ^{1}H NMR and ^{13}C NMR.

^{1}H NMR (500 MHz, CD_3OD) δ 7.57 – 7.50 (m, 4H, H-2', H-3'), 7.40 – 7.37 (m, 1H, H-4'), 2.19 (s, 3H, CH_3) ppm.

^{13}C NMR (126 MHz, $CDCl_3$) δ 147.4 (C-3), 142.7 (C-5), 138.9 (C-1'), 129.7 (C-2'), 127.6 (C-4'), 123.6 (C-3'), 78.8 (C-4), 12.7 (CH_3) ppm.

HRMS (ESI$^+$) *m/z* chemical formula: $C_{10}H_{10}BrN_3$; found 252.0146 $[M+H]^+$, expected 252.0131 (for ^{79}Br).

These data are consistent with the literature characterisation for this compound[19].

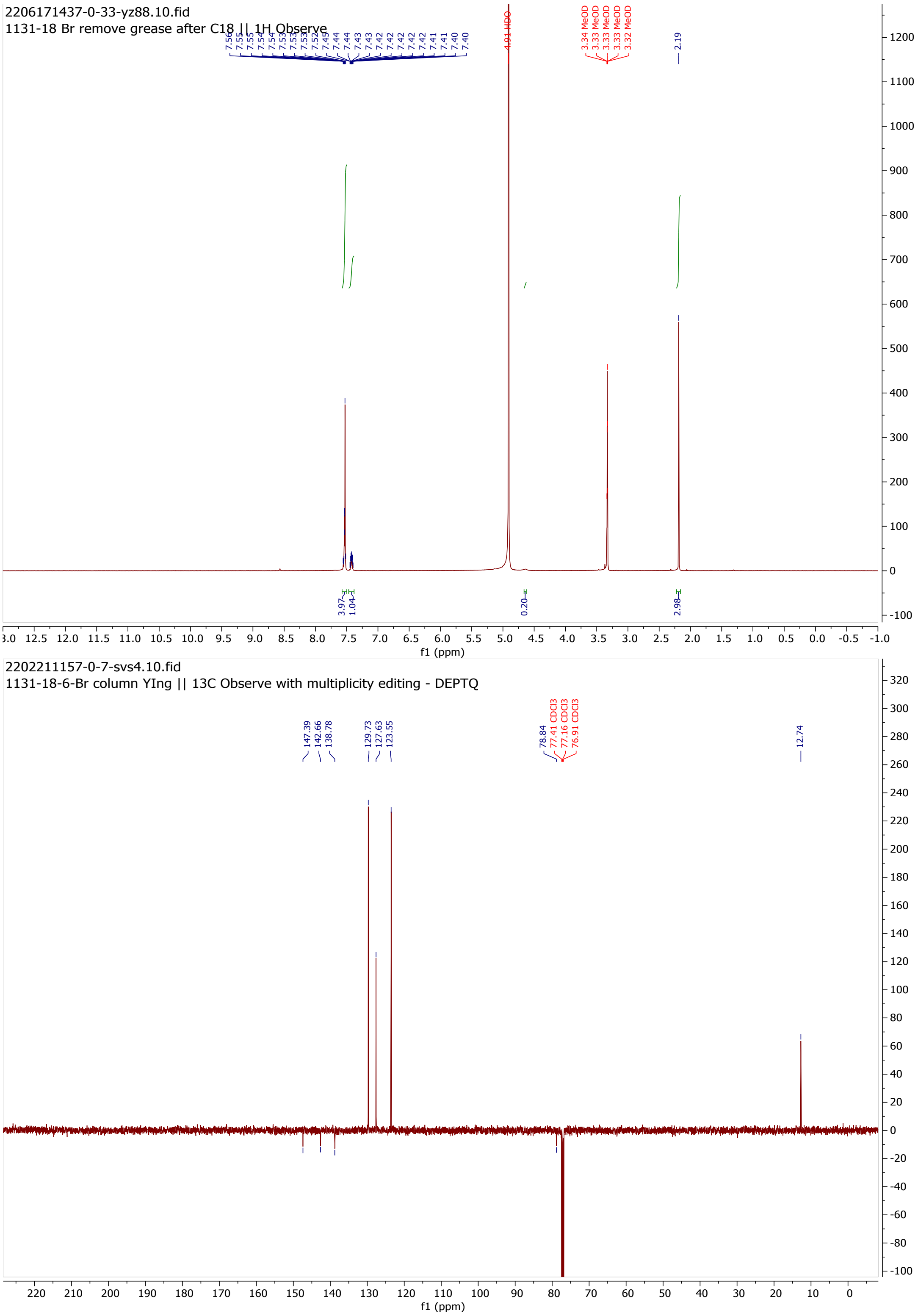

Figure S47. ^{1}H NMR (500 MHz, CD_3OD) and ^{13}C NMR (126 MHz, $CDCl_3$) of 4-bromo-3-methyl-1-phenyl-1H-pyrazol-5-amine (**2b**).

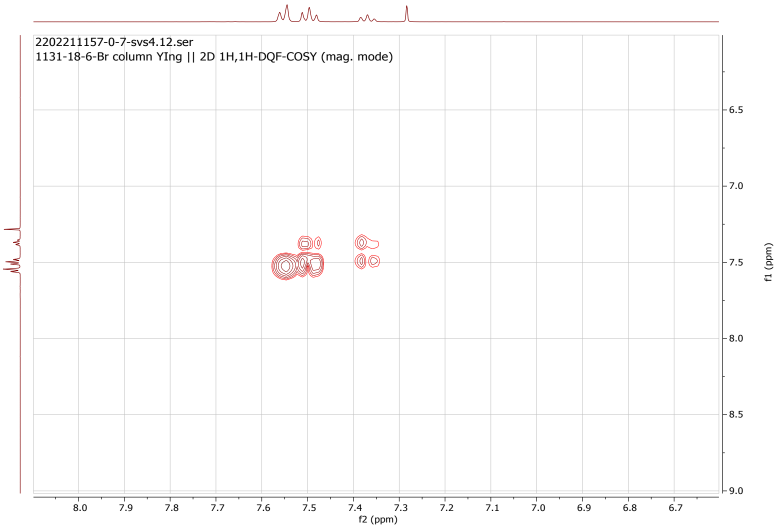

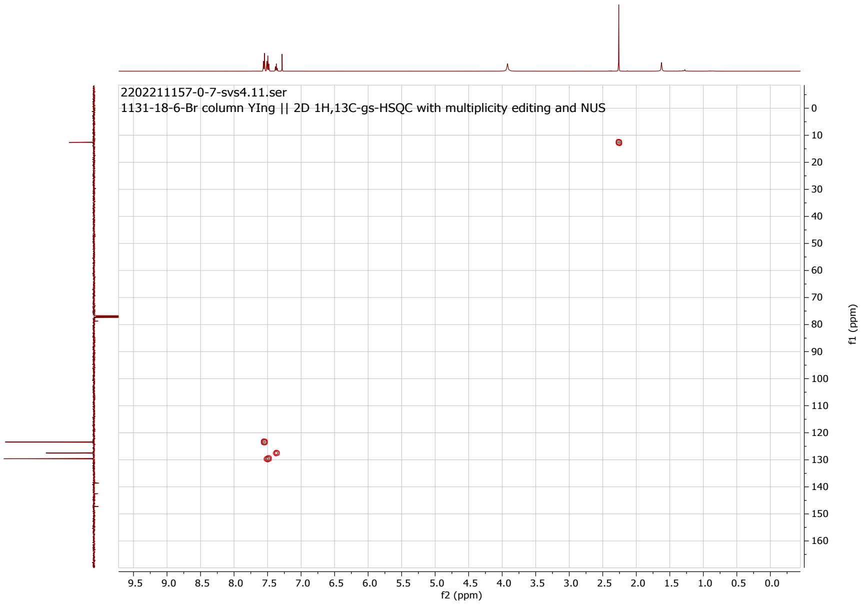

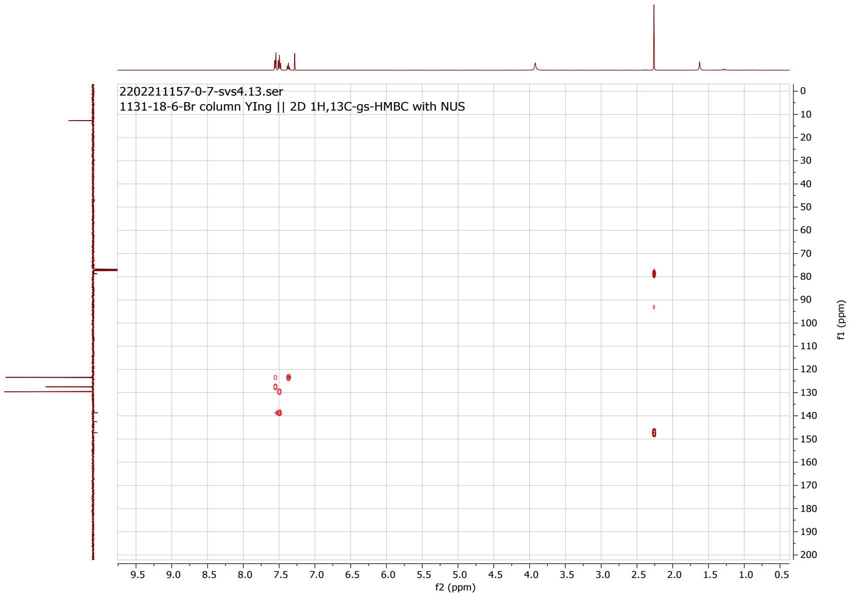

Figure S48. 2-D NMR (500 MHz, $CDCl_3$), COSY, HSQC and HMBC spectra of of 4-bromo-3-methyl-1-phenyl-1H-pyrazol-5-amine (**2b**).

2-(2’-Anilino)-3-bromo-1H-indole (3a)

A substrate input of 11 mg of **3** was used, resulting in a total reaction volume containing 1-3 mM substrate concentration. The resulting dried product (1.6 mg, 14 % yield) was dissolved in CD_3OD and characterised using ^1H NMR and ^{13}C NMR.

^1H NMR (500 MHz, CD_3OD) δ 7.49 (d, *J* = 7.8 Hz, 1H, H-4), 7.41 (d, *J* = 8.1 Hz, 1H, H-7), 7.27 (d, *J* = 7.7 Hz, 1H, H-6’), 7.23 – 7.18 (m, 2H, H-6, H-4’), 7.14 (t, *J* = 7.5 Hz, 1H, H-5), 6.89 (d, *J* = 8.1 Hz, 1H, H-3’), 6.80 (t, *J* = 7.5 Hz, 1H, H-5’) ppm.

^{13}C NMR (126 MHz, CD_3OD) δ 147.1 (C-2’), 137.3 (C-7a), 135.2 (C-2), 132.3 (C-6’), 130.9 (C-4’), 129.0 (C-3a), 123.6 (C-6), 121.0 (C-5), 119.3 (C-4), 118.7 (C-5’), 118.3 (C-1’), 117.1 (C-3’), 112.5 (C-7), 90.6 (C-3) ppm.

HRMS (ESI^+) *m/z* chemical formula: $C_{14}H_{11}BrN_2$; found 287.0184 $[M+H]^+$, expected 287.0178 (for ^{79}Br).

These data are consistent with the literature characterisation for this compound[20].

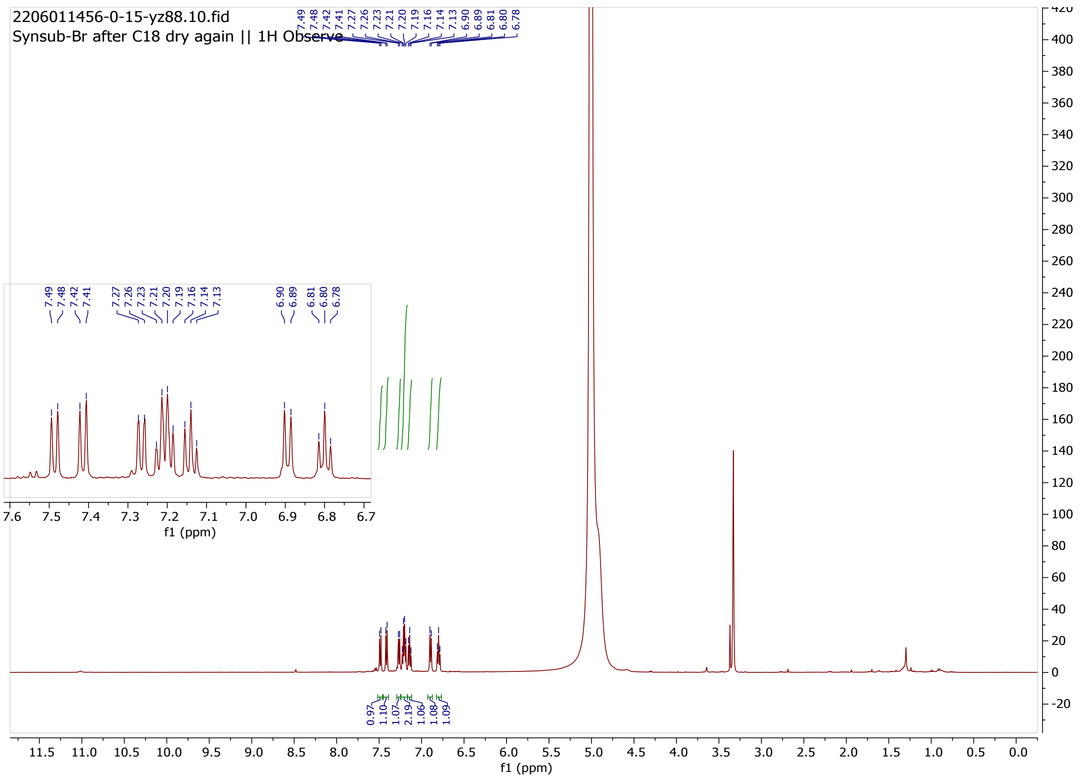

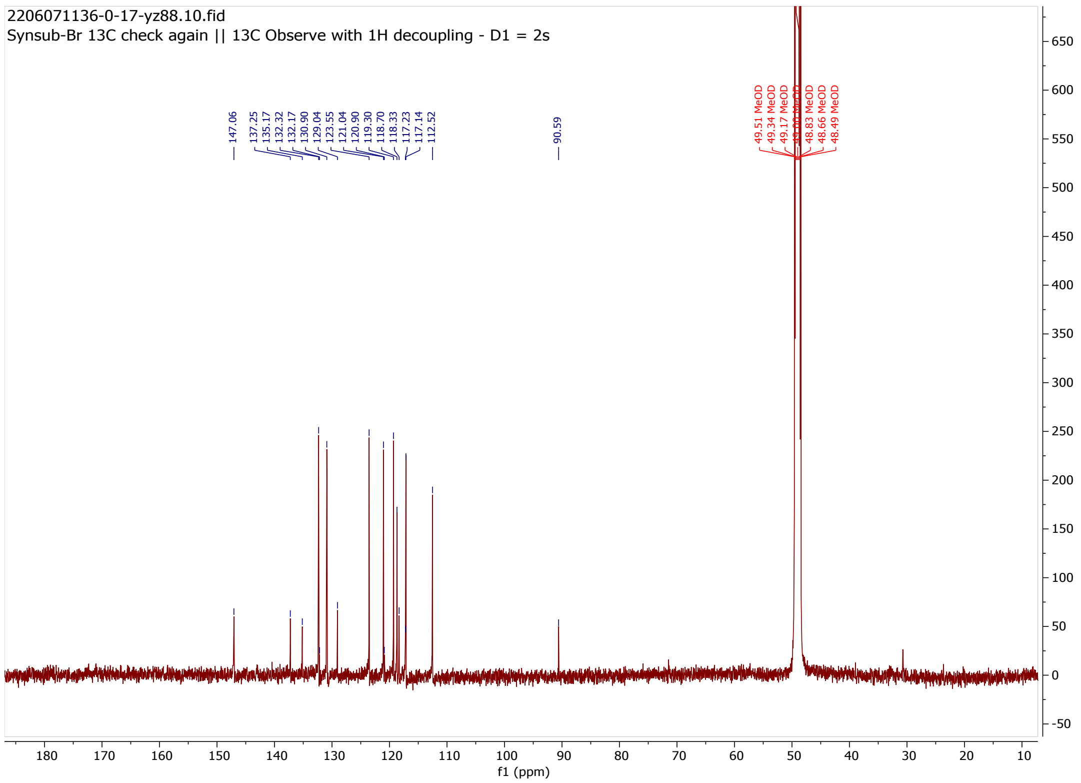

Figure S49. ^{1}H NMR (500 MHz, CD_3OD) and ^{13}C NMR (126 MHz, CD_3OD) of 2-(3-bromo-1H-indol-2-yl)aniline (**3a**).

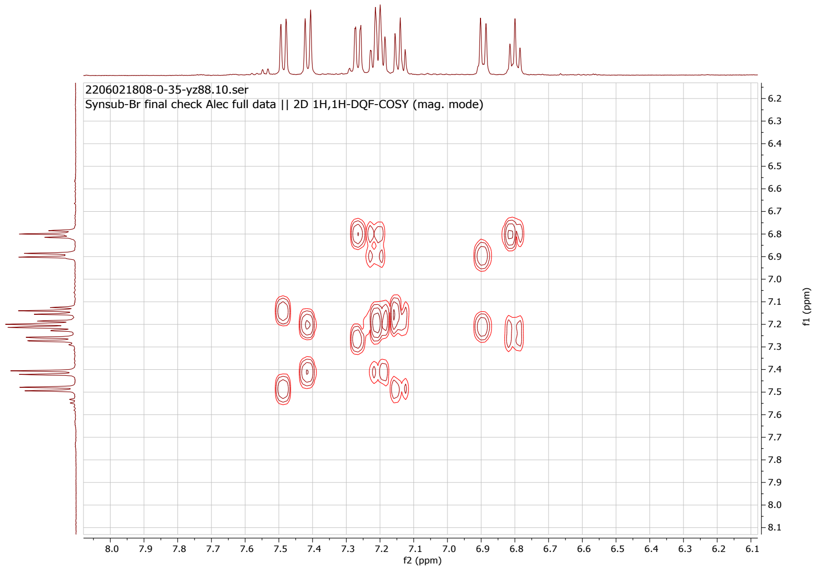

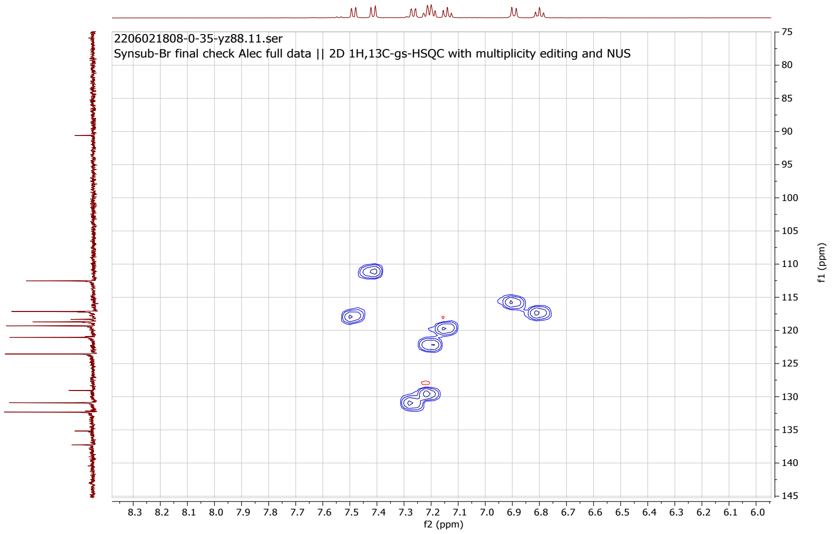

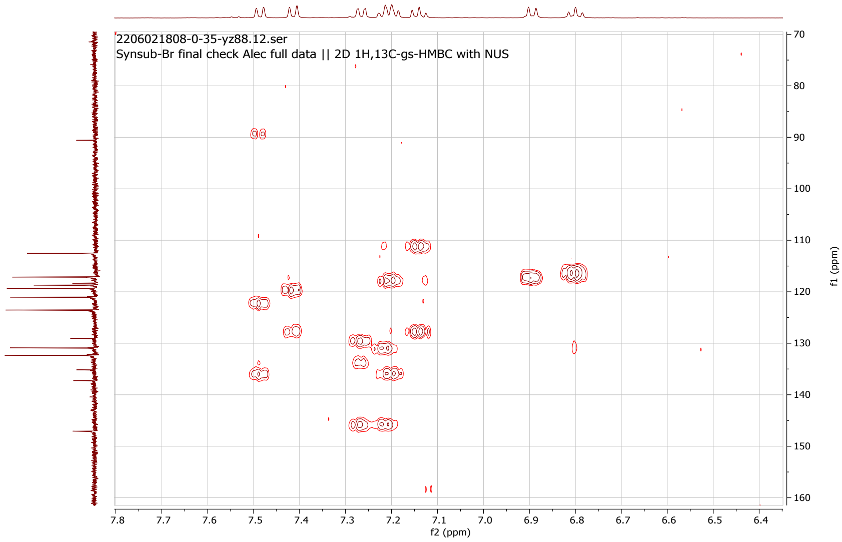

Figure S50. 2-D NMR (500 MHz, CD_3OD), COSY, HSQC and HMBC spectra of of 2-(3-bromo-1*H*-indol-2-yl)aniline (**3a**).

7-Bromo-2-methylquinolin-8-ol (4a)

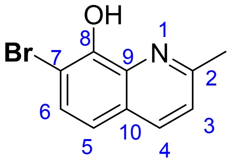

The substrate input of 16 mg of **4** was used, resulting in a total reaction volume containing 1-3 mM substrate concentration. The resulting dried product (2.0 mg, 8.4 % yield) was dissolved in CD_3OD and characterised using ^{1}H NMR and ^{13}C NMR.

^{1}H NMR (500 MHz CD_3OD) δ 8.15 (d, J = 8.4 Hz, 1H, H-4), 7.56 (d, J = 8.8 Hz, 1H, H-6), 7.44 (d, J = 8.4 Hz, 1H, H-3), 7.27 (d, J = 8.8 Hz, 1H, H-5), 2.75 (s, 3H, CH_3) ppm.

^{13}C NMR (126 MHz, CD_3OD) δ 158.1 (C-2), 149.7(C-8), 138.0 (C-10), 136.2 (C-4), 129.8 (C-6), 126.0 (C-9), 122.6 (C-3), 118.1 (C-5), 104.4 (C-7), 23.5 (CH_3) ppm.

HRMS (ESI$^+$) *m/z* chemical formula: $C_{10}H_8BrNO$; found 239.9869 $[M+H]^+$, expected 237.9862 (for ^{79}Br).

These data are consistent with the literature characterisation for this compound[21].

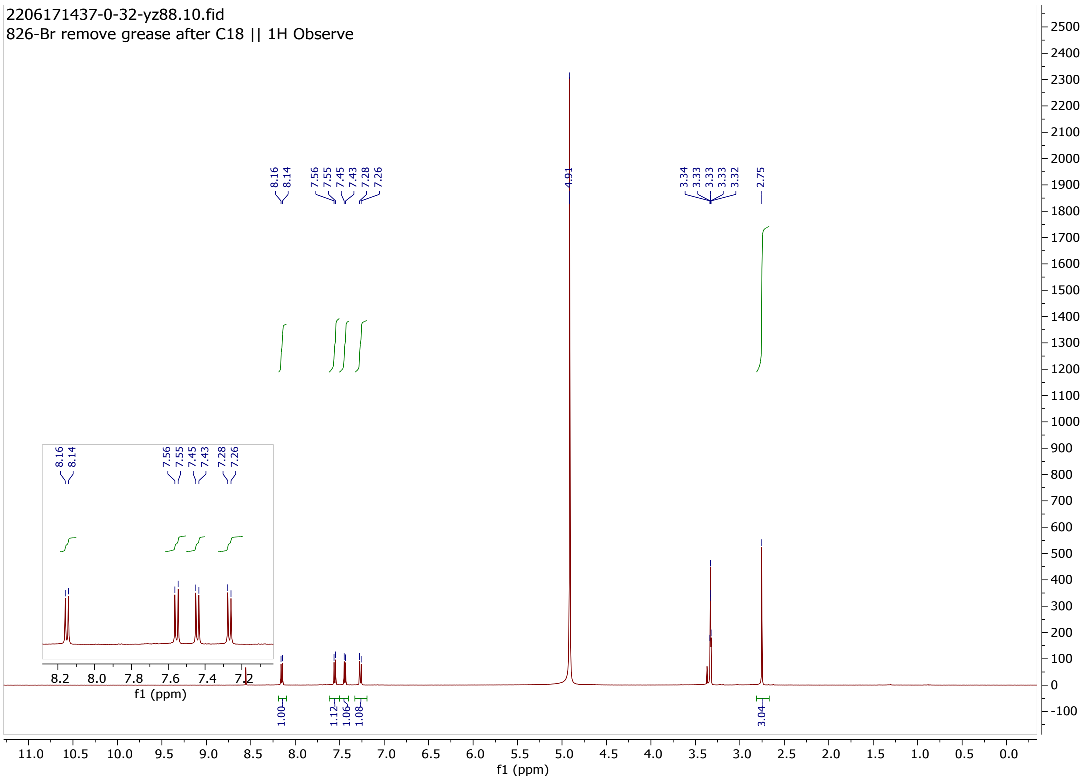

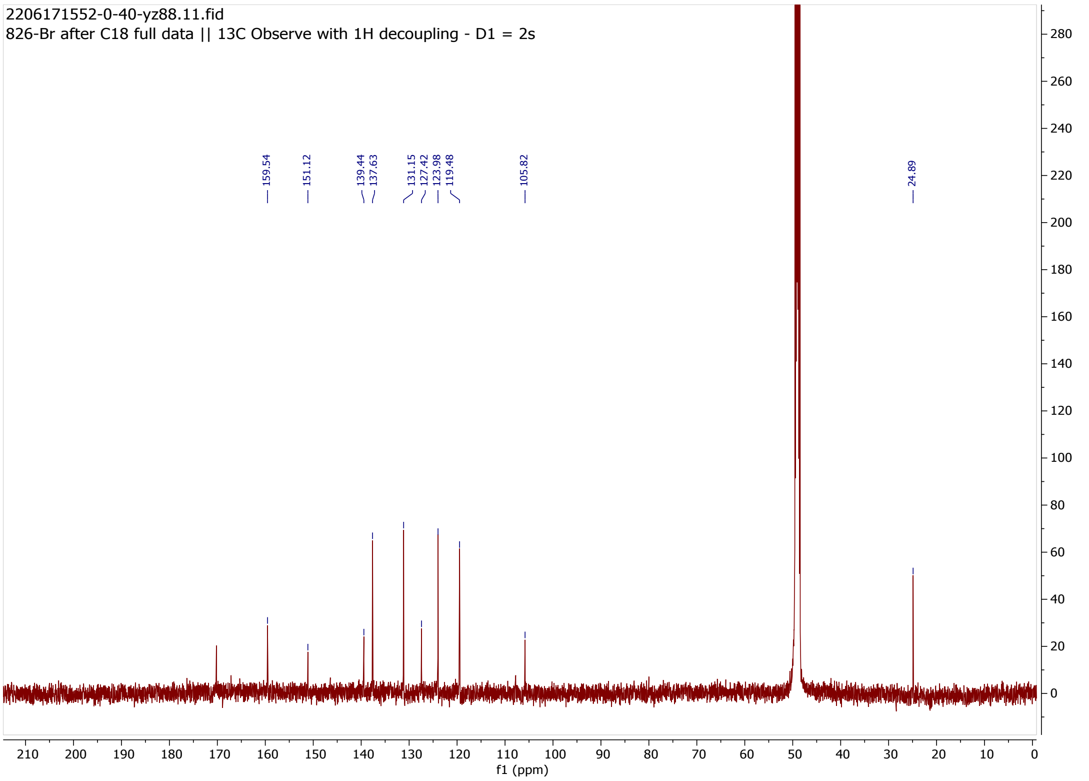

Figure S51. ^{1}H NMR (500 MHz, CD_3OD) and ^{13}C NMR (126 MHz, CD_3OD) of 7-bromo-2-methylquinolin-8-ol (**4a**).

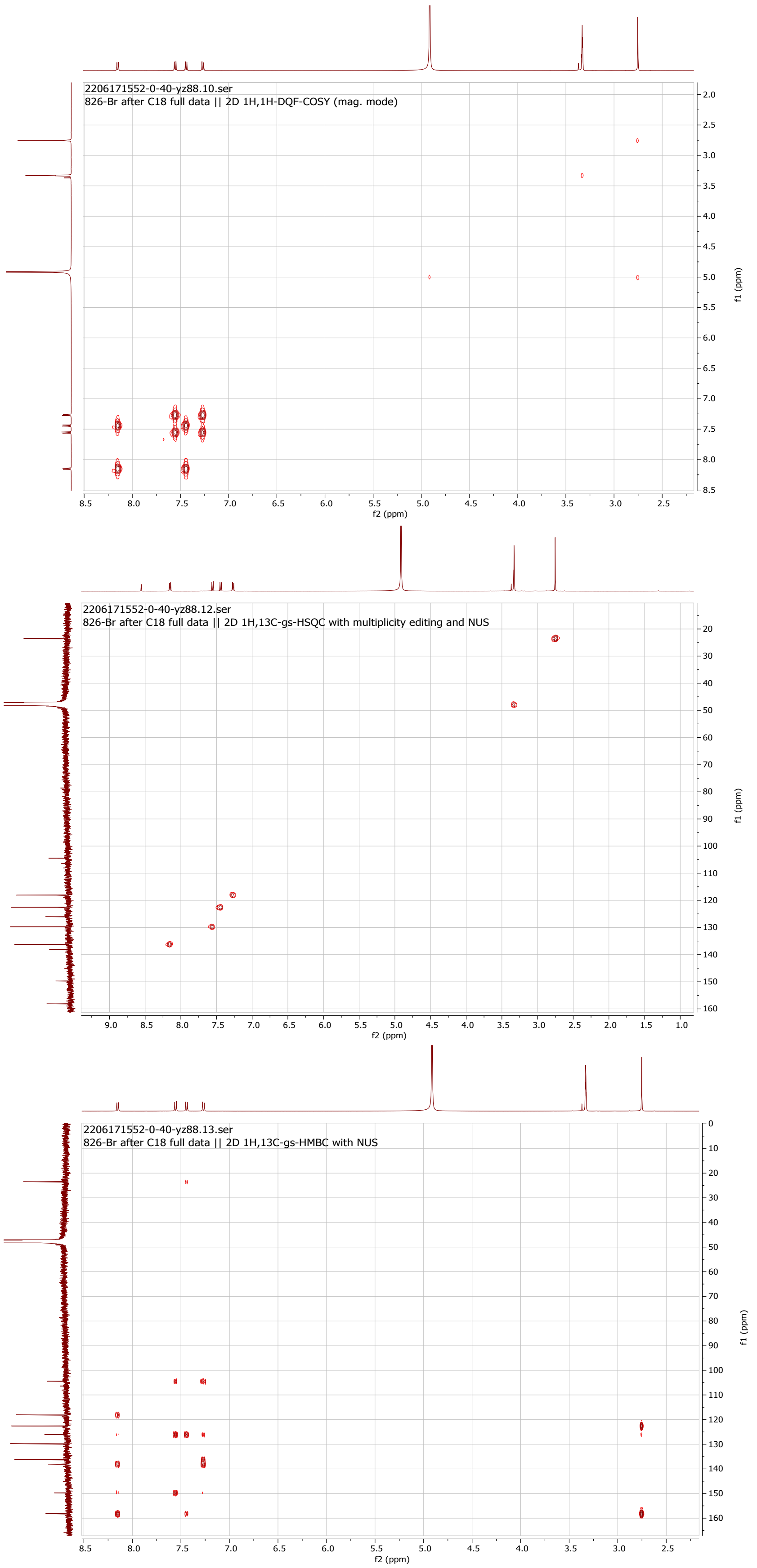

Figure S52. 2-D NMR (500 MHz, CD_3OD), COSY, HSQC and HMBC spectra of of 7-bromo-2-methylquinolin-8-ol (**4a**).

(3-Bromo-4-hydroxyphenyl)(4'-hydroxyphenyl)methanone (5a)

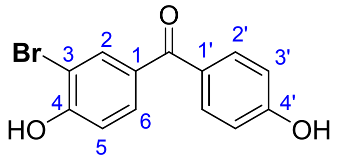

A substrate input of 15 mg of **5** was used, resulting in a total reaction volume containing 1-3 mM substrate concentration. The resulting dried product (1.9 mg, 9 % yield) was dissolved in CD_3OD and characterised using ^{1}H NMR and ^{13}C NMR.

^{1}H NMR (500 MHz, CD_3OD) δ 7.91 (d, J = 2.1 Hz, 1H, H-2), 7.71 – 7.65 (m, 2H, H-2'), 7.62 (dd, J = 8.4, 2.1 Hz, 1H, H-6), 6.99 (d, J = 8.3 Hz, 1H, H-5), 6.93 – 6.88 (m, 2H, H-3') ppm.

^{13}C NMR (126 MHz, CD_3OD) δ 195.5 (C=O), 163.4 (C-4'), 160.0 (C-4), 136.4 (C-2), 133.6 (C-2'), 132.3 (C-6), 131.8 (C-1), 130.1 (C-1'), 116.6 (C-5), 116.1 (C-3'), 111.0 (C-Br) ppm.

HRMS (ESI$^+$) *m/z* chemical formula: $C_{13}H_9BrO_3$; found 292.9812 $[M+H]^+$, expected 292.9808 (for ^{79}Br). No literature characterisation for this compound was found.

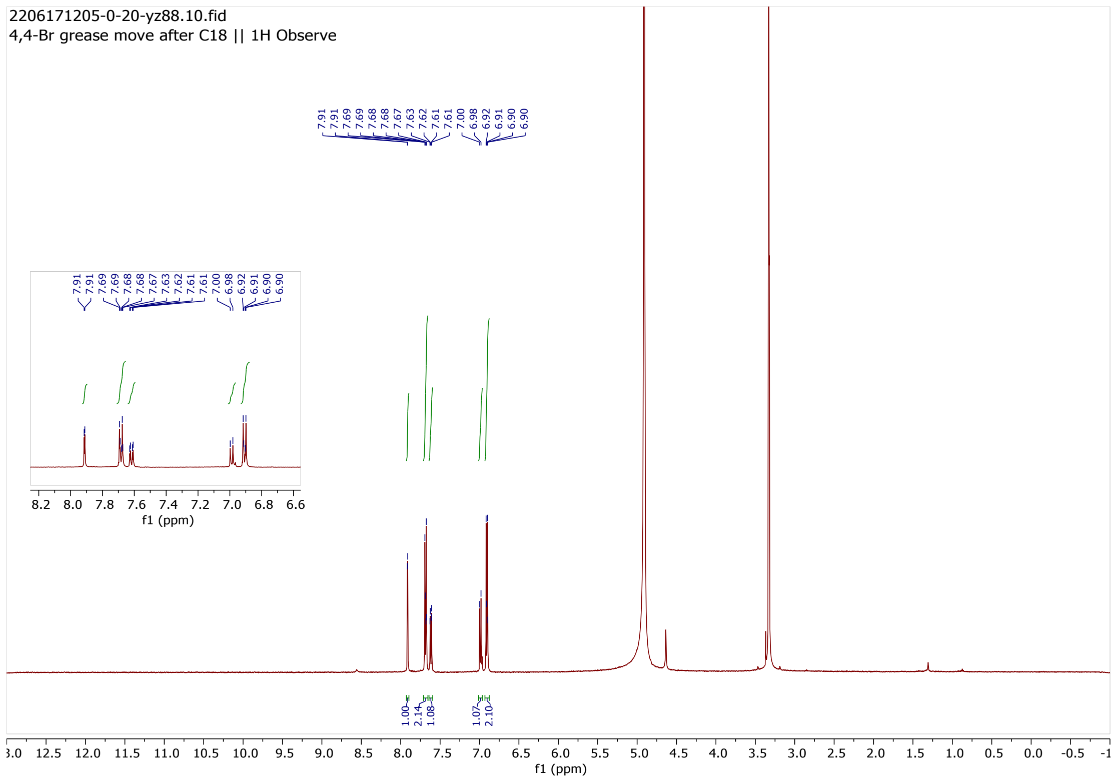

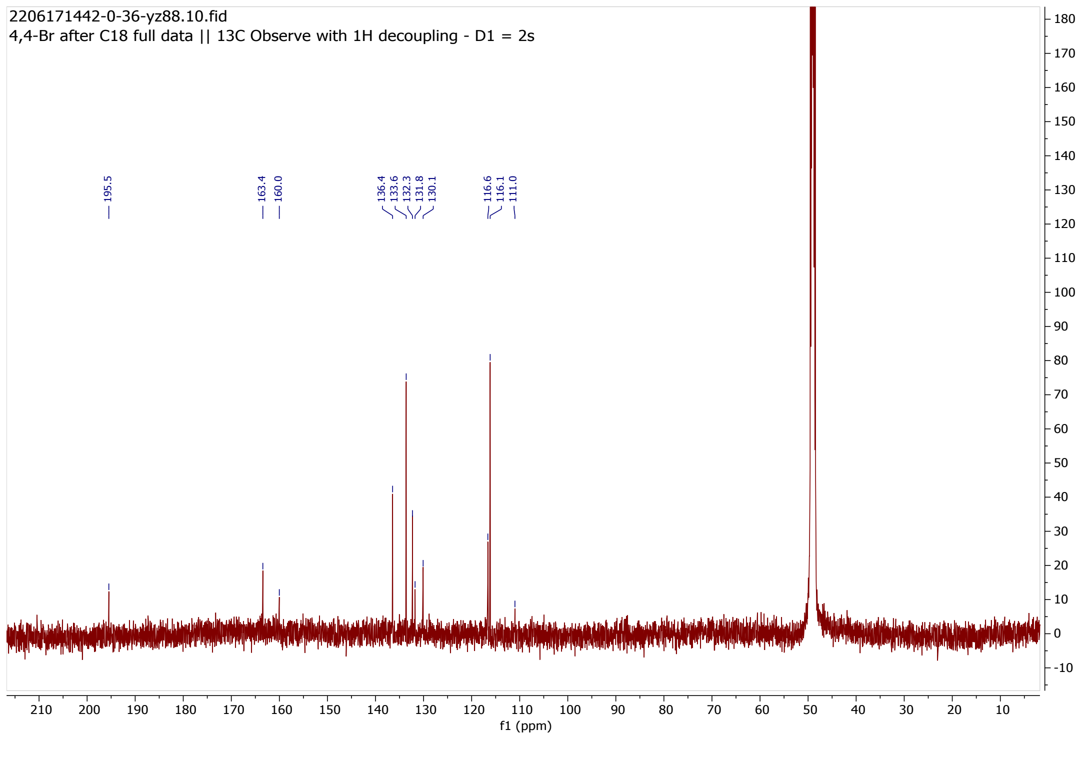

Figure S53. ^{1}H NMR (500 MHz, CD_3OD) and ^{13}C NMR (126 MHz, CD_3OD) of (3-bromo-4-hydroxyphenyl)(4-hydroxyphenyl)methanone (**5a**).

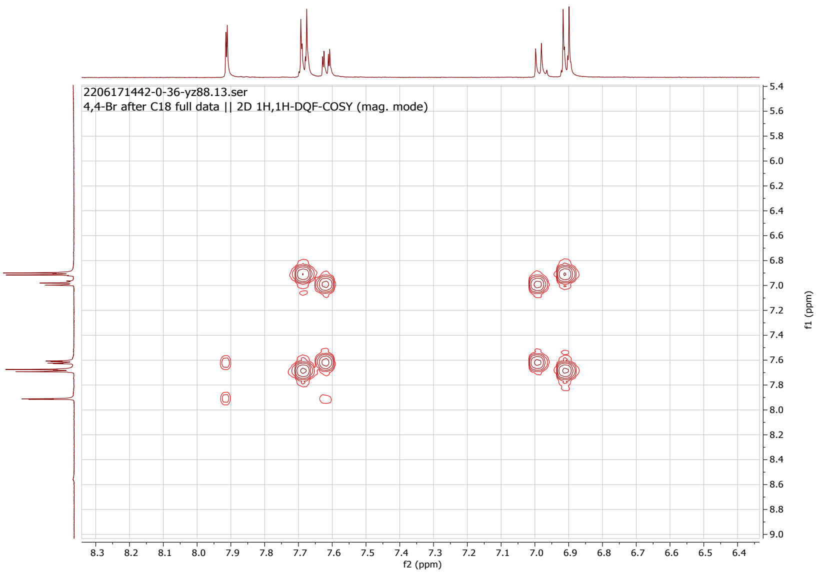

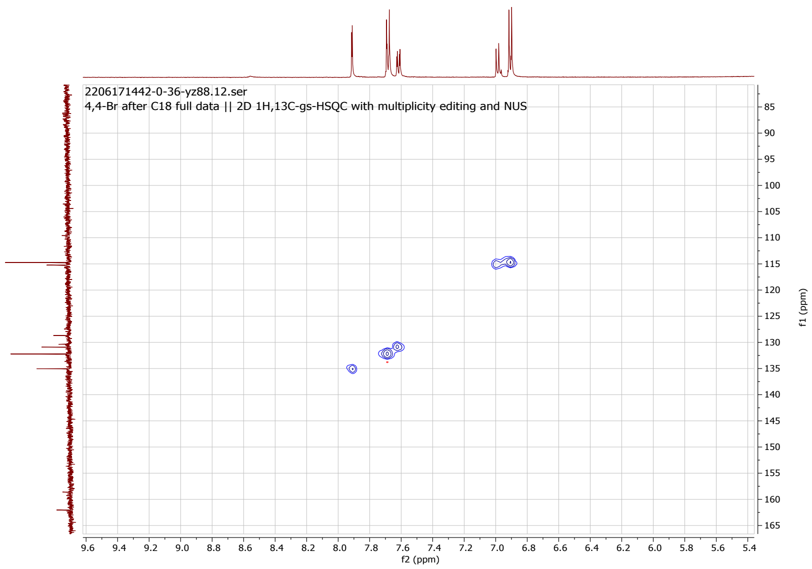

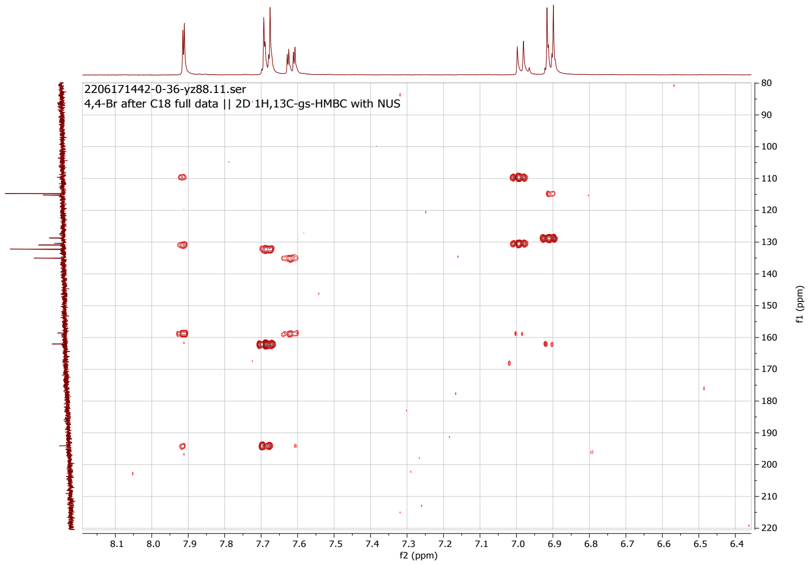

Figure S54. 2-D NMR (500 MHz, CD_3OD), COSY, HSQC and HMBC spectra of (3-bromo-4-hydroxyphenyl)(4-hydroxyphenyl)methanone (**5a**).

Bis(3-bromo-4-hydroxyphenyl)methanone (5b)

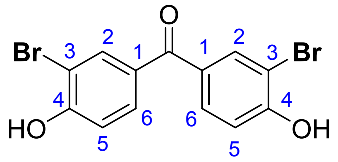

This product was isolated from the identical biotransformation process described in the identification of **5a**. However, it constituted the minor product. Based on a partial NMR analysis of the mixture, the product (isolated product not pure < 1 mg, yield < 5 %) was identified as a symmetrical di-bromo compound (only characterized through ^{1}H NMR).

^{1}H NMR (500 MHz, $CDCl_3$) δ 8.00 (d, J = 2.0 Hz, 2H, H-2), 7.70 (dd, J = 8.5, 2.0 Hz, 2H, H-6), 7.13 (d, J = 8.4 Hz, 2H, H-5) ppm.

HRMS (ESI^+) *m/z* chemical formula: $C_{13}H_8Br_2O_3$; found 370.8918 $[M+H]^+$, expected 370.8913(for ^{79}Br). No literature characterisation for this compound was found.

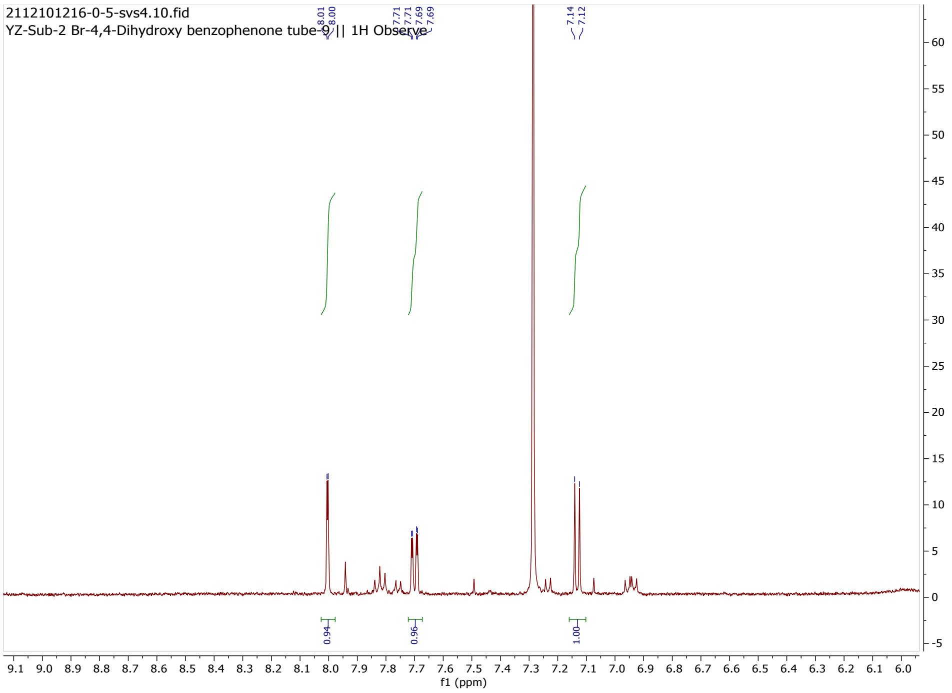

Figure S55. ^{1}H NMR (500 MHz, CD_3OD) of bis(3-bromo-4-hydroxyphenyl)methanone (**5b**).

6,8-Dibromo-5,7-dihydroxy-2-phenyl-4H-chromen-4-one (6c)

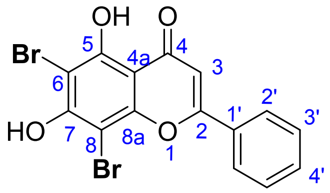

The substrate input of 14 mg of **6** was used, the substrate concentration agreed with 50 µg/mL (due to the solubility issue), resulting in a total reaction volume of 280 mL. Extraction (3 times using ethyl acetate) and purification (normal phase, 100 % Hexane to 99 % EtOAc containing 1 % MeOH 20-30 column volumes as required) were conducted. The resulting dried product (1.6 mg, 7 % yield) was dissolved in DMSO-d_6 and characterised using ^{1}H NMR and ^{13}C NMR (some quaternary ^{13}C signals were not observed).

^{1}H NMR (500 MHz, DMSO-d_6) δ 8.27 – 8.10 (m, 2H, H-2'), 7.68 – 7.61 (m, 3H, H-4' & H-3'), 7.23 (s, 1H, H-3) ppm.
^{13}C NMR (126 MHz, DMSO- d_6) δ 181.6 (C-4), 163.5 (C-2), 157.1 (C-5/C-7), 152.4 (C-8a), 132.6 (C-4'), 130.3 (C-1'), 129.4 (C-3'), 126.6 (C-2'), 105.2 (C-3), 105.0 (C-4a), 88.6 (C-6/C-8) ppm.
HRMS (ESI^{+}) *m/z* chemical formula: $C_{15}H_8Br_2O_4$; found 410.8873 $[M+H]^{+}$, expected 410.8862.
These data are consistent with the literature characterisation for this compound[22].

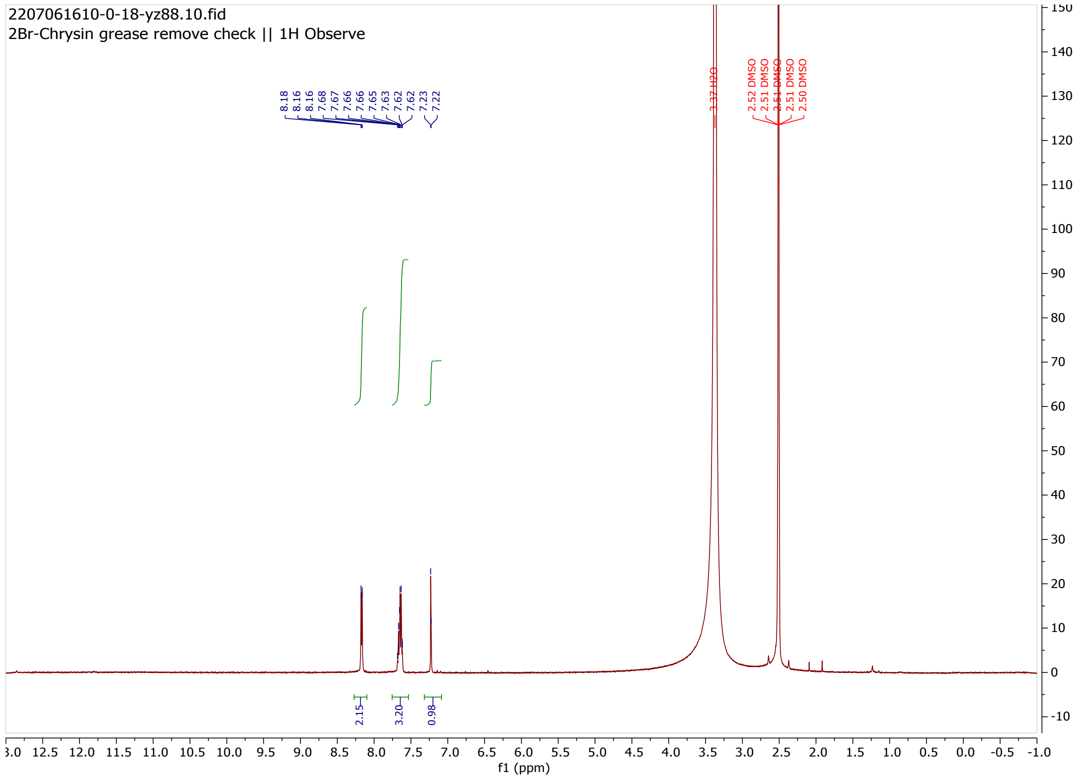

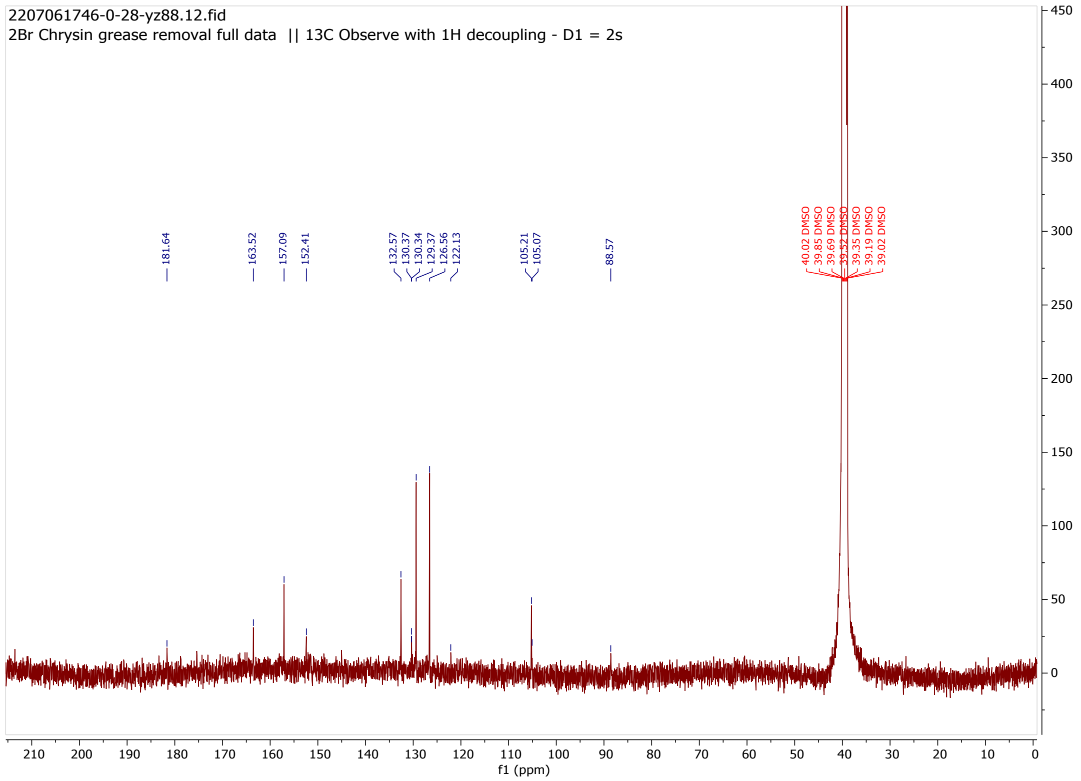

Figure S56. ^{1}H NMR (500 MHz, DMSO-d_6) and ^{13}C NMR (126 MHz, DMSO-d_6) of 6,8-dibromo-5,7-dihydroxy-2-phenyl-4H-chromen-4-one (**6c**).

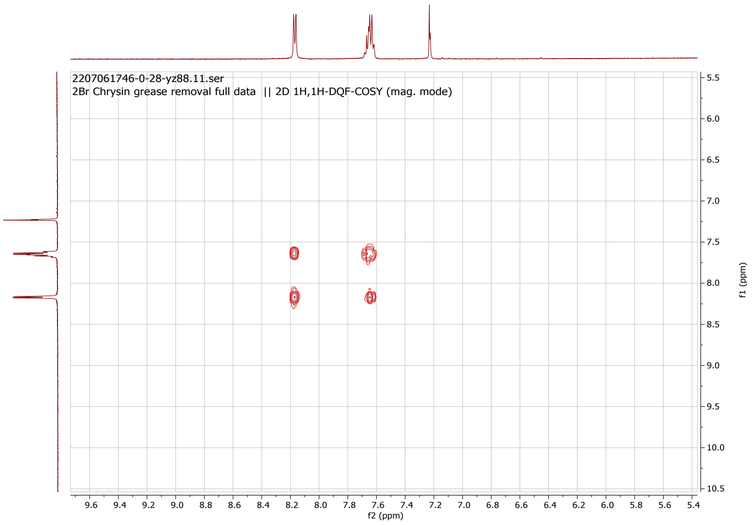

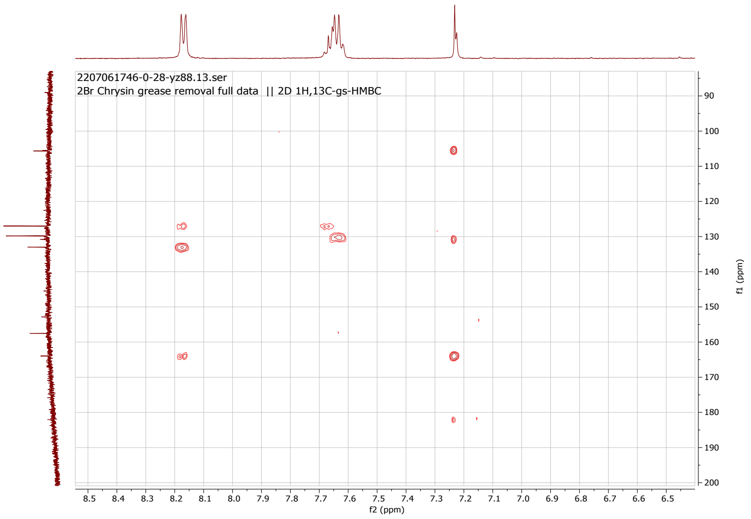

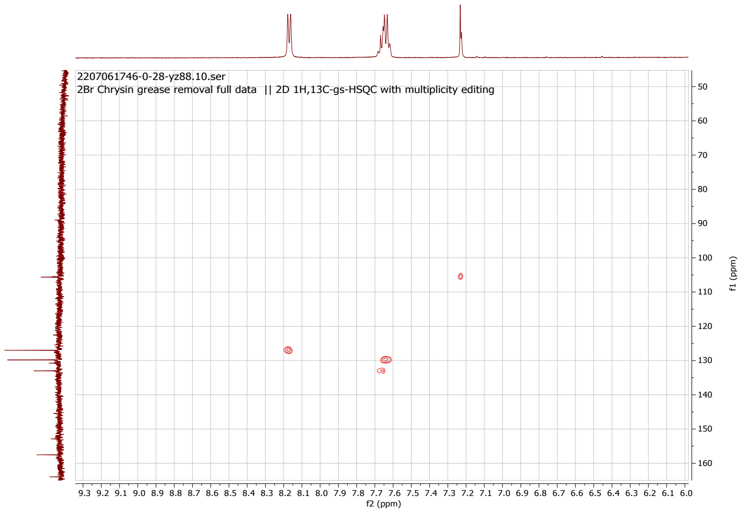

Figure S57. 2-D NMR (500 MHz, DMSO-d_6), COSY, HSQC and HMBC spectra of 6,8-dibromo-5,7-dihydroxy-2-phenyl-4H-chromen-4-one (**6c**).

2-Bromo-6-(isoxazol-5-yl)phenol (7a)

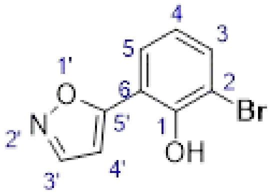

The substrate input of 16 mg of **7** was used, resulting in a total reaction volume containing 1-3 mM substrate concentration. Product extraction (3 times using ethyl acetate) followed by purification normal phase (100 % Hexane to 100 % DCM 20-30 column volumes as required). The resulting dried product (2.0 mg, 8.4 % yield) was dissolved in CD_3OD and characterised using ^{1}H NMR and ^{13}C NMR.

^{1}H NMR (500 MHz, CD_3OD) δ 8.43 (d, *J* = 1.8 Hz, 1H, H-3'), 7.82 (dd, *J* = 7.9, 1.7 Hz, 1H, H-5), 7.57 (dd, *J* = 7.9, 1.6 Hz, 1H, H-3), 7.05 (d, *J* = 1.8 Hz, 1H, H-4'), 6.79 (t, *J* = 7.9 Hz, 1H, H-4) ppm.

^{13}C NMR (126 MHz, CD_3OD) δ 167.5 (C-5'), 154 (C-1)*, 152.1 (C-3'), 135.2 (C-3), 127.5 (C-5), 120.1 (C-4), 118.3 (C-6), 114.5 (C-Br), 103.6 (C-4') ppm. * determined from HMBC.

HRMS (ESI$^+$) *m/z* chemical formula: $C_9H_6BrNO_2$; found 239.9631 $[M+H]^+$, expected 239.9655.

These data are consistent with the literature characterisation for this compound[23].

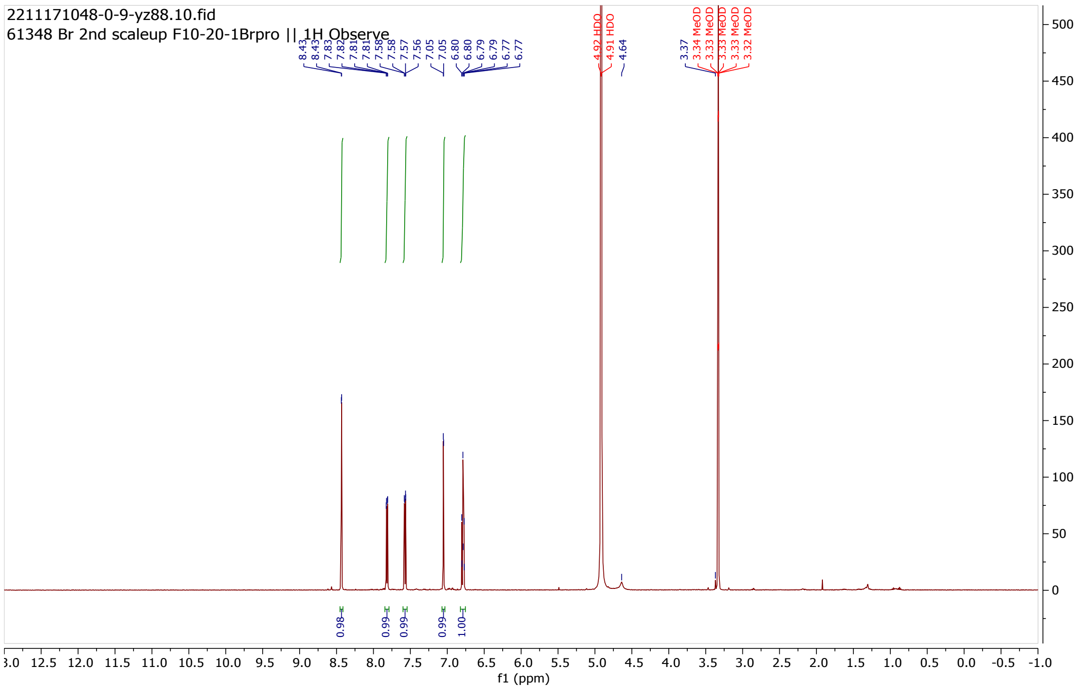

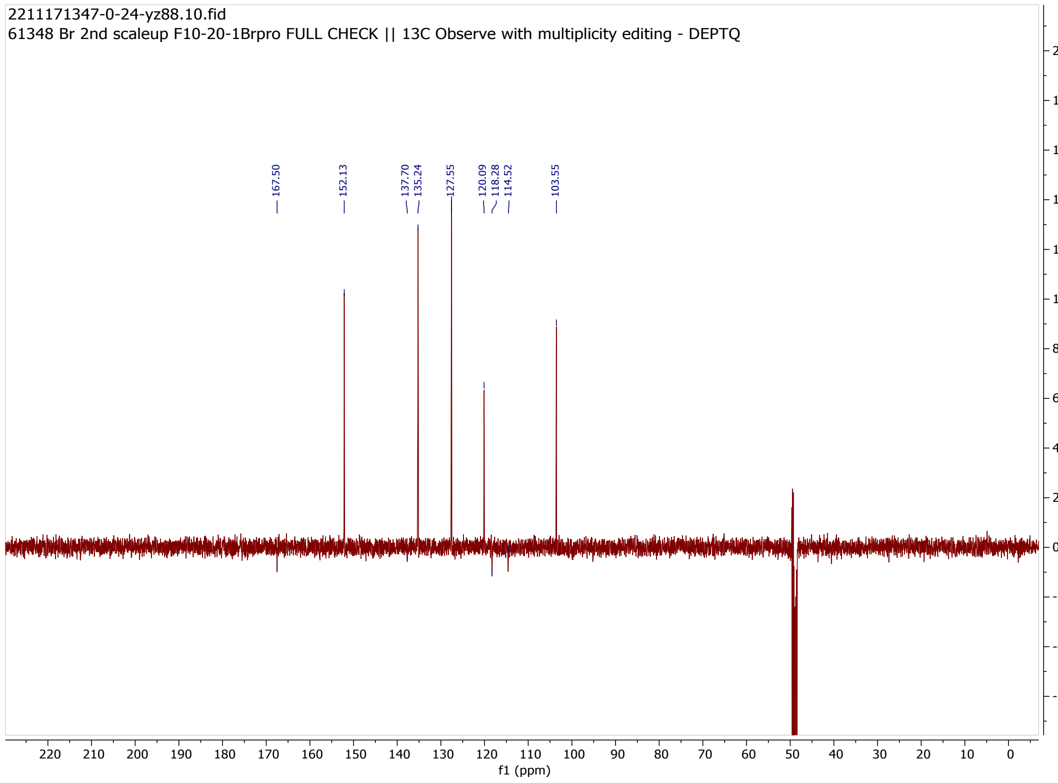

Figure S58. ^{1}H NMR (500 MHz, CD_3OD) and ^{13}C NMR (126 MHz, CD_3OD) of 2-(3-bromo-1H-indol-2-yl)aniline (**7a**).

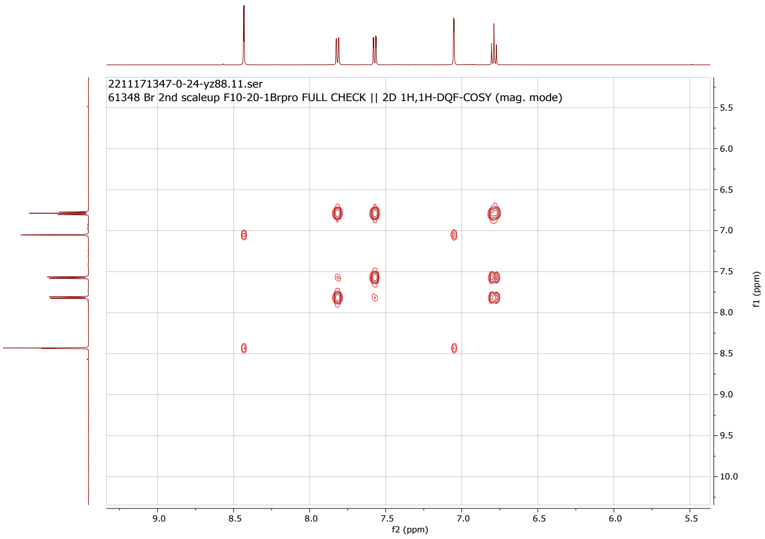

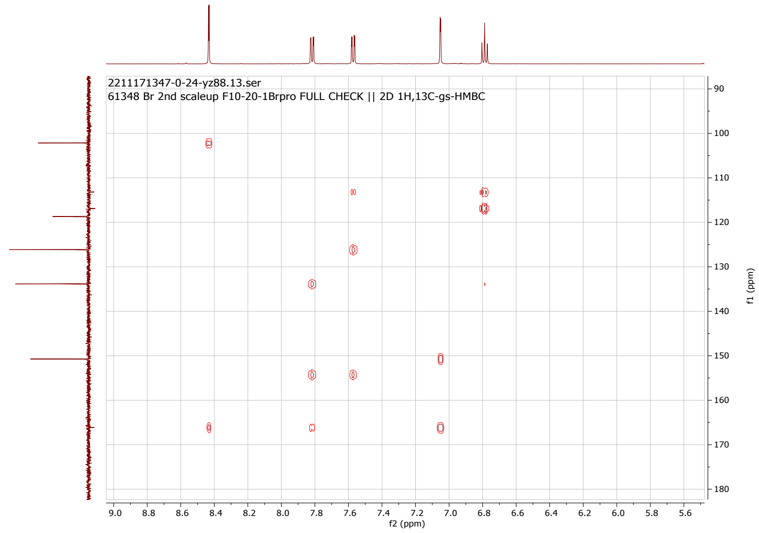

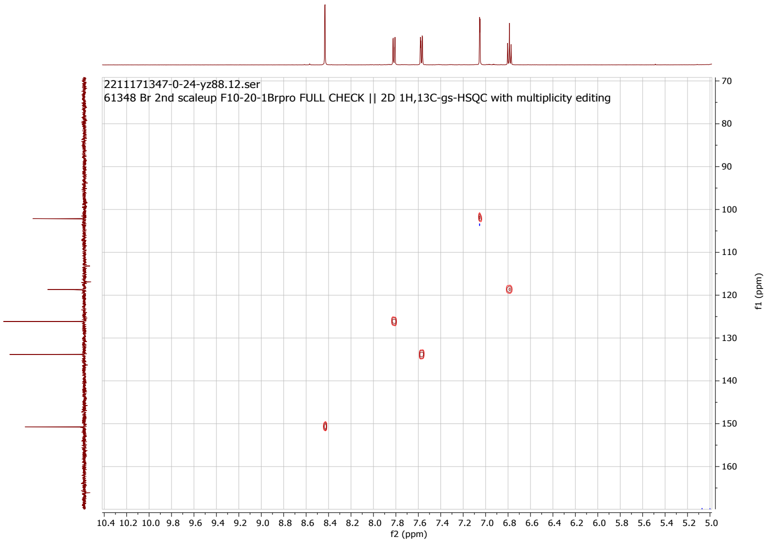

Figure S59. 2-D NMR (500 MHz, CD_3OD), COSY, HSQC and HMBC spectra of 2-(3-bromo-1H-indol-2-yl)aniline (**7a**).

4-Bromo-2-(isoxazol-5-yl)phenol (7b)

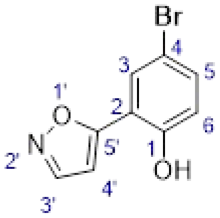

11b

This product was isolated from the same biotransformation and purification process as **7a**. The resulting dried product (1.0 mg, 3.4 % yield) was dissolved in CD^{3}OD and characterised using ^{1}H NMR and ^{13}C NMR.

^{1}H NMR (500 MHz, CD_3OD) δ 8.44 (d, J = 1.9 Hz, 1H, H-3'), 7.93 (d, J = 2.5 Hz, 1H, H-3), 7.39 (dd, J = 8.7, 2.5 Hz, 1H, H-5), 6.97 (d, J = 1.8 Hz, 1H, H-4'), 6.91 (d, J = 8.8 Hz, 1H, H-6) ppm.

^{13}C NMR (126 MHz, CD_3OD) δ 166.1 (C-5'), 155.0 (C-1),* 152.2 (H-3'), 134.8 (C-5), 130.4 (C-3), 119.7 (C-6), 119.6 (C-2), 110.6 (C-4).* 103.9 (C-4') ppm. * Determined from HMBC.

HRMS (ESI$^+$) *m/z* chemical formula: $C_9H_6BrNO_2$; found 239.9631 [M+H]$^+$, expected 239.9655.

These data are consistent with the literature characterisation for this compound[23].

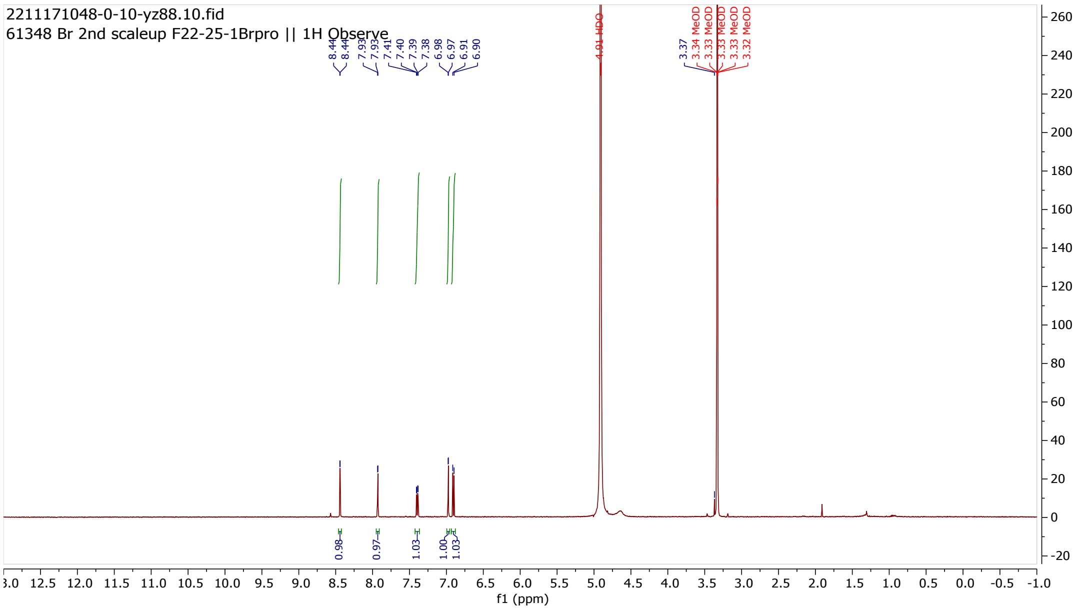

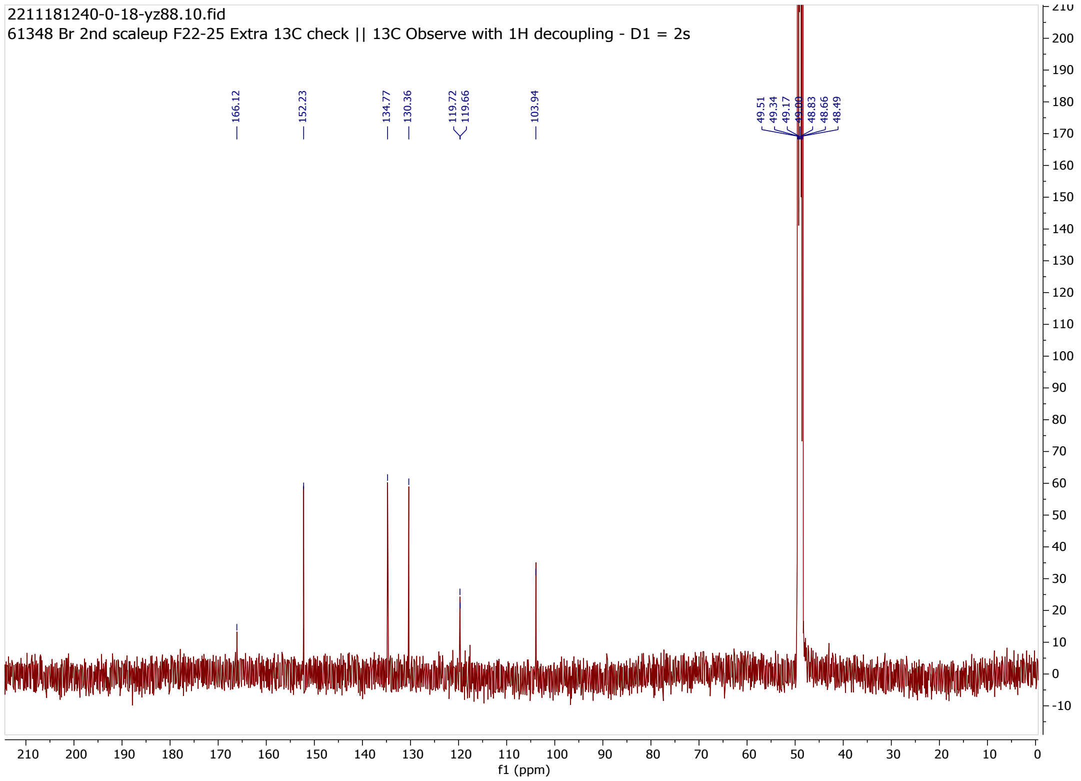

Figure S60. ^{1}H NMR (500 MHz, CD_3OD) and ^{13}C NMR (126 MHz, CD_3OD) of of 4-bromo-2-(isoxazol-5-yl)phenol (**7b**).

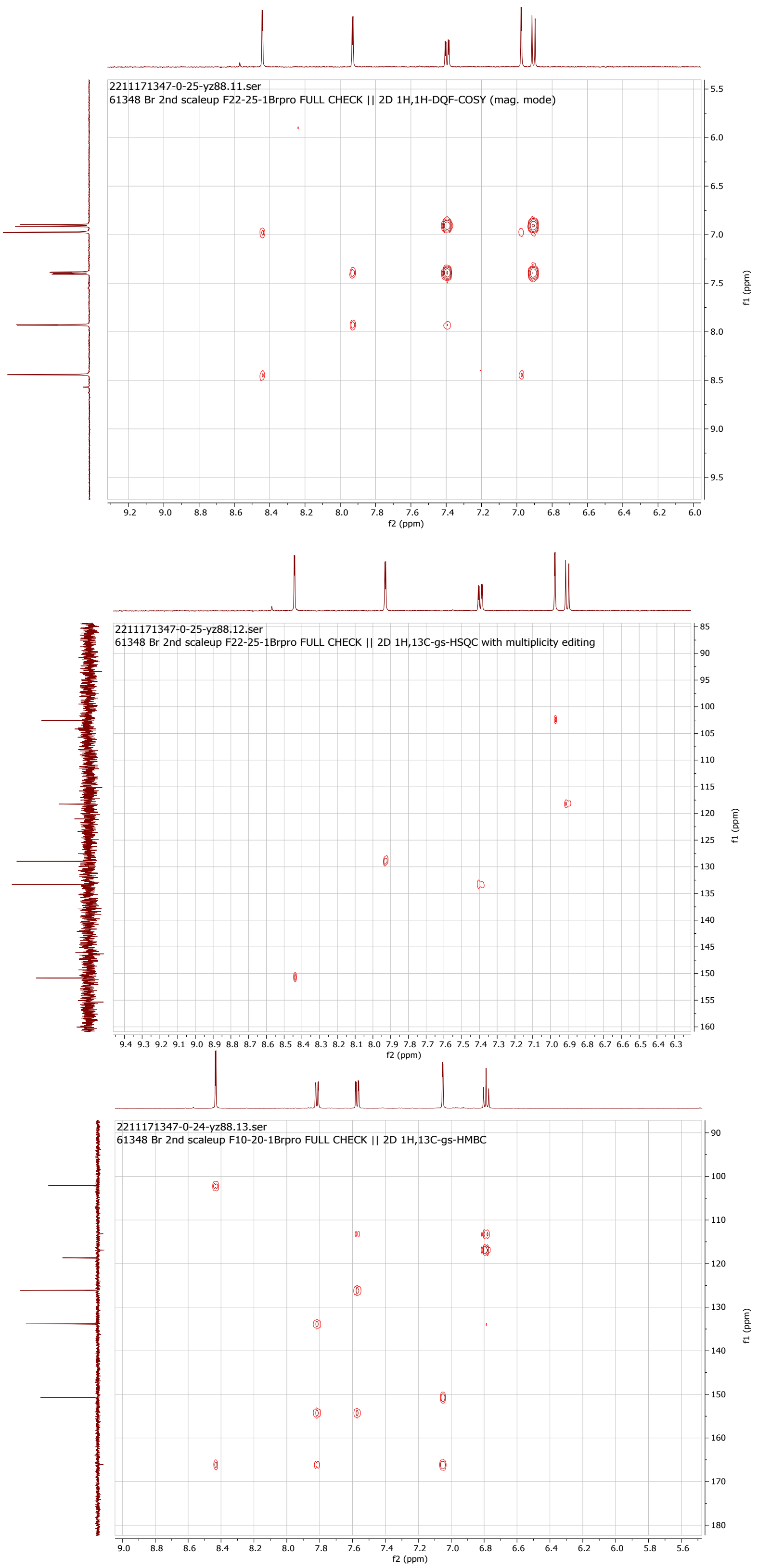

Figure S61. 2-D NMR (500 MHz, CD_3OD), COSY, HSQC and HMBC spectra of 4-bromo-2-(isoxazol-5-yl)phenol (**7b**).

2,4-Dibromo-6-(isoxazol-5-yl)phenol (7c)

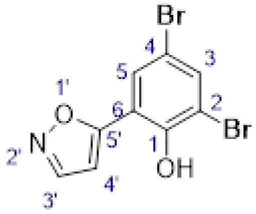

This product was isolated from the same biotransformation process and purification process described in the identification of **7a**. The resulting dried product (6.9 mg, 17.4 % yield) was dissolved in CD_3OD and characterised using ^{1}H NMR and ^{13}C NMR.

^{1}H NMR (500 MHz, CD_3OD) δ 8.48 (d, J = 1.9 Hz, 1H, H-3'), 7.95 (d, J = 2.4 Hz, 1H, H-5), 7.76 (d, J = 2.4 Hz, 1H, H-3), 7.03 (d, J = 1.9 Hz, 1H, H-4') ppm.

^{13}C NMR (126 MHz, CD_3OD) δ 165.3 (C-5'), 152.7 (C-1), 152.3 (C-3'), 137.0 (C-3), 130.0 (C-5), 119.5 (C-2), 114.2 (C-6), 112.3 (C-4), 104.9 (C-4') ppm.

HRMS (ESI$^+$) *m/z* chemical formula: $C_9H_8Br_2NO_2$; found 317.8773 $[M+H]^+$, expected 317.8760.

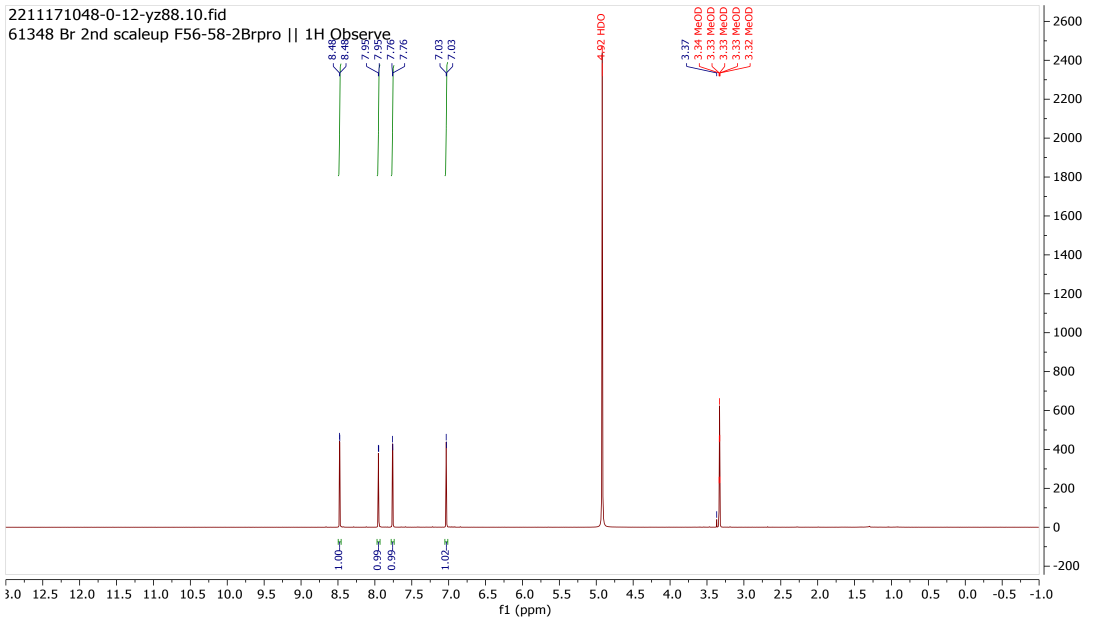

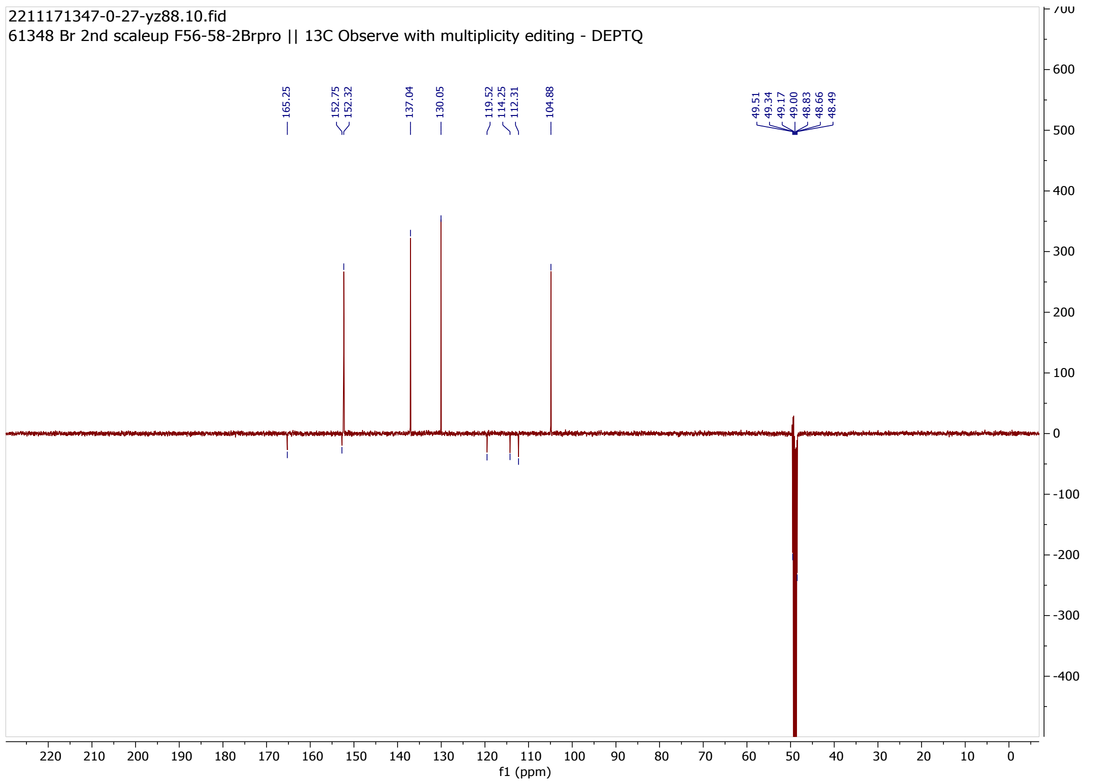

Figure S62. ^{1}H NMR (500 MHz, CD_3OD) and ^{13}C NMR (126 MHz, CD_3OD) of 2,4-dibromo-6-(isoxazol-5-yl)phenol (**7c**).

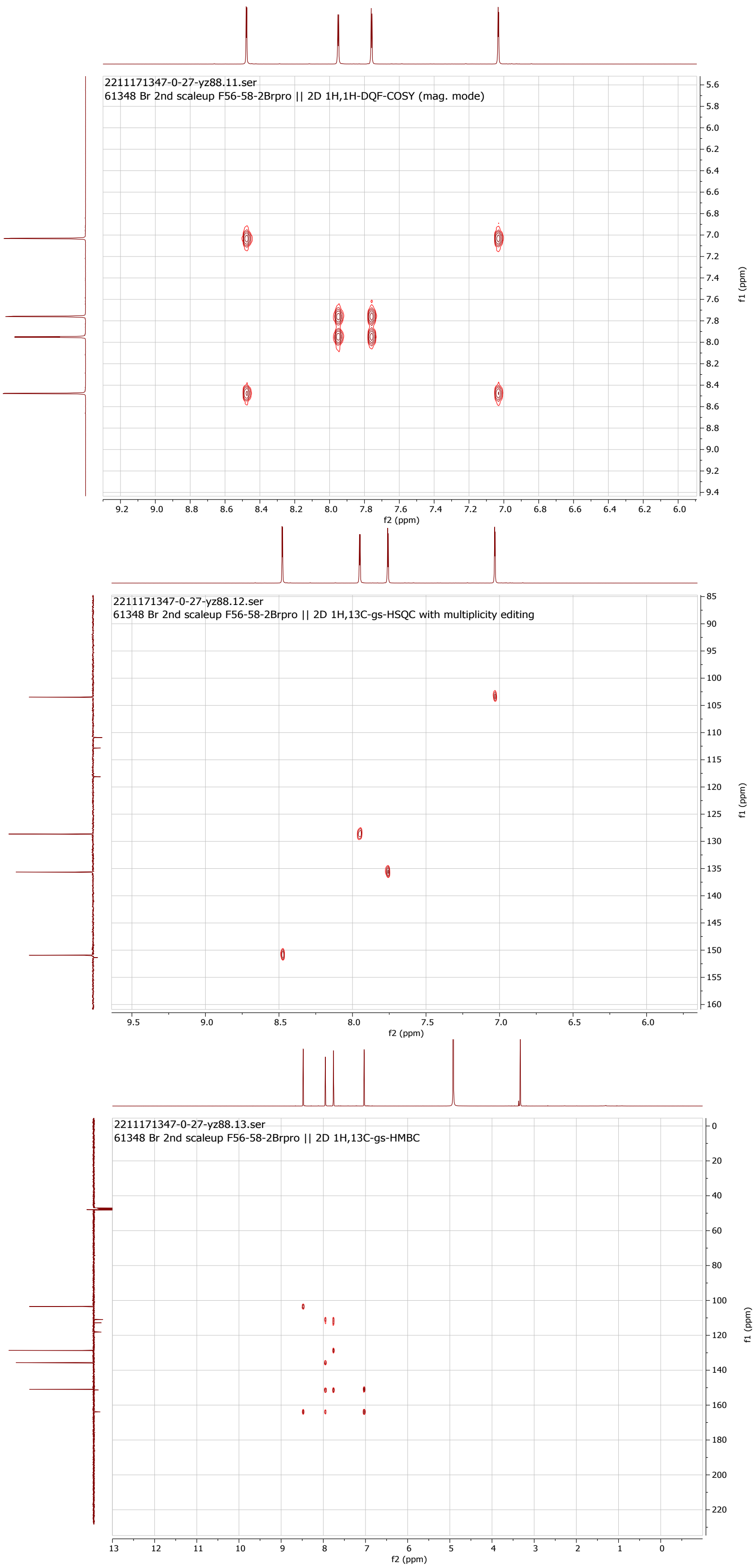

Figure S63. 2-D NMR (500 MHz, CD_3OD), COSY, HSQC and HMBC spectra of 2,4-dibromo-6-(isoxazol-5-yl)phenol (**7c**).

3.12 Thermal Shift Assay of CHEESY1

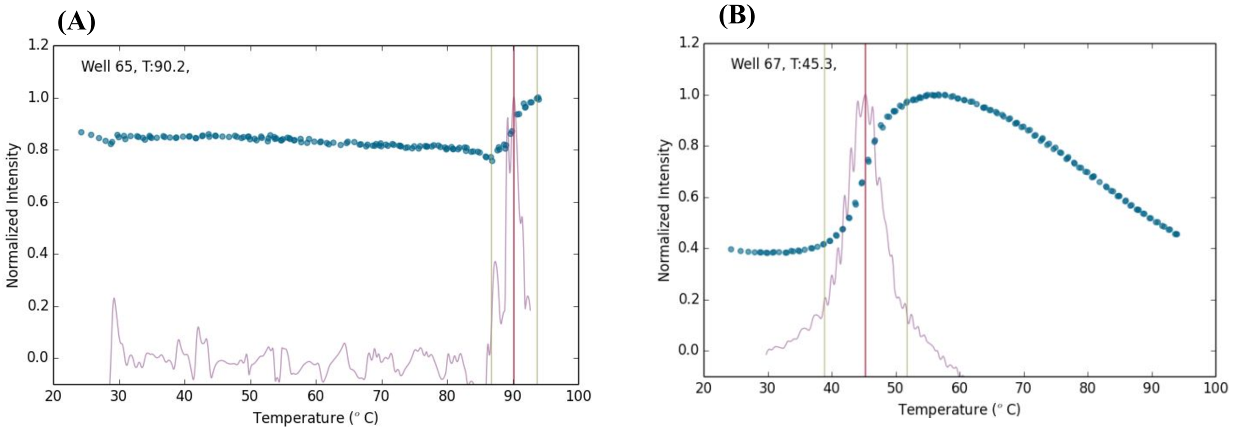

Figure S64. Fluorescence profiles of in 50 mM HEPES (pH 8.0) buffer only (A) and CHEESY1 in buffer. CHEESY1 (Tm = 45 ± 0.7°C) was tested.

3.13 Thermal Stability Analysis of CHEESY1

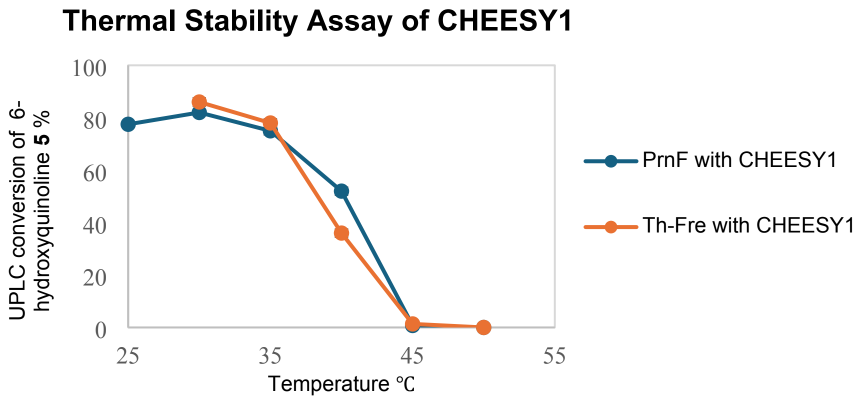

Figure S65. Thermal stability assay of CHEESY1 with PrnF and Th-Fre. Since Tm of CHEESY1 is shown as 45 °C， Th-Fre was used as the flavin reductase to test the thermal stability of CHEESY1, as Th-Fre shows its best working temperature is 45 °C. PrnF with CHEESY1 was tested also as a comparison.

3.14 Halogenation Activity Assays of Five Mutants against Selected Substrates

After the mutation constructs were generated, protein production and purification were performed according to the protocol described in Section 2.4. Purified protein was verified by SDS-PAGE (Figure S66). All five mutants were obtained as soluble proteins and purity was high. Halogenation assays were then conducted following the procedure in Section 2.6.

Y170I exhibited an approximately 40% reduction in conversion compared to CHEESY1 WT, Figure S67. Y170V showed relatively higher activity than Y170I, with only about 20% reduction compared to WT, and displayed no effect on the chlorination of substrate **2**. Results from both Y170I and Y170V indicated that replacing the phenolic tyrosine with hydrophobic residues affected enzymatic activity but did not completely abolish the activity. Notably, Y170I demonstrated poorer activity, likely due to steric hindrance caused by the additional methyl group interfering with substrate binding.

For the S311 mutations (Figure S67), S311A (hydroxyl deletion) moderately affected enzymatic activity, reducing the conversion of substrate **1** to approximately 50%, while having minimal impact on the bromination of substrate **2**. S311V completely abolished chlorination activity for substrate **2**, retaining only 40% bromination activity for this substrate, with less than 20% WT activity for bromination of substrates **1** and **6**. S311I resulted in complete loss of enzymatic activity, presumably due to steric hindrance preventing effective substrate binding after replacement of the hydroxyl group with *sec*-butyl chain.

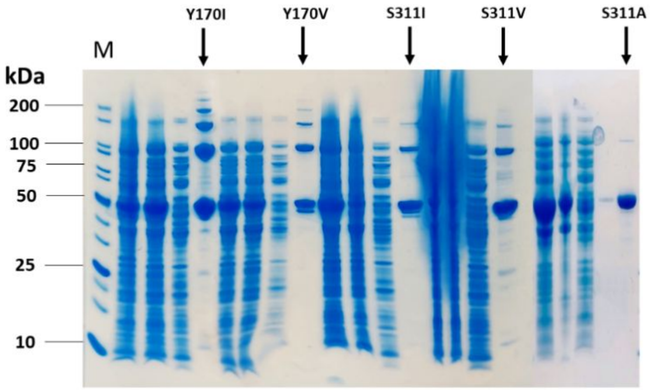

Figure S66. SDS-PAGE (10%) of Y170I, Y170V, S311I, S311V, and S311A. M: NEB unstained protein ladder 10-200 kDa. Eluted proteins are indicated by black arrows. All five mutants were soluble and showed ideal purity. .

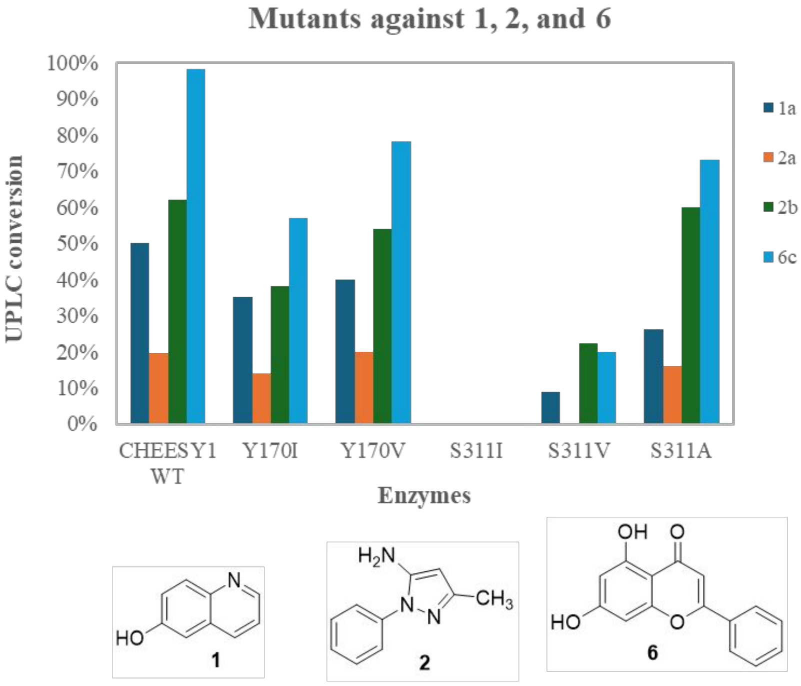

	UPLC conversion %*			
	1a	2a	2b	6c
CHEESY1 WT	50	20	62	98
Y170I	35	14	38	57
Y170V	40	20	54	78
S311I	0	0	0	0
S311V	8.7	0	22	20
S311A	26	16	60	73

*: % represents the product peak area ratio in UPLC chromatograms (measured at 254 nm for **1a**, 245 nm for **2a** and **2b**, and 300 nm for **6c**).

Figure S67. UPLC conversion of selected substrates by Y170I, Y170V, S311I, S311V, and S311A mutants compared to CHEESY1 WT. Substrates **1**, **2** and **6** were tested for bromination assays, additionally substrate **2** chlorination was tested.

4 References

1. Jumper, J.; Evans, R.; Pritzel, A.; Green, T.; Figurnov, M.; Ronneberger, O.; Tunyasuvunakool, K.; Bates, R.; Žídek, A.; Potapenko, A. Highly accurate protein structure prediction with AlphaFold. *Nature* **2021**, *596* (7873), 583-589.

2. Abramson, J.; Adler, J.; Dunger, J.; Evans, R.; Green, T.; Pritzel, A.; Ronneberger, O.; Willmore, L.; Ballard, A. J.; Bambrick, J. Accurate structure prediction of biomolecular interactions with AlphaFold 3. *Nature* **2024**, *630* (8016), 493-500.

3. Jurcik, A.; Bednar, D.; Byska, J.; Marques, S. M.; Furmanova, K.; Daniel, L.; Kokkonen, P.; Brezovsky, J.; Strnad, O.; Stourac, J. CAVER Analyst 2.0: analysis and visualization of channels and tunnels in protein structures and molecular dynamics trajectories. *Bioinformatics* **2018**, *34* (20), 3586-3588.

4. Design, L. Pharmacophore and ligand-based design with Biovia Discovery Studio®. *BIOVIA. California* **2014**.

5. Ravindranath, P. A.; Forli, S.; Goodsell, D. S.; Olson, A. J.; Sanner, M. F. AutoDockFR: advances in protein-ligand docking with explicitly specified binding site flexibility. *PLoS Comput. Biol.* **2015**, *11* (12), e1004586.
6. Ravindranath, P. A.; Sanner, M. F. AutoSite: an automated approach for pseudo-ligands prediction—from ligand-binding sites identification to predicting key ligand atoms. *Bioinformatics* **2016**, *32* (20), 3142-3149.
7. Davis, I. W.; Leaver-Fay, A.; Chen, V. B.; Block, J. N.; Kapral, G. J.; Wang, X.; Murray, L. W.; Arendall III, W. B.; Snoeyink, J.; Richardson, J. S. MolProbity: all-atom contacts and structure validation for proteins and nucleic acids. *Nucleic Acids Res.* **2007**, *35* (suppl_2), W375-W383.

8. Schrödinger，LLC. *The PyMOL Molecular Graphics System, Version 3.0.*

9. Wallace, A. C.; Laskowski, R. A.; Thornton, J. M. LIGPLOT: a program to generate schematic diagrams of protein-ligand interactions. *PEDS* **1995**, *8* (2), 127-134.
10. Morris, G. M.; Huey, R.; Lindstrom, W.; Sanner, M. F.; Belew, R. K.; Goodsell, D. S.; Olson, A. J. AutoDock4 and AutoDockTools4: Automated docking with selective receptor flexibility. *J. Comput. Chem.* **2009**, *30* (16), 2785-2791.
11. Trott, O.; Olson, A. J. AutoDock Vina: improving the speed and accuracy of docking with a new scoring function, efficient optimization, and multithreading. *J. Comput. Chem.* **2010**, *31* (2), 455-461.
12. Morris, G. M.; Goodsell, D. S.; Halliday, R. S.; Huey, R.; Hart, W. E.; Belew, R. K.; Olson, A. J. Automated docking using a Lamarckian genetic algorithm and an empirical binding free energy function. *J. Comput. Chem.* **1998**, *19* (14), 1639-1662.
13. Liu, H.; Naismith, J. H. An efficient one-step site-directed deletion, insertion, single and multiple-site plasmid mutagenesis protocol. *BMC Biotechnol.* **2008**, *8* (1), 1-10.
14. Menon, B. R. K.; Brandenburger, E.; Sharif, H. H.; Klemstein, U.; Shepherd, S. A.; Greaney, M. F.; Micklefield, J. RadH: A versatile halogenase for integration into synthetic pathways. *Angew. Chem. Int. Ed.* **2017**, *56* (39), 11841-11845.
15. Peh, G.; Gunawan, G. A.; Tay, T.; Tiong, E.; Tan, L. L.; Jiang, S.; Goh, Y. L.; Ye, S.; Wong, J.; Brown, C. J. Further characterization of fungal halogenase RadH and its homologs. *Biomol.* **2023**, *13* (7), 1081.
16. Crowe, C.; Molyneux, S.; Sharma, S. V.; Zhang, Y.; Gkotsi, D. S.; Connaris, H.; Goss, R. J. Halogenases: a palette of emerging opportunities for synthetic biology–synthetic chemistry and C–H functionalisation. *Chem. Soc. Rev.* **2021**, 50(17), 9443-9481.
17. Phintha, A.; Prakinee, K.; Jaruwat, A.; Lawan, N.; Visitsatthawong, S.; Kantiwiriyawanitch, C.; Songsungthong, W.; Trisrivirat, D.; Chenprakhon, P.; Mulholland, A. Dissecting the low catalytic capability of flavin-dependent halogenases. *J. Biol. Chem.* **2021**, *296*.
18. Clarke, D. D.; Gershon, H.; Shoja, M.; Yen, M.W. Revision of the assigned structures of 5- and 7-iodo-8-quinolinols and 5-and 7-iodo-2-methyl-8-quinolinols. *Monatsh. Chem.* **1998**, *129*, 419-422.
19. Allen, S.; Andrews, S. W.; Blake, J. F.; Condroski, K. R.; Haas, J.; Huang, L.; Jiang, Y.; Kercher, T.; Kolakowski, G. R.; Seo, J. Pyrrolidinyl urea and pyrrolidinyl thiourea compounds as trka kinase inhibitors. Google Patents, WO2012158413, 2012.

20. Barraja, P.; Diana, P.; Carbone, A.; Cirrincione, G. Nucleophilic reactions in the indole series: Displacement of bromine under phase transfer catalysis. *Tetrahedron* **2008**, *64* (51), 11625-11631.

21. Gershon, H.; Mcneil, M. W. 5-and 7-substituted 2-methyl-8-quinolinols. *J. Heterocycl. Chem.* **1972**, *9* (3), 659-667.

22. Park, H.; Dao, T. T.; Kim, H. P. Synthesis and inhibition of PGE2 production of 6, 8-disubstituted chrysin derivatives. *Eur. J. Med. Chem.* **2005**, *40* (9), 943-948.

23. Yalazan, H.; Barut, B.; Yıldırım, S.; Yalçın, C. Ö.; Kantekin, H. Axially disubstituted silicon (IV) phthalocyanines containing different isoxazolyl groups: Design, syntheses, binding and in vitro phototoxic activities against SH-SY5Y cells. *J. Mol. Struct*. **2022**, *1262*, 133066.
